# UA-30 ameliorates motor deficits through RalA-mediated mitophagy in ALS mice

**DOI:** 10.1172/JCI202787

**Published:** 2026-09-15

**Authors:** Bingge Zhang, Ye He, Ting Su, Xiaomei Li, Xiufen Zhang, Ruijuan Liu, Xiao Han, Ruiming Zhang, Chao Yang, Xinlei Liu, Qinghua Hou, Zaijun Zhang, Yongmei Xie, Gongping Liu, Xifei Yang

**Affiliations:** 1 Department of Pathophysiology, School of Basic Medicine and the Collaborative Innovation Center for Brain Science, Key Laboratory of Ministry of Education of China/Hubei Province for Neurological Disorders, Tongji Medical College, Huazhong University of Science and Technology, Wuhan, China.; 2 Shenzhen Key Laboratory of Modern Toxicology, Shenzhen Medical Key Discipline of Health Toxicology (2020–2024), Shenzhen Center for Disease Control and Prevention, Shenzhen, China.; 3 State Key Laboratory of Biotherapy, West China Hospital, Sichuan University and Collaborative Innovation Center for Biotherapy, Chengdu, China.; 4 Department of Neurology, Clinical Neuroscience Center, The Seventh Affiliated Hospital, Sun Yat-sen University, Shenzhen, China.; 5 State Key Laboratory of Bioactive Molecules and Druggability Assessment, Guangdong Basic Research Center of Excellence for Natural Bioactive Molecules and Discovery of Innovative Drugs, Institute of New Drug Research, College of Pharmacy, Jinan University, Guangzhou, China.; 6 Co-innovation Center of Neuroregeneration, Nantong University, Nantong, China.

**Keywords:** Cell biology, Neuroscience, ALS, Mitochondria

## Abstract

Amyotrophic lateral sclerosis (ALS) is a neurodegenerative disease characterized by progressive motor neuron loss, skeletal muscle atrophy, paralysis, and eventually death. Mitochondrial dysfunction plays a pivotal role in ALS pathogenesis, although the precise pathogenic mechanisms remain elusive, and effective therapeutic strategies are extremely limited. In this study, we developed a small-molecule inhibitor, UA-30, which directly targets RalA, and explored its potential for the treatment of ALS. We found that when administered via oral gavage for 6 weeks following the onset of motor deficit, UA-30 extended lifespan and improved motor function of SOD1^G93A^ mice, a model of ALS. UA-30 ameliorated motor neuron loss, neuroinflammation, fibrosis, and mitochondrial dysfunction, as evidenced by energy recovery, decreased oxidative stress, and enhanced mitophagy. Mechanistically, UA-30 inhibited RalA activity and thereby modulated ERK/FOXO3a signaling, which inhibited FOXO3a degradation via the ubiquitin-proteasome pathway; enhanced FOXO3a stability; and upregulated the expression of mitophagy-related genes in this ALS mouse model. The beneficial effects of UA-30 in ALS were abolished by overexpression of the constitutively active form of RalA (RalA^G23V^) or Mdivi-1 treatment. These findings support RalA inhibition as a therapeutic strategy for enhancing mitophagy and mitigating ALS-like pathology and support UA-30 as an orally active candidate for further preclinical development.

## Abstract 2

An orally administered compound, UA-30, improved movement and extended survival in an ALS mouse model by restoring mitochondrial quality control.

## Introduction

Amyotrophic lateral sclerosis (ALS) is a neurodegenerative disorder characterized by the selective degeneration of upper and lower motor neurons. As the disease progresses, patients gradually lose voluntary motor function and develop symptoms including dysphagia and respiratory insufficiency (1–3). ALS manifests in adulthood and exhibits rapid deterioration, with most patients succumbing to respiratory failure within 2–4 years of symptom onset (4).

Among Asian populations, the most prevalent mutation associated with ALS occurs in the superoxide dismutase 1 (*SOD1*) gene, accounting for approximately 2% of sporadic ALS and 10%–20% of familial ALS cases (5, 6). Currently, only 3 FDA-approved drugs are available for ALS treatment: riluzole, which reduces excitotoxicity, and the antioxidants edaravone and tofersen, which target *SOD1* mRNA to reduce the production of toxic SOD1 protein. However, these therapeutic agents offer only modest symptom alleviation and do not halt disease progression.

Evidence indicates the presence of mitochondrial dysfunction in both animal models of ALS and patients with ALS (7), which is associated with the misfolding and aggregation within mitochondria of proteins such as FUS, SOD1, and TDP-43 (8–10). Specifically, mutant SOD1 aggregates localized to the mitochondrial intermembrane space have been shown to impair electron transport chain (ETC) activity (9). Mitophagy, the selective autophagic degradation of damaged mitochondria, is essential for maintaining mitochondrial integrity and function, representing a critical quality control mechanism for compromised organelles (11, 12). In ALS, excessive mitochondrial damage coupled with impaired mitophagy disrupts mitochondrial turnover, leading to oxidative stress, protein aggregation, and ultimately neuronal degeneration (13, 14). Notably, activating autophagy has been shown to enhance the survival of motor neurons in ALS models (15). Collectively, these findings highlight the importance of neuronal mitophagy signaling pathways in slowing ALS progression.

Urolithin A (UA) is a metabolite produced by the gut microbiota following the consumption of polyphenol-rich plant-based foods. It has been demonstrated to activate mitophagy, thereby contributing to the maintenance of healthy cellular function (16, 17). However, the absorption of UA is highly dependent on the gut microbiota, dietary patterns, and health status. Furthermore, the rigid structure of UA substantially reduces its water solubility and lipophilicity, resulting in limited oral bioavailability and gastrointestinal membrane permeability. These factors collectively restrict the clinical translation of UA (18–20).

To overcome these limitations, we generated a UA-derived small-molecule compound, UA-30 (Supplemental Figure 1; supplemental material available online with this article; https://doi.org/10.1172/JCI202787DS1), by replacing the phenolic hydroxyl group of UA with an *N*-methylpiperazine moiety followed by hydrochloride salt formation. In prior independent characterization, UA-30 showed improved pharmacokinetic properties compared with UA. Based on these properties, the present study was designed to evaluate the therapeutic potential of UA-30 in the SOD1^G93A^ transgenic mouse model of ALS. RalA was further investigated as a potential molecular target of UA-30 using complementary assays in this study.

## Results

### UA-30 improved motor performance and extended lifespan in SOD1^G93A^ mice.

To evaluate the CNS distribution of UA-30, pharmacokinetic analysis was performed after a single oral dose in mice. UA-30 was detected in both brain and spinal cord. The *C_max_* in spinal cord reached 6,293 ± 1,022 ng/g at 0.625 h after dose, while the *C_max_* in brain reached 16,490 ± 2,204 ng/g at 0.875 h after dose. UA-30 also showed favorable CNS distribution (brain/plasma, >2.5; spinal cord/plasma, >0.7), together with relatively prolonged tissue half-lives (brain *t_1/2_* = 3.44 h; spinal cord *t_1/2_* = 3.67 h) (Supplemental Figure 2 and Supplemental Table 1).

**Figure 1:**
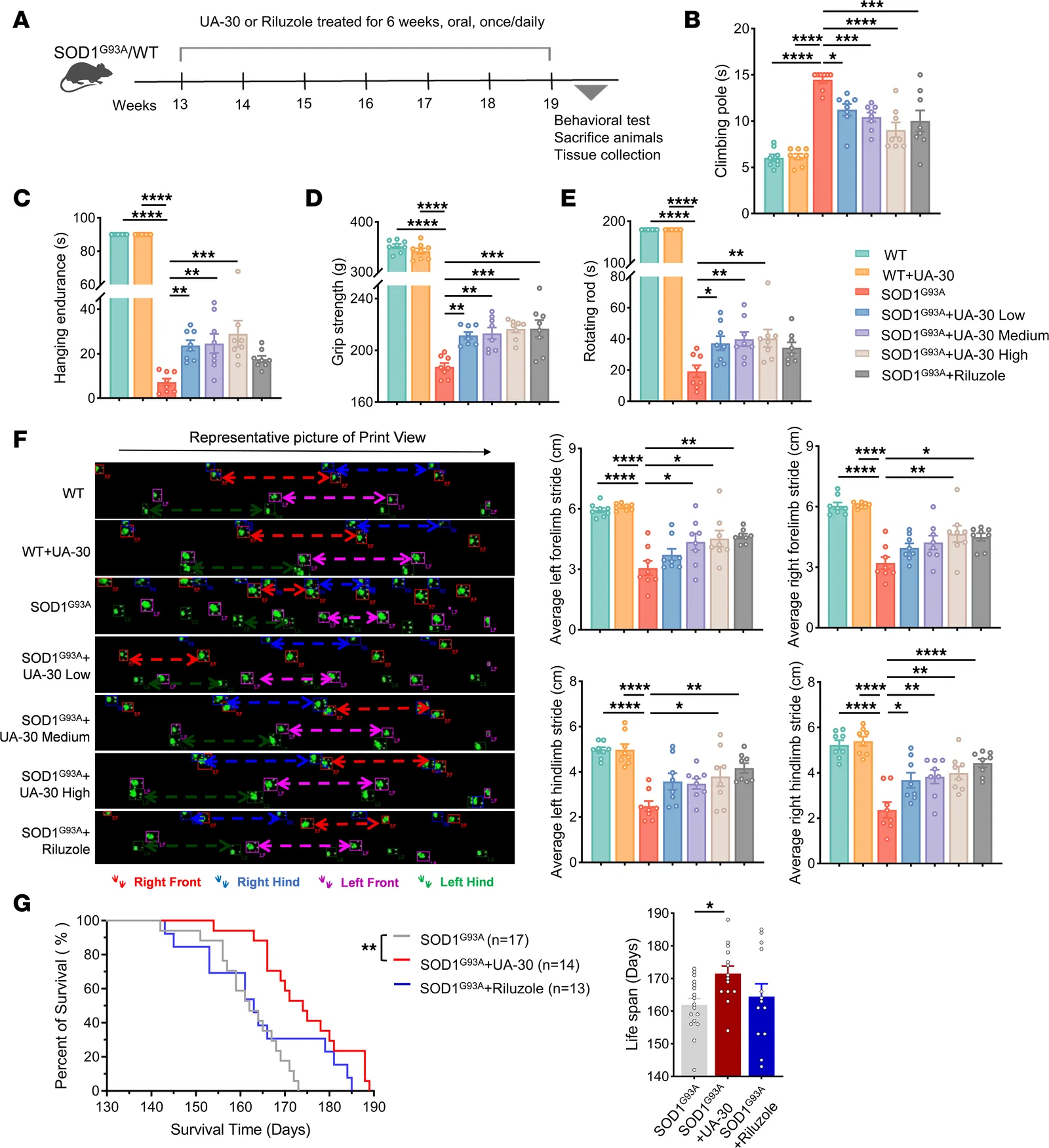
UA-30 treatment improved motor dysfunction and increased lifespan in male SOD1^G93A^ mice. (**A**) Schematic of the experimental protocol and administration schedule. (**B**–**F**) Motor performance and muscular strength assessments in male SOD1^G93A^ mice treated with different doses of UA-30 or saline for 6 weeks. Tests performed: pole climbing (**B**), wire hanging (**C**), grip strength (**D**), rotarod (**E**), and gait (**F**). Representative marker paw prints in gait analysis and statistics on left/right forelimb and left/right hind limb stride length. *n* = 8 male mice per group. (**G**) Kaplan-Meier survival curves for male SOD1^G93A^ mice treated with saline, UA-30 (100 mg/kg), or riluzole (10 mg/kg). Statistical summaries show overall lifespan for each cohort. *n* = 17 SOD1^G93A^ male mice. *n* = 14 SOD1^G93A^ + UA-30 male mice, *n* = 13 SOD1^G93A^ + riluzole male mice. Data are expressed as mean ± SEM. *P < 0.05, **P < 0.01, ***P < 0.001, and ****P < 0.0001. **B**–**F**, 1-way ANOVA followed by Tukey’s multiple-comparison test; **G**, log-rank (Mantel-Cox) test for survival curves and 1-way ANOVA followed by Tukey’s multiple-comparison test for overall lifespan summary.

Next, we evaluated the effects of UA-30 on ALS disease progression and survival in SOD1^G93A^ mice. SOD1^G93A^ transgenic mice at 13 weeks of age received oral administration of UA-30 (25, 50, and 100 mg/kg) or riluzole (10 mg/kg) once daily for 6 weeks (Figure 1A). Baseline behavioral assessments before treatment initiation showed comparable motor function across all experimental groups (Supplemental Figure 3A). Longitudinal behavioral assessments such as pole climbing, wire hanging, and grip strength were performed at weeks 2, 4, and 6 during the 6-week treatment period to evaluate the effects of UA-30 on motor function (Figure 1, B–D, Supplemental Figure 2, B and C, and Supplemental Figure 4, A–C). At the week 6 endpoint, motor coordination and balance were further evaluated using the rotarod test (Figure 1E and Supplemental Figure 4D), and locomotor patterns were assessed by gait analysis (Figure 1F, Supplemental Figure 4E, and Supplemental Videos 1 and 2). Compared with control mice, SOD1^G93A^ mice exhibited impaired climbing ability, endurance, and grip strength as the disease advanced, with males showing a faster rate of disease progression than females (Figure 1, B–D, Supplemental Figure 3, B and C, and Supplemental Figure 4, A–C). UA-30 treatment attenuated the decline in motor performance in both sexes across multiple behavioral endpoints. In male mice, UA-30 reduced pole descent time, prolonged wire-hang latency, increased grip strength, and improved rotarod performance (Figure 1, B–E). Female mice also showed improvement in these behavioral measures (Supplemental Figure 4, A–D). Gait analysis showed that SOD1^G93A^ mice had substantially reduced stride lengths compared with WT controls, while high-dose UA-30 treatment increased the average stride length in male mice (Figure 1F and Supplemental Video 1) and improved specific stride parameters in female mice (Supplemental Figure 4E and Supplemental Video 2). Specifically, compared with the low dose, the high dose improved performance by 19.36% in the pole-climbing test and increased hanging latency by 22.11%, grip strength by 2.30%, and rotarod latency by 8.39% (Figure 1, B–E). Notably, high-dose UA-30 demonstrated superior efficacy to riluzole treatment in improving most ALS-associated motor deficits, shown as more pronounced improvements in pole descent time (reducing time by 13.7% in females and 10.6% in males), wire hanging endurance (increasing time by 39.7% in females and 64.5% in males), and rotarod fall latency (increasing latency by 29.7% in females and 14.9% in males) (Figure 1, B, C, and E, and Supplemental Figure 4, A, B, and D).

To further determine whether treatment responses differed by sex, we performed 2-way ANOVA using sex and treatment as factors for the week 6 behavioral endpoints. No significant sex × treatment interaction was detected for pole climbing (*P* = 0.3222), wire hanging (*P* = 0.5612), grip strength (*P* = 0.8287), or rotarod performance (*P* = 0.5821) (Supplemental Figure 5, A–D), indicating that there was no statistical evidence for sex-specific treatment responses in these assays. Significant main effects of treatment were observed for all 4 behavioral endpoints, whereas sex main effects were detected for wire hanging (*P* < 0.0001), grip strength (*P* = 0.0326), and rotarod performance (*P* = 0.0118), but not for pole climbing (*P* = 0.3674) (Supplemental Figure 5, A–D), reflecting endpoint-dependent overall differences between males and females rather than differential treatment responses.

To address whether the structural modification of UA-30 confers a therapeutic advantage over its parent compound, we performed a head-to-head comparison at equimolar doses in SOD1^G93A^ mice. Functional assessment in SOD1^G93A^ mice treated with UA-30 revealed improvements in motor performance. UA-30 demonstrated greater improvements in pole-climbing descent time, hanging endurance, and rotarod performance (Supplemental Figure 6, A–C), whereas grip strength did not differ significantly between the 2 groups (Supplemental Figure 6D).

To investigate the effect of UA-30 on survival in SOD1^G93A^ mice, we established cohorts treated with UA-30, riluzole, or vehicle control. Compared with untreated SOD1^G93A^ mice, UA-30–treated SOD1^G93A^ mice showed a longer mean overall lifespan (171.5 ± 8.7 days vs. 161.9 ± 8.1 days, *P* = 0.027) and an 8.5-day increase in median survival. Although the difference in survival distribution between UA-30 and riluzole groups did not reach statistical significance (*P* = 0.107), the UA-30 group showed a trend toward longer survival, as indicated by a greater mean overall lifespan (171.5 ± 8.7 vs. 164.5 ± 14.2 days) and median survival (170.5 vs. 163 days) (Figure 1G). Survival was also analyzed in the female cohort. However, under the present study conditions, the survival benefit of UA-30 did not reach statistical significance (Supplemental Figure 4F).

### UA-30 reduced muscle damage and fibrosis in the gastrocnemius muscle of SOD1^G93A^ mice.

**Figure 2:**
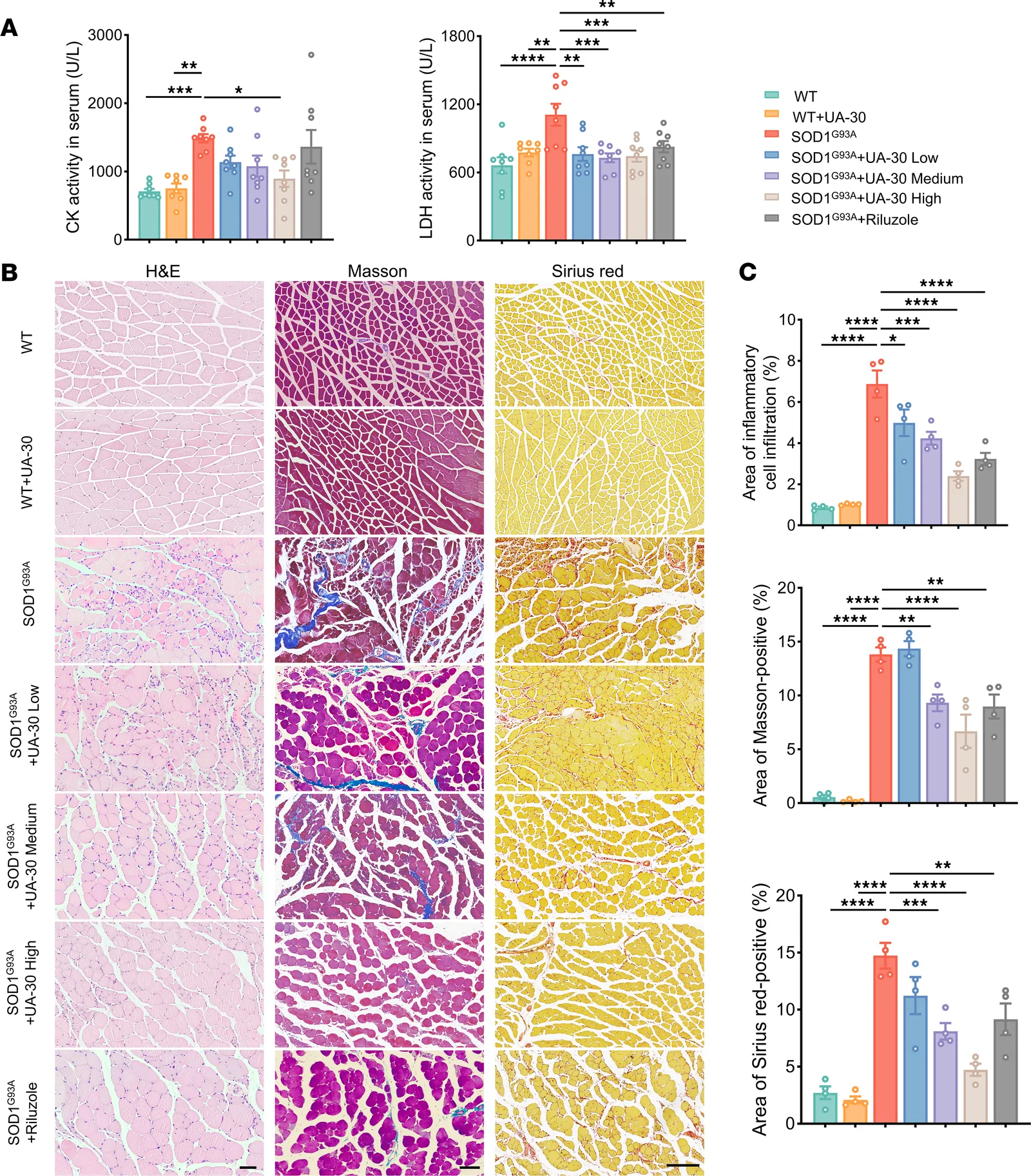
UA-30 treatment alleviated muscle damage and suppressed muscle pathologic deterioration of male SOD1^G93A^ mice. Samples were collected at the experimental endpoint after 6 weeks of treatment (19 weeks of age) from the same cohort of mice used for behavioral assessments. (**A**) Blood biochemical analysis of creatine kinase (CK) and lactate dehydrogenase (LDH) activity in SOD1^G93A^ male mice treated with UA-30 or saline in each group. *n* = 8 male per group. (**B** and **C**) Representative H&E, Masson’s, and Sirius red staining of gastrocnemius muscle from male SOD1^G93A^ mice after UA-30 treatment, with corresponding quantification of inflammatory area, collagen fiber content, and collagen deposition. Scale bars: 50 μm (H&E), 100 μm (Masson), 200 μm (Sirius red). *n* = 4 male per group. Data are expressed as mean ± SEM. *P < 0.05, **P < 0.01 ***P < 0.001, and ****P < 0.0001. 1-way ANOVA followed by Dunnett’s multiple-comparison test.

At the end of the 6-week treatment period, biochemical and histopathological analyses were performed on the same cohort of mice used for behavioral assessments. Oral administration of different doses of UA-30 did not affect the body weight of SOD1^G93A^ mice (Supplemental Figure 7A). Compared with control mice, SOD1^G93A^ mice exhibited a statistically significant reduction in gastrocnemius muscle weight (*P* < 0.0001). Notably, UA-30 treatment increased gastrocnemius muscle weight in female SOD1^G93A^ mice (Supplemental Figure 7B). Furthermore, UA-30 reduced serum creatine kinase and lactate dehydrogenase activities (Figure 2A) and normalized the reduced blood glucose levels observed in SOD1^G93A^ mice (Supplemental Figure 8, A and B). Riluzole treatment was associated with increased serum alanine aminotransferase and aspartate aminotransferase levels in SOD1^G93A^ mice (Supplemental Figure 8A).

In ALS, atrophy of the gastrocnemius muscle is frequently accompanied by fibrosis (21). Treatment with UA-30 reduced the inflammatory area and collagen fiber hyperplasia in the gastrocnemius muscle of SOD1^G93A^ mice (Figure 2, B and C). Masson’s and Sirius red staining revealed substantially higher levels of muscular fibrosis in untreated SOD1^G93A^ mice compared with WT mice. UA-30 administration attenuated gastrocnemius fibrosis in this model, which was particularly evident at the high dose of UA-30. Compared with riluzole, high-dose UA-30 showed numerically lower fibrotic areas (Masson-positive area: 6.7% ± 3.1% vs. 9.0% ± 2.2%; Sirius red–positive area: 4.7% ± 1.1% vs. 9.2% ± 2.8%), although no statistically significant difference was detected between the 2 groups. In WT mice, synaptophysin-positive presynaptic terminals showed extensive overlap with α-bungarotoxin–labeled motor end plates, indicating intact neuromuscular junction (NMJ) innervation. By contrast, SOD1^G93A^ mice exhibited evident NMJ denervation. UA-30 treatment partially restored NMJ innervation in SOD1^G93A^ mice (Supplemental Figure 9).

### UA-30–attenuated motor neuron loss and glial cell proliferation in SOD1^G93A^ mice.

**Figure 3:**
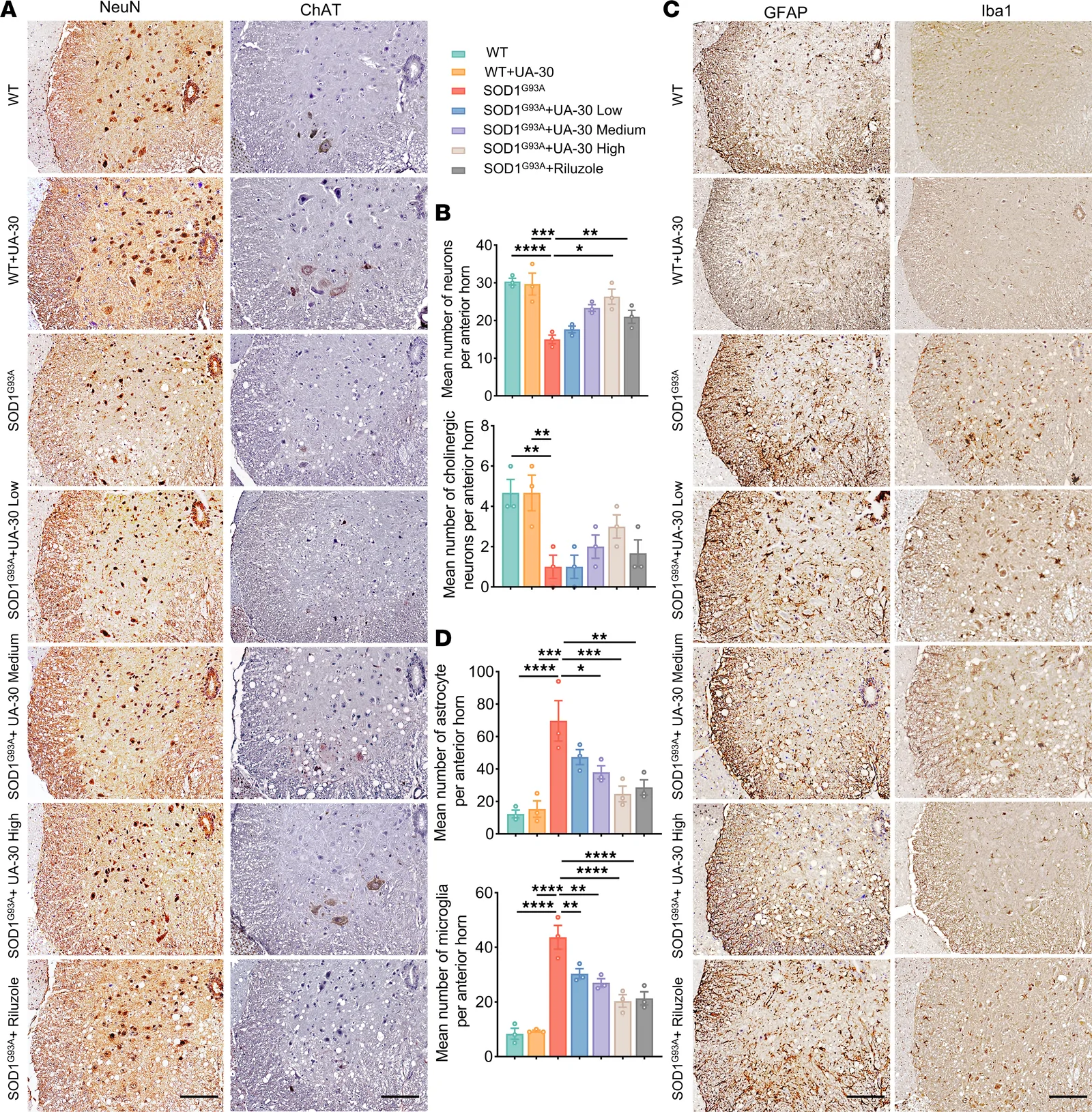
UA-30 treatment attenuated motor neuron loss and neuroinflammation in male SOD1^G93A^ mice. (**A** and **B**) Representative images (**A**) and statistical analysis (**B**) of immunohistochemical staining of neurons (NeuN) and cholinergic neurons labeled with choline acetyltransferase (ChAT) of L4-L5 spinal cord from saline- or UA-30–treated male SOD1^G93A^ mice. Scale bar: 200 μm. (**C** and **D**) Representative images (**C**) and quantification (**D**) of astrocytes (GFAP) and microglia (Iba1) immunohistochemical staining in spinal cord after UA-30 treatment in male SOD1^G93A^ mice. Scale bar: 200 μm. *n* = 3 male per group. Data are expressed as mean ± SEM. *P < 0.05, **P < 0.01 ***P < 0.001, and ****P < 0.0001. 1-way ANOVA followed by Dunnett’s multiple-comparison test.

The principal neuropathological hallmark of ALS is the progressive loss of motor neurons (1). To assess motor neuron survival within the anterior horn of the L4-L5 lumbar spinal cord, neurons and motor neurons were specifically labeled using NeuN and choline acetyltransferase immunohistochemistry, respectively. SOD1^G93A^ mice showed a statistically significant reduction in the number of spinal motor neurons compared with controls (*P* < 0.0001). UA-30 administration ameliorated loss of spinal motor neurons in the ALS model (Figure 3, A and B, and Supplemental Figure 10A). We also found that UA-30 treatment attenuated the increase in both astrocyte and microglial cell numbers observed in the spinal cord of male SOD1^G93A^ mice (Figure 3, C and D). Similar results were observed in the spinal cord of female SOD1^G93A^ mice (Supplemental Figure 10A). High-dose UA-30 treatment proved superior to riluzole, exhibiting a 20.3% greater preservation of neurons in male mice and a 9.5% greater preservation in female mice compared with the riluzole-treated group.

To comprehensively evaluate the pathogenesis of ALS, human SOD1 (hSOD1) levels were quantified in the medulla oblongata and spinal cord of SOD1^G93A^ mice (Supplemental Figure 10B). We found that high-dose UA-30 administration reduced hSOD1 levels in both the medulla and spinal cord of SOD1^G93A^ mice. These results indicate that UA-30 treatment mitigates the extent of cellular pathology associated with ALS.

### UA-30 modified the protein expression profile in SOD1^G93A^ mice.

To elucidate the therapeutic mechanism of UA-30, we performed proteomic analyses of the medulla oblongata and spinal cord in SOD1^G93A^ mice. A total of 6,266 proteins were identified and quantified in spinal cord samples, while 5,893 proteins were detected in the medulla oblongata. Compared with WT mice, SOD1^G93A^ mice exhibited 1,364 differentially expressed (DE) proteins in the spinal cord (Supplemental Figure 11A). Bioinformatic analysis revealed that downregulated proteins were enriched in biological processes including mitochondrial ATP synthesis, aerobic respiration, TCA cycle, mitochondrial respiratory chain assembly, and mitochondrial electron transport (Supplemental Figure 11B). In the medulla oblongata, 653 DE proteins were identified in SOD1^G93A^ mice compared with WT controls (Supplemental Figure 11C), with downregulated DE proteins primarily involved in endocytosis, protein transport, mitochondrial ATP synthesis, aerobic respiration, and mitochondrial respiratory chain assembly (Supplemental Figure 11D). These results indicate marked energy metabolism dysregulation and mitochondrial dysfunction in ALS model mice, suggesting that improving mitochondrial function represents a potential therapeutic strategy for ALS.

**Figure 4:**
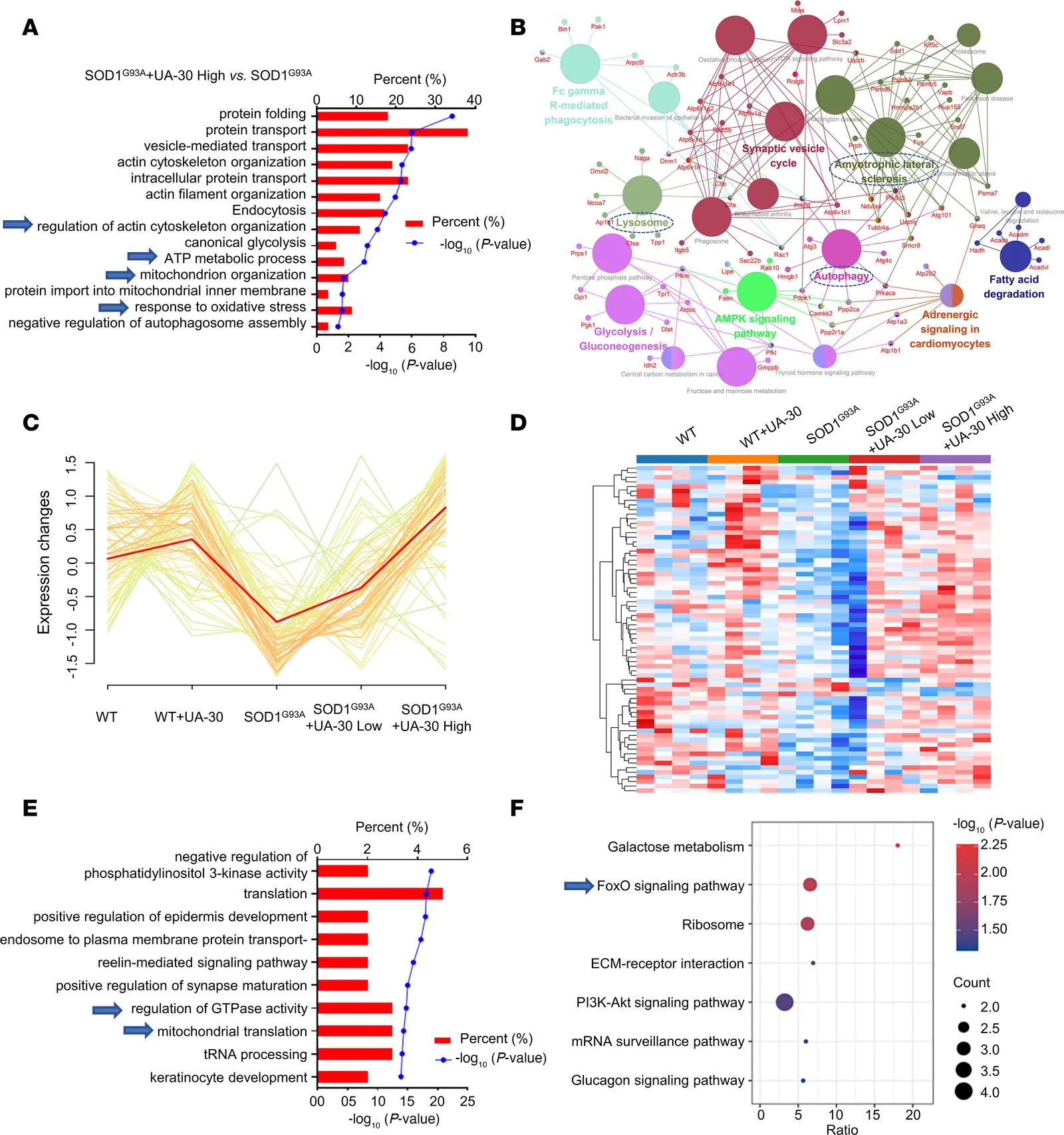
Proteomics analysis of the spinal cord and medulla oblongata in male SOD1^G93A^ mice after UA-30 treatment. (**A** and **B**) Corresponding biological processes (**A**) and KEGG pathways (**B**) of DE protein enrichment in medulla oblongata proteomics under high-dose UA-30 treatment, with *P* value of enrichment less than 0.05. (**C**) An ameliorated expression pattern in the spinal cord proteome identified by unsupervised hierarchical clustering of DE proteins based on their normalized expression profiles across experimental groups. The red line indicates the mean expression trend of this cluster. (**D**) Heatmap showing the expression profiles of 72 DE proteins belonging to the ameliorated expression cluster shown in (**C**), which decreased in SOD1^G93A^ mice and was restored toward WT levels after UA-30 treatment. Rows represent individual proteins, columns represent experimental groups, and colors indicate row-wise *z* score–normalized protein abundance (red, higher expression; blue, lower expression). (**E** and **F**) Biological processes (**E**) and KEGG pathways (**F**) enriched among the DE proteins within this expression pattern. Enrichment was defined as *P* value < 0.05. Arrows indicate terms/pathways with high enrichment scores and greater biological relevance.

Gene Ontology analysis of DE proteins in the medulla oblongata demonstrated that high-dose UA-30 treatment enriched biological processes related to actin cytoskeleton organization regulation, canonical glycolysis, ATP metabolism, mitochondrial organization, oxidative stress response, and autophagosome assembly in SOD1^G93A^ mice compared with untreated model mice (Figure 4A). KEGG pathway analysis of the relevant proteins implicated pathways primarily associated with ALS, autophagy, lysosomes, and glycolysis (Figure 4B). Subsequent unsupervised hierarchical clustering was performed on the 269 DE proteins identified in the spinal cord to group proteins based on their normalized expression profiles across all experimental conditions. One cluster displaying an ameliorated expression pattern following UA-30 treatment was identified (Figure 4C). A heatmap of the 72 proteins within this cluster is shown (Figure 4D). Gene enrichment analysis of these 72 proteins revealed their involvement in biological processes including PI3K activity regulation, GTPase activity regulation, and mitochondrial translation (Figure 4E). KEGG pathway analysis of this cluster identified enriched pathways including galactose metabolism, FOXO signaling, and PI3K/AKT signaling (Figure 4F).

### UA-30 ameliorated ALS pathology by enhancing mitochondrial function and mitophagy.

**Figure 5:**
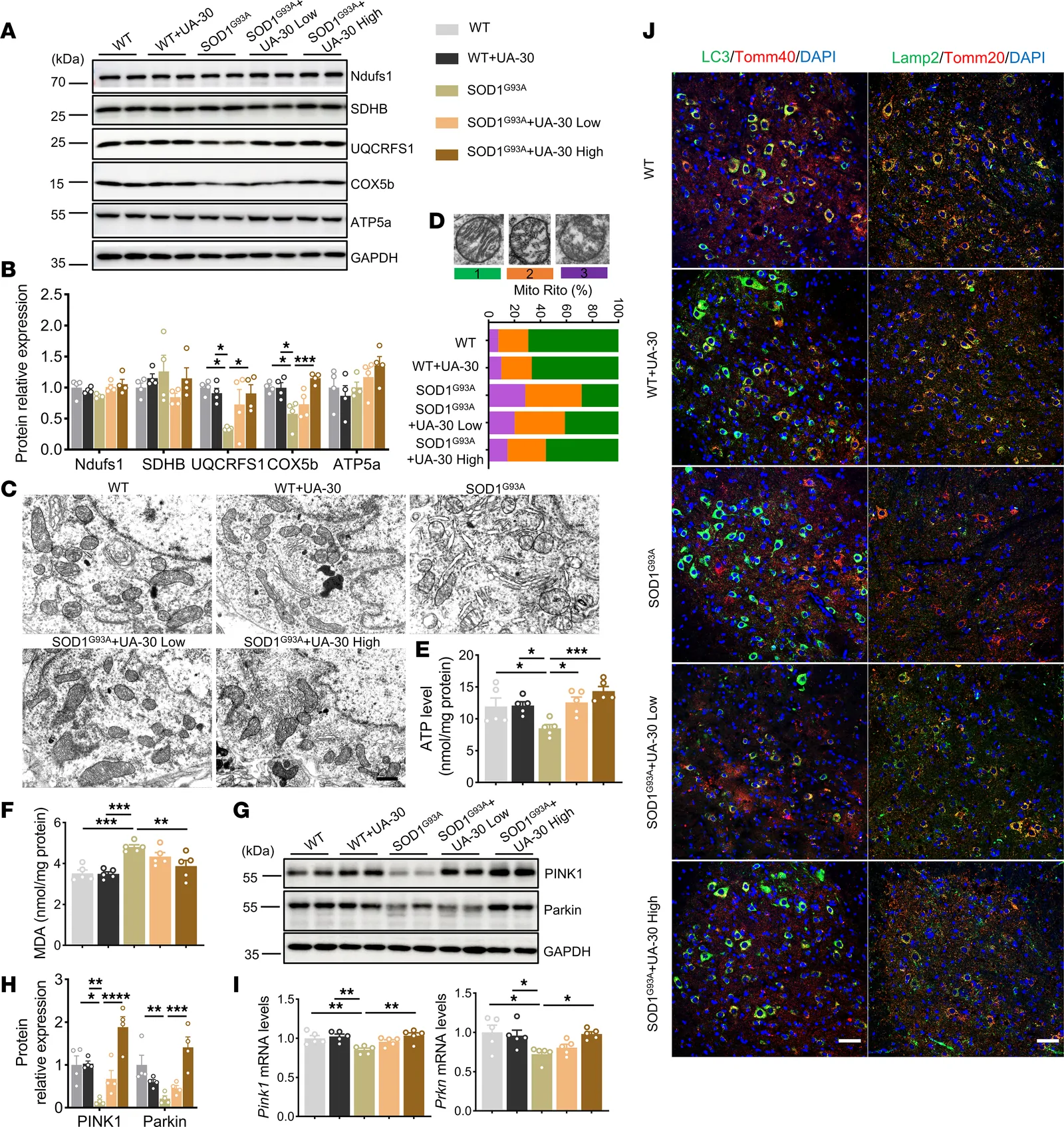
UA-30 treatment improved mitochondrial damage and mitophagy defects in male SOD1^G93A^ mice. (**A** and **B**) Expression and quantification of mitochondrial respiratory complex I (Ndufs1), II (SDHB), III (UQCRFS1), IV (COX5b), and V (ATP5a) in the spinal cord from saline- or UA-30–treated mice. *n* = 4 male per group. (**C** and **D**) Mitochondria morphology in spinal cord neurons of SOD1^G93A^ mice was observed using TEM. Representative mitochondrial images are shown in **C**. Scale bar: 1 μm. Mitochondria were classified into 3 categories based on cristae morphology (green, intact; orange, mild cristae loss; purple, severe cristae loss). The proportion of each mitochondria category was quantified separately (**D**), and data are presented as frequency distributions. At least 150 mitochondria from 3 male mice were analyzed in each group. (**E** and **F**) Steady-state ATP content and lipid peroxidation levels were measured in the spinal cord of SOD1^G93A^ mice exposed to different treatments. *n* = 5 male per group. (**G** and **H**) Western blot (**G**) and subsequent quantitation (**H**) for levels of PINK1 and Parkin in spinal cord after UA-30 treatment in SOD1^G93A^ mice. *n* = 4 male per group. (**I**) RT-qPCR analysis of *Pink1* and *Prkn* mRNA levels in spinal cord of UA-30–treated SOD1^G93A^ mice. *n* = 5 male per group. (**J**) Co-immunofluorescence staining of LC3 (green) and Tomm40 (red) and co-immunofluorescence staining of Lamp2 (green) and Tomm20 (red) in the spinal cord of male mice treated with UA-30. Scale bars: 50 μm. Data are expressed as mean ± SEM. *P < 0.05, **P < 0.01 ***P < 0.001, and ****P < 0.0001. 1-way ANOVA followed by Dunnett’s multiple-comparison test.

Proteomic analysis in this study indicated that UA-30 partially alleviated ALS-related changes by modulating mitochondrial and intracellular homeostasis. To determine the impact of UA-30 on mitochondria, we analyzed mitochondrial oxidative phosphorylation proteins in the spinal cord and medulla oblongata of SOD1^G93A^ mice. UA-30 treatment increased levels of ETC complex III (UQCRFS1) and complex IV (COX5b) in the spinal cord (Figure 5, A and B) and elevated levels of complexes I (Ndufs1), III, and IV in the medulla oblongata (Supplemental Figure 12, A and B). Consistent with these findings, UA-30 improved levels of ETC complexes III and IV in PC12 cells transfected with hSOD1^G93A^ (an ALS cellular model; Supplemental Figure 13, A–C) (22). Mitochondria were categorized into 3 types based on the degree of cristae loss (23). Transmission electron microscopy (TEM) revealed that spinal cord neurons of SOD1^G93A^ mice exhibited rounded, swollen mitochondria with disorganized or dissipated cristae. UA-30 intervention effectively mitigated these mitochondrial morphological defects (Figure 5, C and D). Furthermore, UA-30 reversed the decrease in ATP levels and the increase in malondialdehyde (MDA) levels observed in the spinal cord, medulla oblongata, and gastrocnemius muscle of SOD1^G93A^ mice (Figure 5, E and F, and Supplemental Figure 12, C and D). Consistently, UA-30 increased ATP levels, restored mitochondrial membrane potential (MMP), and reduced ROS and mitochondrial ROS (mitoROS) levels in SOD1^G93A^ cells (Supplemental Figure 13, D–F).

Based on proteomic data suggesting UA-30 involvement in autophagy pathways, we first assessed LC3 and p62 levels in the medulla oblongata. High-dose UA-30 treatment reduced the elevated levels of LC3 (by ~99%) and p62 (by ~60%) in SOD1^G93A^ mice (Supplemental Figure 14, A and B). We therefore next focused on the mitochondrial clearance pathway. In both the spinal cord and medulla oblongata, PINK1 and Parkin levels were markedly reduced in SOD1^G93A^ mice, and this decline was reversed by UA-30 treatment (Figure 5, G and H, and Supplemental Figure 14, A and B). Similarly, UA-30 increased PINK1 and Parkin levels in SOD1^G93A^ cells (by ~100% and ~50%, respectively) (Supplemental Figure 14, C and D). Real-time qPCR analysis further confirmed that UA-30 ameliorated the decrease in *Pink1* and *Prkn* mRNA levels in the spinal cord of SOD1^G93A^ mice (Figure 5I). In the mitochondrial fraction, both PINK1 and Parkin were markedly decreased in SOD1^G93A^ mice compared with WT controls, whereas UA-30 treatment restored their mitochondrial accumulation (Supplemental Figure 15, A and B). In addition, immunofluorescence staining showed that p-Ubiquitin and p-Parkin signals in the ventral horn of the spinal cord were markedly reduced in SOD1^G93A^ mice but restored after UA-30 treatment (Supplemental Figure 15, C and D). Together, these data indicate that UA-30 not only increases PINK1 and Parkin expression, but also promotes mitochondrial recruitment and activation of the PINK1/Parkin mitophagy machinery. Immunofluorescence analysis further demonstrated reduced colocalization of LC3 (autophagosome marker) with Tomm40 (mitochondrial marker) and of Lamp2 (lysosome marker) with Tomm20 (mitochondrial marker) in the ventral horn of the spinal cord of SOD1^G93A^ mice compared with controls. Both colocalization events increased following UA-30 treatment (Figure 5J). UA-30 also promoted an increase in LC3 puncta and improved mitochondrial integrity in SOD1^G93A^-transfected cells (Supplemental Figure 14E). Taken together, these findings indicate that UA-30 promotes PINK1/Parkin-mediated mitophagy-related processes and mitochondrial quality control in the SOD1^G93A^ model.

### UA-30 stabilized FOXO3a protein by inhibiting the RalA/ERK/FOXO3a signaling axis.

**Figure 6:**
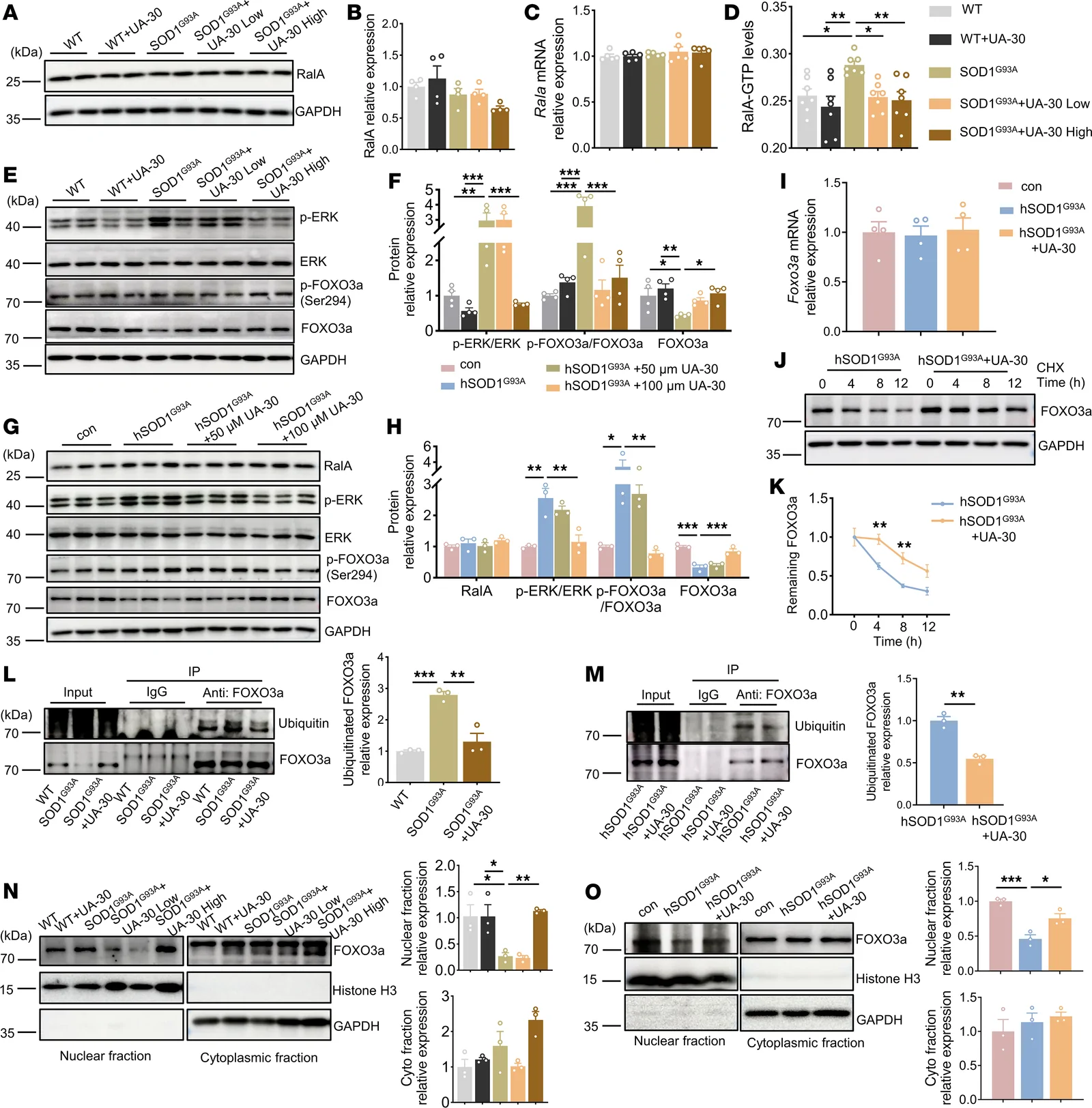
UA-30 treatment regulated the ERK/FOXO3a pathway by inhibiting RalA activity. (**A** and **B**) Western blot and quantification of RalA in spinal cord of different treatment groups. *n* = 4 male per group. (**C**) Relative *Rala* mRNA levels in spinal cord of UA-30 treatment groups. *n* = 5 male per group. (**D**) Analysis of active RalA in spinal cord by G-LISA assay. The level of RalA activity was expressed as RalA-GTP levels. *n* = 7 male per group. (**E** and **F**) Western blot analysis of p-ERK, ERK, p-FOXO3a (Ser294), and FOXO3a expression in spinal cord after UA-30 treatment in SOD1^G93A^ mice. *n* = 4 male per group. (**G** and **H**) Western blot and quantitative analysis of RalA, p-ERK, ERK, p-FOXO3a (Ser294), and FOXO3a in SOD1^G93A^ cells treated with vehicle or UA-30. *n* = 3 per group. (**I**) Relative *Foxo3a* mRNA levels in SOD1^G93A^ cells of UA-30 treatment. *n* = 4 per group. (**J** and **K**) Western blot analysis of FOXO3a expression at 0, 4, 8, and 12 h in SOD1^G93A^ cells after UA-30 or cycloheximide (CHX) treatment. *n* = 3 per group. (**L** and **M**) Immunoprecipitation analysis of ubiquitination levels of FOXO3a in male SOD1^G93A^ mice after 100 mg/kg UA-30 treatment (**L**) or PC12 cells transfected with SOD1^G93A^ after 100 μM UA-30 treatment (**M**). *n* = 3 per group. (**N** and **O**) Immunoblots and quantitation of FOXO3a in nuclear or cytoplasmic fraction in spinal cord after UA-30 treatment in male SOD1^G93A^ mice (**N**) or SOD1^G93A^ cells after 100 μM UA-30 administration. *n* = 3 per group. Data are expressed as mean ± SEM. *P < 0.05, **P < 0.01, and ***P < 0.001. **B**–**D**, **F**, **H**, **I**, **L**, **N**, and **O**, 1-way ANOVA followed by Dunnett’s multiple-comparison test; **K**, 2-way ANOVA followed by Bonferroni’s multiple-comparison test; **M**, unpaired 2-tailed Student’s *t* test.

RalA was identified as a molecular target of UA-30 in complementary target identification experiments. In these studies, thermal proteome profiling, cellular thermal shift assay, and surface plasmon resonance collectively supported the interaction between UA-30 and RalA. Specifically, thermal proteome profiling nominated RalA as a candidate target, cellular thermal shift assay demonstrated a UA-30–dependent thermal shift of RalA in cells, and surface plasmon resonance confirmed direct binding between UA-30 and RalA. Our results indicate that UA-30 did not affect RalA protein levels or *RalA* mRNA levels (Figure 6, A–C). As a Ras-related small GTPase that cycles between a GTP-bound active state and a GDP-bound inactive state, RalA activates downstream effectors when GTP bound (24). Utilizing a small G protein activation assay (G-LISA), we detected elevated RalA-GTP levels in the spinal cord of SOD1^G93A^ mice. UA-30 treatment dose-dependently reduced RalA-GTP levels, with higher doses producing more pronounced inhibitory effects (Figure 6D).

One of the most prominent alterations in the spinal cord proteome of UA-30–treated SOD1^G93A^ mice was associated with the FOXO signaling pathway. FOXO signaling, particularly involving FOXO3a, serves as a critical transcriptional hub regulating mitophagy (25–27). Ral regulates the activation of various transcription factors primarily through the MAPK pathway, wherein ERK acts as a key effector of Ral (28, 29). In both SOD1^G93A^ mice and SOD1^G93A^-transfected cells, UA-30 treatment decreased the levels of phosphorylated ERK (p-ERK/ERK) and phosphorylated FOXO3a (p-FOXO3a/FOXO3a), while concurrently increasing the total FOXO3a protein expression level (Figure 6, E–H).

To elucidate the mechanism by which UA-30 increases FOXO3a protein expression, protein degradation assays were performed in UA-30–treated SOD1^G93A^ cells. UA-30 treatment did not detectably alter *Foxo3a* mRNA levels (Figure 6I). Following inhibition of protein synthesis with cycloheximide, UA-30 suppressed FOXO3a degradation, resulting in higher FOXO3a expression than in untreated SOD1^G93A^ cells (Figure 6, J and K). Previous studies have reported that ERK phosphorylates FOXO3a at Ser294, thereby regulating its degradation via the ubiquitin-proteasome pathway and preventing its nuclear translocation (30, 31). Co-IP assays revealed that UA-30 administration inhibited FOXO3a ubiquitination compared with the vehicle-treated SOD1^G93A^ group, by approximately 50% in mice (*P* < 0.01) and 45% in cells (*P* < 0.01) (Figure 6, L and M). These findings confirm that UA-30 inhibits FOXO3a protein degradation through the ubiquitin-proteasome pathway. Furthermore, both in vivo and in vitro experiments revealed that UA-30 promoted an increase in FOXO3a levels within the nuclear fraction (by ~110% and ~55%, respectively, compared with SOD1^G93A^), while not affecting FOXO3a levels in the cytoplasmic fraction (Figure 6, N and O).

### Overexpression of active RalA reversed UA-30’s effects on mitophagy via the RalA/ERK/FOXO3a pathway.

**Figure 7:**
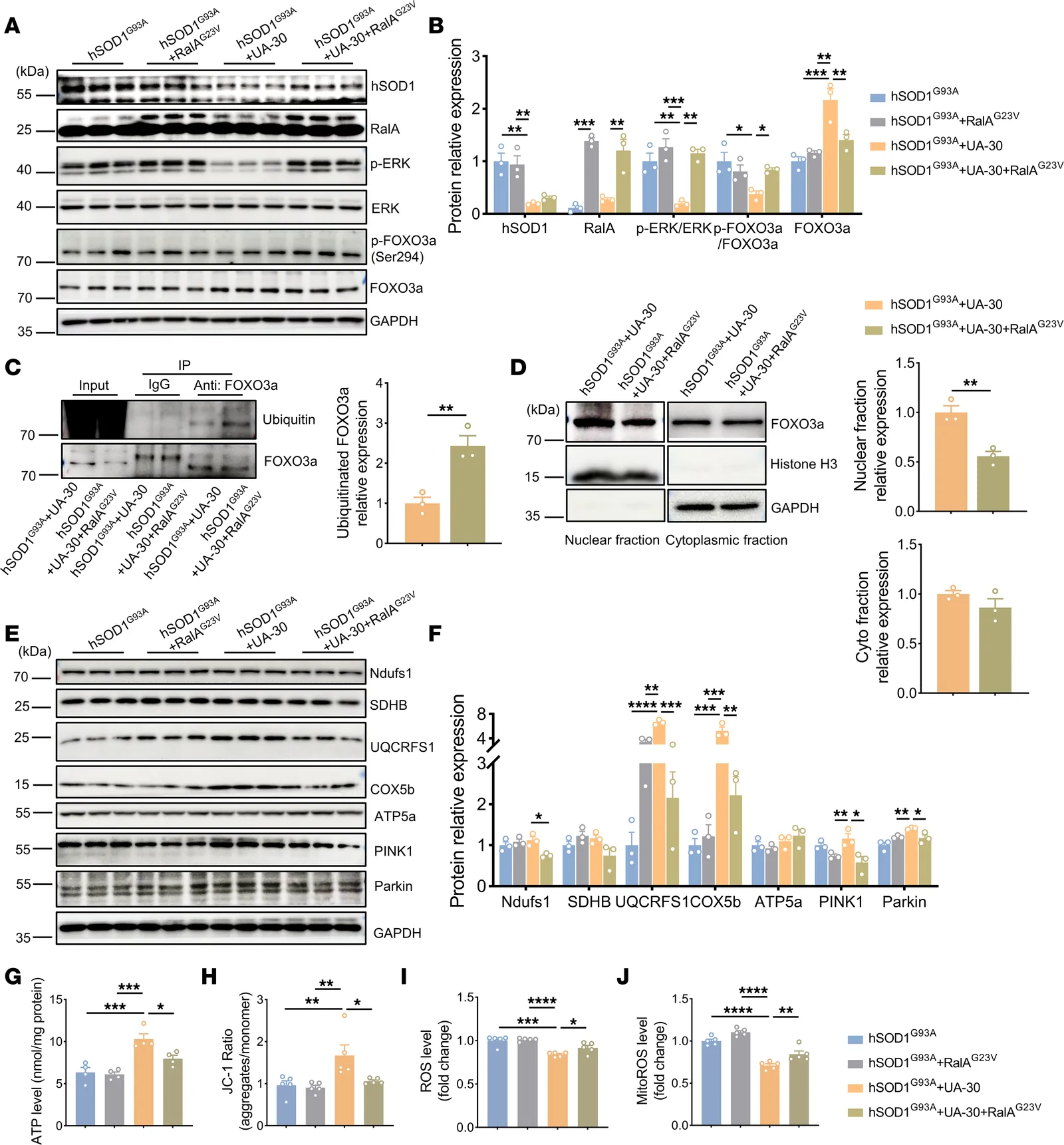
Overexpression of active RalA abolished the impact of UA-30 on mitophagy and the ERK/FOXO3a pathway. (**A** and **B**) After RalA^G23V^ overexpression, immunoblot analysis and quantitation of hSOD1, RalA, p-ERK, ERK, p-FOXO3a (Ser294), and FOXO3a in SOD1^G93A^ cells treated with 100 μM UA-30. *n* = 3 per group. (**C**) The immunoprecipitation analysis of ubiquitination levels of FOXO3a in PC12 cells transfected with SOD1^G93A^ after 100 μM UA-30 or RalA^G23V^ overexpression treatment. *n* = 3 per group. (**D**) Western blot analysis of FOXO3a expression in nuclear or cytoplasmic fraction of SOD1^G93A^ cells after 100 μM UA-30 and RalA^G23V^ overexpression treatment. *n* = 3 per group. (**E** and **F**) Western blot analysis of the levels of mitochondrial respiratory complex, PINK1, and Parkin in SOD1^G93A^ cells overexpressing RalA^G23V^. *n* = 3 per group. (**G**–**J**) Assessment of ATP level (**G**), MMP level (JC-1 staining red/green fluorescence intensity) (**H**), ROS production (**I**), and mitoROS level (**J**) in SOD1^G93A^ cells treated with 100 μM UA-30 and overexpressing RalA^G23V^. *n* = 4–5 per group. Data are expressed as mean ± SEM. *P < 0.05, **P < 0.01 ***P < 0.001, and ****P < 0.0001. **B** and **F**–**J**, 1-way ANOVA followed by Dunnett’s multiple-comparison test; **C** and **D**, unpaired 2-tailed Student’s *t* test.

To further investigate whether UA-30 improves mitophagy and mitochondrial function through the RalA/ERK/FOXO3a signaling pathway, we overexpressed constitutively active RalA (RalA^G23V^) (32) in UA-30–treated SOD1^G93A^ cells. To determine whether RalA^G23V^ itself causes mitochondrial impairment under unstressed conditions, we also expressed RalA^G23V^ in WT PC12 cells. RalA^G23V^ expression did not affect cell viability, ATP levels, MDA levels, total SOD activity, or mitoROS signals in WT PC12 cells (Supplemental Figure 16, A–E). Overexpression of active RalA reversed the UA-30–induced suppression of the ERK/FOXO3a signaling pathway. Specifically, it increased the p-ERK/ERK and p-FOXO3a/FOXO3a ratios while decreasing total FOXO3a protein levels (Figure 7, A and B). Furthermore, overexpression of active RalA enhanced the ubiquitination of FOXO3a and counteracted the UA-30–mediated increase in FOXO3a nuclear translocation in cells (Figure 7, C and D).

Subsequently, overexpression of active RalA reduced the UA-30–induced protein levels of ETC complexes I, III, and IV, as well as PINK1 and Parkin, in SOD1^G93A^ cells (Figure 7, E and F). Furthermore, active RalA overexpression in SOD1^G93A^ cells attenuated the ameliorative effects of UA-30 on mitochondrial dysfunction, as evidenced by decreased ATP levels and MMP and increased levels of total cellular ROS and mitoROS (Figure 7, G–J).

Collectively, these results demonstrate that UA-30 ameliorates mitochondrial dysfunction in the SOD1^G93A^ model by enhancing mitophagy, mediated through the targeted suppression of the RalA/ERK/FOXO3a signaling pathway.

### Mdivi-1 abrogated the UA-30–induced improvement in mitochondrial function and neuroprotection in SOD1^G93A^ mice.

**Figure 8:**
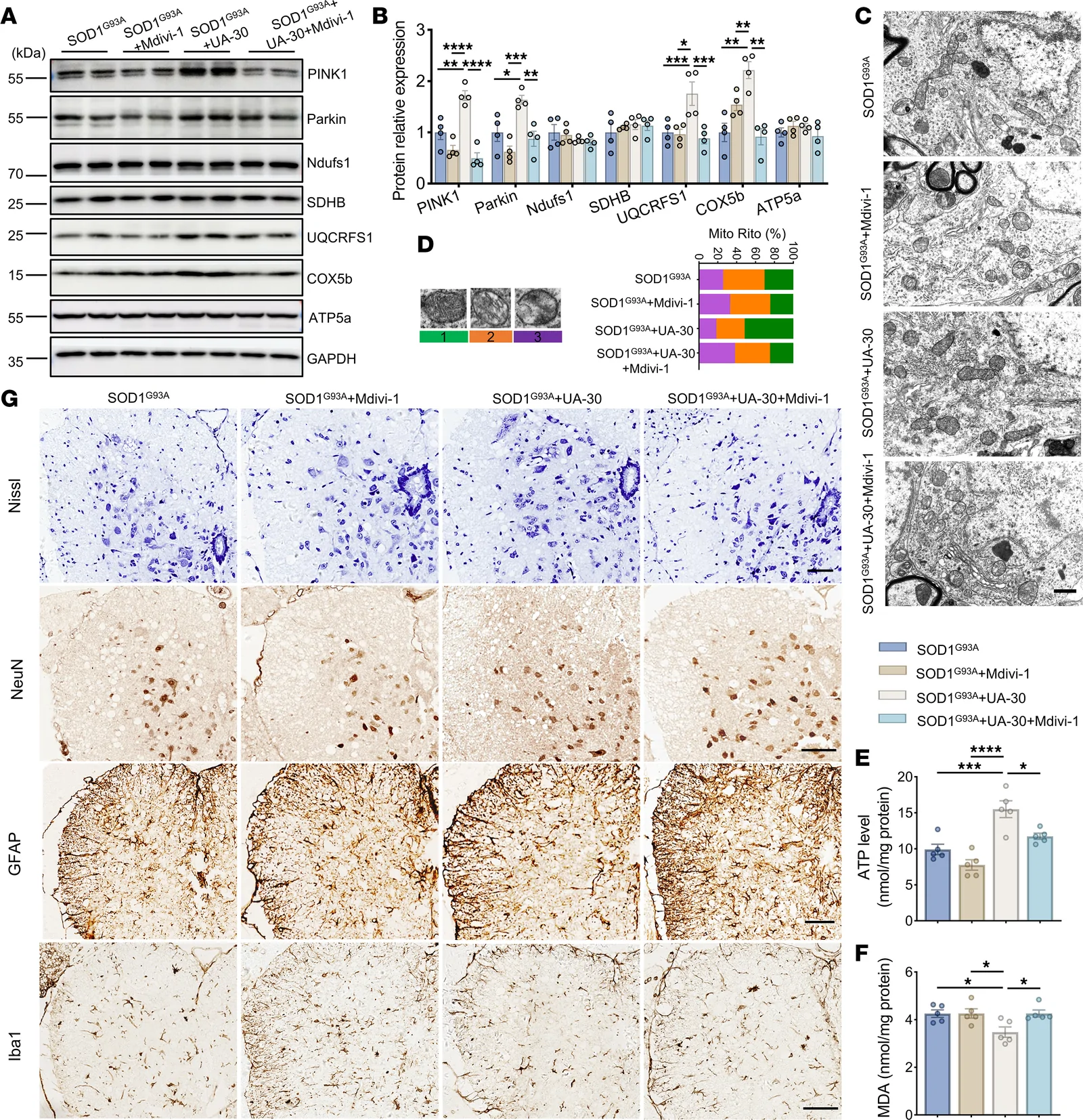
Mdivi-1 eliminated the beneficial effects of UA-30 on motor neuron loss and neuroinflammation. (**A** and **B**) Western blot analysis and quantification of PINK1, Parkin, and mitochondrial respiratory complex protein levels in spinal cord of SOD1^G93A^ mice treated with UA-30 or Mdivi-1. *n* = 4 male per group. (**C** and **D**) Representative mitochondrial images (**C**) were observed in the spinal cord neurons of male SOD1^G93A^ mice from different treatment groups using TEM. Scale bar: 1 μm. Mitochondria were classified into 3 categories based on cristae morphology (green, intact; orange, mild cristae loss; purple, severe cristae loss). The proportion of each mitochondria category was quantified separately (**D**), and data are presented as frequency distributions. At least 150 mitochondria from 3 mice were analyzed in each group. (**E** and **F**) Measurement of ATP level and lipid peroxidation level in spinal cord of SOD1^G93A^ mice after UA-30 or Mdivi-1 treatment. *n* = 5 male per group. (**G**) Representative images of Nissl staining and neurons (NeuN), astrocytes (GFAP), and microglia (Iba1) immunohistochemical staining in spinal cord after UA-30 and Mdivi-1 treatment in male SOD1^G93A^ mice. Scale bars: 100 μm (Nissl), 200 μm (NeuN), 200 μm (GFAP), 200 μm (Iba1). Data are expressed as mean ± SEM. *P < 0.05, **P < 0.01 ***P < 0.001, and ****P < 0.0001. 1-way ANOVA followed by Dunnett’s multiple-comparison test.

To corroborate the involvement of mitophagy in UA-30’s therapeutic mechanism against ALS, Mdivi-1 was used to pharmacologically interfere with mitophagy-related signaling (33, 34). SOD1^G93A^ mice received combined treatment with Mdivi-1 and UA-30. Spinal cord tissues from Mdivi-1–treated mice showed reduced levels of PINK1 and Parkin proteins (Figure 8, A and B), indicating that Mdivi-1 interfered with mitophagy-related signaling.

Notably, the expression of mitochondrial complex III (UQCRFS1) and complex IV (COX5b) in the spinal cord of UA-30–treated SOD1^G93A^ mice was decreased upon Mdivi-1 treatment (Figure 8, A and B). TEM analysis revealed swollen, vacuolar mitochondria with an apparent loss of cristae in Mdivi-1–treated groups (Figure 8, C and D). Critically, the beneficial effects of UA-30 on increasing ATP levels and reducing MDA levels were completely abolished in the presence of Mdivi-1 (Figure 8, E and F).

**Figure 9:**
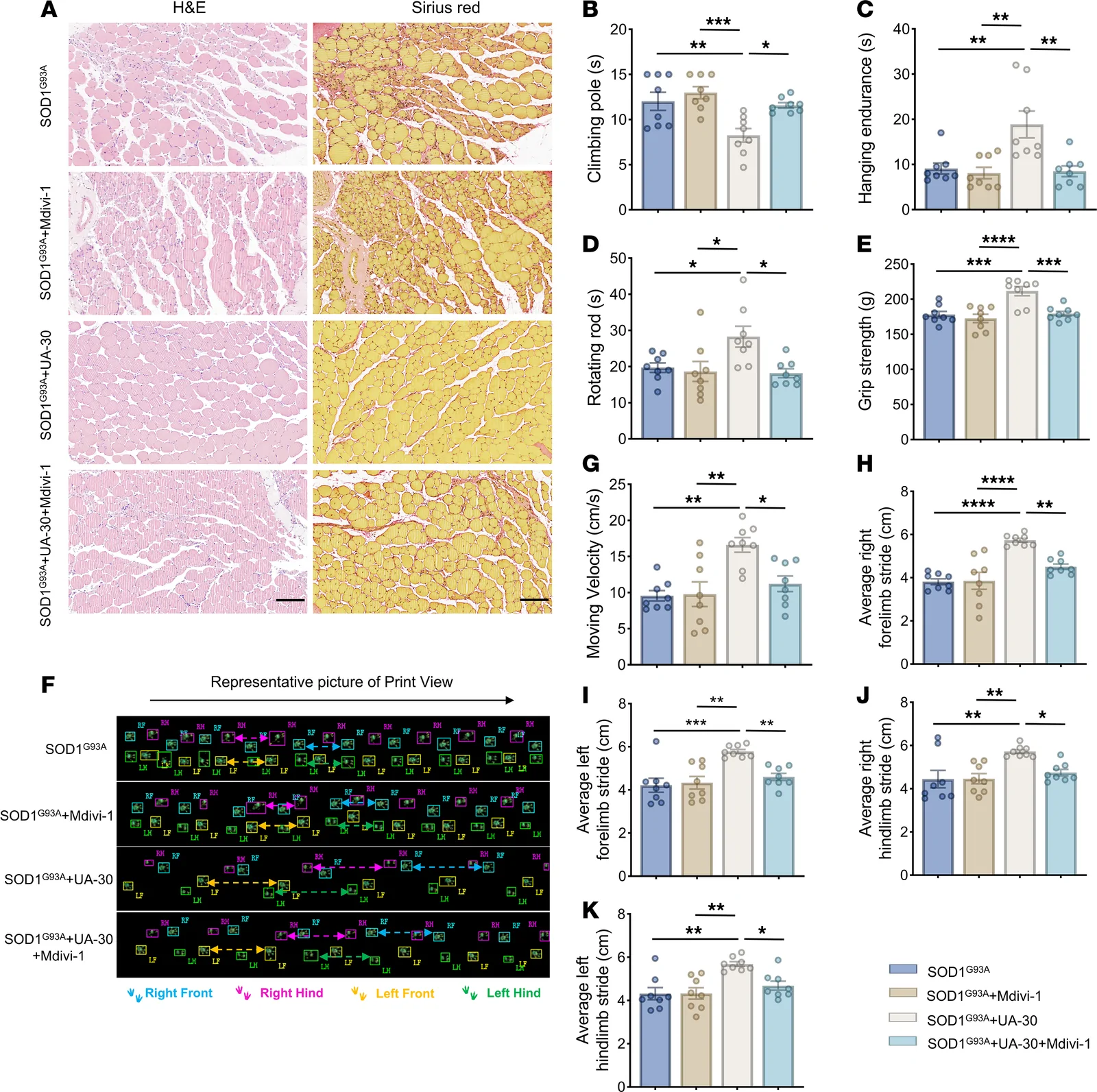
Mdivi-1 attenuated UA-30–induced improvement in motor function. (**A**) Representative histological H&E and Sirius red staining images of the gastrocnemius muscles of male SOD1^G93A^ mice exposed to different treatments. Scale bars: 100 μm (H&E), 100 μm (Sirius red). (**B**–**E**) Motor performance of SOD1^G93A^ mice treated with UA-30 or Mdivi-1 measured by climbing time on pole tests (**B**), hanging endurance time (**C**), retention time on rotarod (**D**), and grip strength (**E**). (**F**–**K**) Representative images of gait tracking via gait analysis (**F**) and quantified stride speed, average stride of left hind limbs and forelimbs or right hind limbs and forelimbs (**G**–**K**). *n* = 8 male per group. Data are expressed as mean ± SEM. *P < 0.05, **P < 0.01, ***P < 0.001, and ****P < 0.0001. 1-way ANOVA followed by Tukey’s multiple-comparison test.

Mdivi-1 eliminated the protective effect of UA-30 against motor neuron loss, as evidenced by reduced neuronal counts using both Nissl staining and NeuN immunohistochemistry (Figure 8G and Supplemental Figure 17, A and B). Furthermore, compared with SOD1^G93A^ mice treated with UA-30 alone, the group receiving combined treatment of UA-30 and Mdivi-1 increased the numbers of astrocytes and microglia (Figure 8G and Supplemental Figure 17, C and D). Additionally, UA-30 treatment reduced muscle inflammation and fibrosis in SOD1^G93A^ mice, which was also counteracted by Mdivi-1 (Figure 9A and Supplemental Figure 17, E and F).

### Mdivi-1 abolished UA-30–induced improvement of motor deficit in SOD1^G93A^ mice.

Behavioral assessments showed that UA-30 reduced climbing time, increased fall latency in the wire hang and rotarod tests, and enhanced grip strength. However, these improvements were completely abolished by coadministration of mdivi-1 (Figure 9, B–E). Similar outcomes were observed in gait analysis (Figure 9, F–K, and Supplemental Video 3). Overall, these findings indicate that administration of mdivi-1 blocked improvement of motor deficit in SOD1^G93A^ mice induced by UA-30.

## Discussion

**Figure 10:**
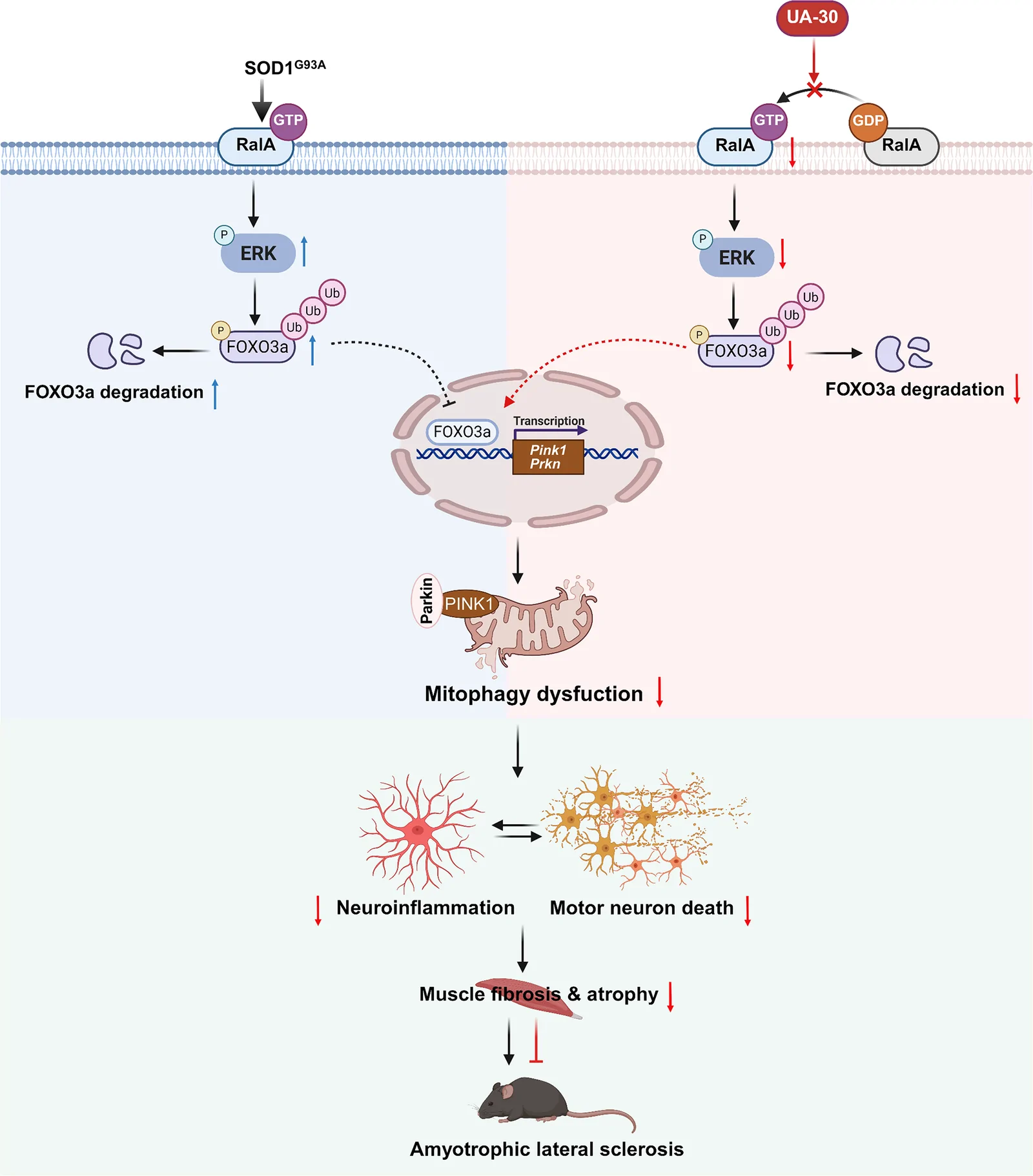
Proposed working model of UA-30 in SOD1^G93A^ mice. In the pathogenesis of ALS, SOD1^G93A^ mutation induces RalA activation, and aberrant activation of RalA triggers a downstream RalA/ERK signaling cascade, which promotes ubiquitin-proteasome–mediated degradation of the key transcription factor FOXO3a. Reduced ubiquitin-proteasome diminishes its nuclear translocation, leading to the downregulation of mitophagy-related genes and consequent impairment of mitophagy. Accumulation of damaged mitochondria contributes to neuroinflammation and ultimately drives motor neuron death, resulting in muscle fibrosis and atrophy. UA-30 exerts its therapeutic effects by directly targeting and inhibiting RalA activation. Suppression of the ERK signaling stabilizes FOXO3a protein levels. Restoration of FOXO3a function enhances mitophagy, clears dysfunctional mitochondria, alleviates neuroinflammation, and prevents motor neuron death, ultimately halting ALS progression. Created with BioRender.

ALS is a lethal neurodegenerative disorder characterized by the progressive degeneration of motor neurons in the brain and spinal cord (1, 2). Despite extensive research, clinically effective pharmacological treatments for ALS remain unavailable. In this study, we evaluated the small-molecule compound UA-30, which has been shown to be safe and exhibits a favorable pharmacokinetic profile. UA-30 achieved CNS exposure after oral administration, ameliorated motor dysfunction, and extended both the median survival and overall lifespan in SOD1^G93A^ mice. UA-30 treatment attenuated key ALS-associated pathological features. Specifically, it mitigated the loss of spinal motor neurons, decreased microgliosis and astrogliosis, and reduced the degree of gastrocnemius muscle fibrosis. Mechanistically, as an inhibitor of RalA GTPase, UA-30 suppressed RalA activation and modulated the ERK/FOXO3a signaling pathway. UA-30 inhibited FOXO3a degradation via the ubiquitin-proteasome pathway, enhanced FOXO3a stability, and supported transcription of mitophagy-related genes, including *Pink1* and *Prkn*. Consistent with this upstream transcriptional support, UA-30 enhanced PINK1/Parkin-dependent mitophagy and improved mitochondrial dysfunction (Figure 10). Collectively, these results support UA-30 as a promising therapeutic candidate for ALS and provide a foundation for future preclinical evaluation.

RalA, a member of the Ras superfamily of small GTPases, functions as a molecular switch cycling between an active GTP-bound state and an inactive GDP-bound state (24). RalA translocates to mitochondria following depolarization, suggesting its potential involvement in mitophagy (35). Furthermore, aberrant RalA activation promotes excessive mitochondrial fission in adipocytes of high-fat diet–induced obese mice, impairing mitochondrial function and metabolic capacity (32). As a downstream effector of Ras, RalA regulates multiple signaling pathways, including the ERK/MAPK cascade (29, 36, 37). In our study, activation of RalA increased ERK phosphorylation at Thr202/Tyr204, supporting the involvement of the RalA/ERK axis under our experimental conditions. Activated ERK has been reported to phosphorylate FOXO3a at Ser294, Ser344, and Ser425, thereby enhancing its interaction with the E3 ubiquitin ligase MDM2 and promoting ubiquitin-proteasome–dependent degradation (30, 31). Thus, phosphorylation of FOXO3a at Ser294 is primarily associated with FOXO3a ubiquitination and reduced protein stability. By contrast, FOXO3a nuclear exclusion and cytoplasmic retention are more classically regulated by phosphorylation at sites such as Thr32, Ser253, and Ser315, which promotes 14-3-3 binding and retention of FOXO3a in the cytoplasm (38–40). Our results showed that UA-30 increased total FOXO3a protein levels and promoted FOXO3a nuclear accumulation, whereas overexpression of constitutively active RalA abolished the regulatory effects of UA-30 on the RalA/ERK/FOXO3a axis and impaired FOXO3a nuclear import. These findings suggest that UA-30 suppresses RalA/ERK signaling, reduces inhibitory FOXO3a phosphorylation and ubiquitination, and restores FOXO3a nuclear localization.

FOXO3a is a well-conserved transcription factor critical for stress resistance, longevity, and cellular homeostasis (41). Activation of FOXO3a in the *C*. *elegans* model has been shown to induce mitophagy, markedly ameliorating muscle aging and mitochondrial dysfunction (42). Previous studies further support a transcriptional role of FOXO3a in mitophagy regulation, as the genes encoding PINK1 and Parkin can be regulated by FOXO3a, and disruption of the FOXO3a/PINK1/Parkin axis impairs mitophagy restoration (27, 43). Consistent with this framework, our results support FOXO3a-dependent transcriptional regulation as an upstream component of UA-30–enhanced mitophagy. UA-30 increased FOXO3a nuclear localization and was associated with transcriptional upregulation of *Pink1* and *Prkn*, together with enhanced PINK1/Parkin-mediated mitophagy. It should be noted that UA-30 leads to a drastic increase in mitophagy protein levels but has only mild effects on their transcripts. This suggests a posttranscriptional regulatory mechanism, independent of FOXO3a, which remains to be investigated in future studies. Overexpression of active RalA counteracted the UA-30–induced increase in FOXO3a nuclear localization and the associated enhancement of mitophagy. In addition, in the SOD1^G93A^ cellular model where mitochondrial dysfunction is already pronounced, constitutively active RalA did not necessarily cause a further measurable decline in these mitochondrial parameters but was sufficient to abolish the mitochondrial improvements conferred by UA-30. These findings suggest that the pathological consequences of RalA activation are context dependent and become more evident in the setting of SOD1^G93A^-associated proteotoxic stress. Consistent with this interpretation, the protective effects of UA-30 are most apparent under disease-associated stress conditions. A similar pattern was also observed in the pharmacological intervention study involving Mdivi-1. In the SOD1^G93A^ model, Mdivi-1 abolished the protective effects of UA-30 but did not further worsen baseline outcomes under the treatment window used here.

Because Mdivi-1 primarily inhibits Drp1-dependent mitochondrial fission, its effect on mitophagy should be regarded as indirect rather than direct. Nevertheless, inhibition of mitochondrial fission can still attenuate mitophagy-related signaling in the PINK1/Parkin pathway (44). In this context, the reduction in Parkin observed after Mdivi-1 treatment is more plausibly interpreted as a downstream consequence of attenuated mitophagy-related signaling, rather than direct suppression of transcription of the gene encoding Parkin or Parkin protein synthesis. Although previous studies have suggested that Mdivi-1 alone does not markedly alter behavioral performance in WT or control animals (45, 46), the effects of Mdivi-1 alone on motor function in WT mice and on survival in SOD1^G93A^ mice were not systematically evaluated this study and remain as limitations to be addressed in future work.

Deficiencies in mitochondrial respiratory chain complex IV are widely observed in patients with sporadic ALS (47, 48). Furthermore, swollen and vacuolated mitochondria are markedly increased in spinal motor neurons of ALS patients (49, 50). Impairment of mitophagy leads to the accumulation of dysfunctional mitochondria, disrupting mitochondrial regulatory functions. Our findings demonstrate that UA-30 treatment reversed the decrease in ATP levels and the increase in MDA levels observed in the spinal cord, medulla oblongata, and gastrocnemius muscle of ALS mice. Additionally, UA-30 treatment increased the expression levels of mitochondrial complex III (UQCRFS1) and IV (COX5b) in the spinal cord and medulla oblongata. UA-30 also ameliorated mitochondrial damage in spinal cord neurons of ALS mice. Mdivi-1 treatment suppressed the expression of PINK1 and Parkin. This inhibition of mitophagy initiation blocked the beneficial effects of UA-30 on mitochondrial function. These results suggest that UA-30 improves mitochondrial dysfunction in ALS mice by enhancing mitophagy.

Mitochondrial dysfunction drives ALS progression through interrelated mechanisms, including bioenergetic deficits, oxidative stress, and neuroinflammation (51–53). This pathogenic framework underlies the key pathological features observed in ALS mice, including spinal motor neuron loss, microglial and astrocytic activation, muscle denervation and atrophy, as well as muscle fibrosis. Consistent with mitigating core pathologies, UA-30 treatment increased spinal motor neuron survival, reduced microgliosis and astrogliosis, and inhibited muscle fibrosis. These improvements collectively contributed to the enhanced motor performance observed in UA-30–treated ALS mice. While our study demonstrates that UA-30 reduces microgliosis in SOD1^G93A^ mice, the functional phenotype of microglia is also likely to be important. In ALS, microglia undergo dynamic and stage-dependent changes, and their activation states may range from relatively protective to proinflammatory phenotypes rather than fitting strictly into a simple M1/M2 dichotomy (54–56). Therefore, the therapeutic effects of UA-30 may involve not only reducing overall microglial abundance, but also modulating microglial phenotype and inflammatory output. This possibility needs to be addressed in future studies using established polarization-related markers and pathways.

Additional assessment of NMJs showed that UA-30 only partially improved NMJ innervation in the gastrocnemius muscle of SOD1^G93A^ mice. This finding may explain why UA-30 produced substantial improvements in muscle pathology and motor neuron survival, yet only a modest survival benefit. Given that treatment was initiated at 13 weeks of age, when denervation is already underway in this model, the therapeutic window for fully preserving distal neuromuscular connectivity may have been limited.

In an additional equimolar comparison, UA-30 improved several motor performance readouts more than UA under matched conditions, and together with its improved oral CNS exposure, these data support UA-30 as a more developable compound based on the UA scaffold for further evaluation. It should also be noted that behavioral outcomes did not exhibit a strictly dose-dependent pattern, with low and high doses often yielding comparable effects despite numerical separation in some measures. This pattern may reflect an in vivo plateau effect, whereby the lower dose is already sufficient to produce near-maximal efficacy for certain functional endpoints, and further dose escalation therefore yields only limited additional benefit. Furthermore, the invasive nature of delivery routes, intravenous for edaravone and intrathecal for tofersen, can substantially compromise the quality of life for ALS patients, further limiting their utility. In contrast, UA-30’s ability to achieve neuroprotection through oral administration makes it more accessible and less invasive. Here, UA-30 showed a trend toward improved efficacy compared with the first-tier drug riluzole, specifically in terms of improved motor performance in pole-climbing, hanging, and rotarod tests, prolonged median survival in ALS mice, and inhibition of muscle fibrosis. Given that UA-30 and riluzole act through distinct mechanisms, it is also important for future studies to determine whether UA-30 combined with riluzole can provide an additive or synergistic benefit.

Based on its oral convenience, favorable safety profile (LD_50_ = 1,992.5 mg/kg, NOAEL > 400 mg/kg), and the preclinical efficacy observed here, UA-30 represents a promising therapeutic candidate for ALS. As ALS diagnosis shifts toward earlier recognition (57), earlier intervention, such as presymptomatic administration of UA-30, may provide greater therapeutic benefit, further delay disease progression, and enhance neuroprotection. These preclinical findings provide a solid foundation for continued evaluation of UA-30.

## Methods

### Sex as a biological variable.

Both male and female SOD1^G93A^ mice were included in the principal behavioral and survival studies. Male and female cohorts were analyzed separately for these endpoints, and sex-specific data are shown in the main figures and supplemental materials. Gastrocnemius muscle histopathology and subsequent mechanistic experiments were performed in male mice to maintain consistency with the primary tissue analyses and to minimize variability associated with sex-dependent differences in disease progression in the SOD1^G93A^ model. Sex and sample size are specified in the corresponding figure legends.

### Animal treatment.

UA-30 was synthesized and provided by the State Key Laboratory of Biotherapy, West China Hospital, Sichuan University. Compound purity (>99.6%) was determined by HPLC, and chemical identity was confirmed by NMR and mass spectrometry (MS). The chemical structure of UA-30 is shown in Supplemental Figure 1. For in vivo experiments, UA-30 was dissolved in sterile normal saline (0.9% NaCl) and administered at the indicated doses.

Transgenic ALS model mice expressing human *SOD1* with the G93A mutation were obtained from The Jackson Laboratory [stock 004435, B6Cg-Tg (SOD1*G93A)1Gur/J]. The mice were housed under a 12 h light/dark cycle with stable temperature (20°C ± 2°C) and humidity (55% ± 5%).

SOD1^G93A^ mice were randomly divided into 6 experimental treatment groups: (a) saline control (SOD1^G93A^), (b) UA-30 low (25 mg/kg), (c) UA-30 medium (50 mg/kg), (d) UA-30 high (100 mg/kg), (e) riluzole (10 mg/kg; Sigma-Aldrich) (58), and (f) UA (10 mg/kg; StanYouth, Bonerge Lifescience). Nontransgenic mice of gender-matched littermates were randomly divided into 2 groups: (a) saline control (WT) and (b) UA-30 (100 mg/kg; WT+UA-30). All mice were 13 weeks old at the start of the experiment. Treatments were administered once daily via oral gavage for 6 weeks, while negative-control groups received equivalent volumes of saline per os. The detailed treatment regimen is shown in Figure 1A. Behavioral assessments were performed at baseline prior to treatment initiation and every 2 weeks during the 6-week treatment period. Body weight was recorded twice weekly. After completion of the final behavioral assessment at the experimental endpoint, mice were euthanized and serum and tissue samples were collected for subsequent pathological, biochemical, and molecular analyses.

### Survival analysis.

Male and female SOD1^G93A^ mice were assigned to saline, UA-30 (100 mg/kg), or riluzole (10 mg/kg) groups. Daily oral gavage was initiated at 13 weeks of age and continued until natural death or humane euthanasia according to predefined criteria. Detailed survival study procedures and euthanasia criteria are provided in Supplemental Methods.

### Behavioral tests.

Behavioral assessments were conducted at baseline and every 2 weeks during the 6-week treatment period and included gait analysis, pole-climbing, wire-hanging, rotarod, and grip-strength tests. Detailed procedures are described in Supplemental Methods.

### Cell lines.

PC12 cells were obtained from the Cell Bank of Type Culture Collection of the Chinese Academy of Sciences and cultured in RPMI-1640 supplemented with 10% FBS at 37°C under 5% CO_2_. Cells were transiently transfected with plasmids encoding human SOD1^G93A^ or constitutively active RalA^G23V^, followed by treatment with UA-30 or vehicle. Detailed plasmids and transfection procedures are described in Supplemental Methods.

### Protein, transcript, and mitochondrial analyses.

Protein expression was assessed by Western blotting and immunoprecipitation, and transcript levels were measured by RT-qPCR. Nuclear/cytoplasmic or mitochondrial fractionation was performed where indicated. Mitochondrial function was assessed in tissues or cells by measuring ATP, MDA, total SOD activity, ROS, mitoROS, and MMP. TEM was used to examine mitochondrial ultrastructure in spinal cord neurons. Detailed protocols, reagents, antibodies, primers, and analytical conditions are provided in Supplemental Methods and Supplemental Tables 2 and 3.

### Proteomics.

TMT-based quantitative proteomics was performed on spinal cord (L4-L5) and medulla oblongata tissues from 4 biological replicates per group based on established principles (59). DE proteins were identified using Student’s *t* test (*P* < 0.05), followed by unsupervised hierarchical clustering and Gene Ontology/KEGG enrichment analyses. Full details of sample preparation, liquid chromatography–tandem MS (LC-MS/MS) acquisition, and bioinformatic analysis are provided in Supplemental Methods.

### RalA activation assay.

The spinal cord (L4-L5) tissues were lysed with lysis buffer including protease inhibitors. RalA-GTP activity was measured following the protocol of the RalA G-LISA GTPase Activation Assay Kit (catalog BK129, Cytoskeleton).

### LC-MS/MS analysis.

To assess tissue exposure, spinal cord and brain samples were collected at defined time points after a single oral dose of 100 mg/kg UA-30 and analyzed by LC-MS/MS. Detailed extraction, chromatography, and MS parameters are described in Supplemental Methods.

### Statistics.

The data are presented as mean ± SEM and were analyzed using GraphPad Prism 9.0 statistical software (GraphPad Software). An unpaired 2-tailed Student’s *t* test was used to compare 2 groups. For comparisons involving more than 2 groups, statistical significance was generally assessed by 1-way ANOVA followed by Dunnett’s multiple-comparison test, except that behavioral data were analyzed using 1-way ANOVA with Tukey’s multiple-comparison test. Body weight outcomes were analyzed using 2-way ANOVA with Bonferroni’s multiple-comparison test. Survival rates were statistically evaluated using the log-rank (Mantel-Cox) test. A *P* value less than 0.05 was considered statistically significant.

### Study approval.

All animal care and experimental procedures were conducted in accordance with institutional guidelines from the Experimental Animal Center of Shenzhen Center for Disease Control and Prevention, with the study protocol approved by the Institutional Animal Care and Use Committee (Animal Ethics Approval 2024022).

### Data availability.

Values for all data points in graphs are reported in the Supporting Data Values file. The mass spectrometry proteomics data have been deposited in the ProteomeXchange Consortium via the iProX partner repository with the dataset identifier PXD076557.

## Author contributions

XY, GL, and YX assumed a pivotal role in both conceptualizing the project and designing the experiments. RZ, CY, X Liu, and YX designed and synthesized the small-molecule compound UA-30. BZ, YH, and TS designed and carried out the majority of the experiments. X Li, XZ, RL, XH, QH, and ZZ assisted with in vivo and in vitro experiments. BZ, GL, and XY analyzed data and wrote the manuscript. The final manuscript was reviewed and approved by all authors.

## Conflict of interest

The authors have declared that no conflict of interest exists.

## Funding support

- Natural Science Foundation of China (82071221 and 82271474 to GL; 82574383 to ZZ).

## Supplementary Material

Supplemental data

Unedited blot and gel images

Supplemental video 1

Supplemental video 2

Supplemental video 3

Supporting data values

## Version 1 09/15/2026

Electronic publication
