# Supplementary material for: UA-30 ameliorates motor deficits through RalA-mediated mitophagy in ALS mice
Source: J Clin Invest. 2026 Sep 15;136(18):e202787. doi: 10.1172/JCI202787 (PMC13574146; doi:10.1172/JCI202787)
Supplement: Supplementary file 1 [file jci-136-202787-s089.pdf]

Supplemental Materials for

UA-30 ameliorates motor deficits through RalA-mediated mitophagy in ALS mice

Bingge Zhang[1, *], Ye He[1, 2, *], Ting Su[2, *], Xiaomei Li[2], Xiufen Zhang[2], Ruijuan Liu[1, 2], Xiao Han[2], Ruiming Zhang[3], Chao Yang[3], Xinlei Liu[3], Qinghua Hou[4], Zaijun Zhang[5], Yongmei Xie[3, #], Gongping Liu[1, 6, #], Xifei Yang[2, #]

[1]Department of Pathophysiology, School of Basic Medicine and the Collaborative Innovation Center for Brain Science, Key Laboratory of Ministry of Education of China and Hubei Province for Neurological Disorders, Tongji Medical College, Huazhong University of Science and Technology, Wuhan, China.

[2]Shenzhen Key Laboratory of Modern Toxicology, Shenzhen Medical Key Discipline of Health Toxicology (2020-2024), Shenzhen Center for Disease Control and Prevention.

[3]State Key Laboratory of Biotherapy, West China Hospital, Sichuan University, and Collaborative Innovation Center for Biotherapy, Sichuan University, Chengdu, China.

[4]Department of Neurology, Clinical Neuroscience Center, the Seventh Affiliated Hospital, Sun Yat-sen University, Shenzhen, China.

[5]State Key Laboratory of Bioactive Molecules and Druggability Assessment; Guangdong Basic Research Center of Excellence for Natural Bioactive Molecules and Discovery of Innovative Drugs; Institute of New Drug Research, College of Pharmacy, Jinan University, Guangzhou, China.

[6]Co-innovation Center of Neuroregeneration, Nantong University, Nantong, Jiangsu, China.

*These authors contributed equally to this work.

#Corresponding authors:

Xifei Yang, Key Laboratory of Modern Toxicology of Shenzhen, Shenzhen Center for Disease Control and Prevention, No. 8, Longyuan Road, Nanshan District, Shenzhen 518055, China. Email: xifeiyang@gmail.com.

Gongping Liu, Department of Pathophysiology, School of Basic Medicine, Key Laboratory of Education Ministry of China/Hubei Province for Neurological Disorders, Tongji Medical College, Huazhong University of Science and Technology, Wuhan 430030, China. Email: liugp111@mail.hust.edu.cn.

Yongmei Xie, State Key Laboratory of Biotherapy and Cancer Center, West China Hospital, Sichuan University and Collaborative Innovation Center of Biotherapy, Chengdu 610041, China. Email: xieym@scu.edu.cn.

This file includes:

Supplemental Methods

Supplemental Figure 1 to Supplemental Figure 17

Supplemental Table 1 to Supplemental Table 3

Supplemental Video legends

Supplemental Methods

Animal treatment

To elucidate the involvement of mitophagy, we performed an additional experiment using Mdivi-1 (HY-15886, MCE, USA) as a pharmacological tool. 13-week-old male SOD1^{G93A} mice were treated with either UA-30 (100 mg/kg) or saline for 6 weeks. Starting at week 3, mice received daily intraperitoneal injections of Mdivi-1 (40 mg/kg) for four consecutive weeks. Mdivi-1 was administered intraperitoneally 1h prior to the daily UA-30 administration.

Survival analysis

For survival studies, male and female SOD1^{G93A} mice were assigned to saline, UA-30 (100 mg/kg), or Riluzole (10 mg/kg) groups. Daily oral gavage was initiated at 13 weeks of age and continued until natural death or humane euthanasia. The primary endpoint of the longevity study was natural death or euthanasia according to predefined humane endpoints. Euthanasia was performed in mice judged unlikely to survive for more than 48 h and showing severe distress. Euthanasia decisions were made following independent blinded veterinary assessment in accordance with AAALAC International guidelines and were based strictly on animal health status. Humane endpoint criteria included severe lethargy, rapid weight loss (>20% within two weeks), significant abdominal distension, signs indicative of pain based on body condition scoring, unresponsiveness to stimuli, severe ulceration or hemorrhagic tumors, marked hypothermia, and abnormal respiratory rates.

Behavioral tests

Behavioral tests were conducted at baseline prior to treatment initiation and subsequently every two weeks during the 6-week treatment period.

Gait analysis: Gait analysis was employed to detect movement abnormalities. Data acquisition utilized the CatWalk XT gait analysis system (Noldus, Netherlands). The apparatus consisted of a 1.3-meter-long enclosed walkway featuring a 70-cm glass plate floor. A high-speed camera mounted beneath this illuminated glass plate captured ventral views of paw contacts. Animals were tested within a darkened, enclosed corridor (approximately 5 cm wide) constructed around the glass plate. The total testing area (~10 × 20 cm) was available for animals to move around freely. Animals were acclimatized to the apparatus 24 h prior to testing. During the experiment, mice were encouraged to voluntarily cross the walkway. Video recordings captured sequences containing at least three consecutive complete gait cycles for subsequent analysis. All footprints were manually classified and labelled according to anatomical location (left/right forelimb, left/right hindlimb). After classification, the system software automatically calculated and synthesized gait parameters.

Climbing-pole test: The climbing-pole test was employed to assess motor coordination in mice. The apparatus consisted of a vertical wooden pole (50 cm height, 1.0 cm diameter) wrapped with gauze to increase surface friction. Each mouse was positioned at the apex of the pole facing downward, and the time required to descend to the base was recorded. Prior to formal testing, mice underwent daily training sessions over a three-day period. During the assessment phase, each trial was limited to a maximum

duration of 15 s. Three consecutive trials were performed per mouse, and the mean descent latency was calculated for subsequent statistical analysis.

Wire hanging test: Forelimb and hindlimb muscle strength was assessed using the wire grid hanging test. The apparatus consisted of a 50 cm × 50 cm wire grid. Each mouse was placed in the center of the grid. After the mouse grasped the wires, the grid was rotated 180°. The duration the mouse remained hanging on the grid was recorded. All mice received pre-training prior to testing. Each mouse underwent two trials, with the maximum duration per trial set at 180 s. The mean hanging duration was calculated for each animal.

Rotarod test: The rotarod test was employed to evaluate endurance, balance, and motor coordination in mice. Each mouse was carefully placed onto the rod of a rotarod fatigue tester, which had a maximum rotation speed of 25 rpm. Prior to formal testing, mice underwent a 3-day training regimen, consisting of two sessions per day, with each training session capped at 180 s. The formal experiment was repeated for three trials, with trials separated by at least 1 h. The mean fall latency time was recorded for each mouse upon loss of balance.

Grip strength test: Maximum limb muscle strength in mice was evaluated with a grip strength apparatus. Individual mice were centered on the grip plate. Following establishment of a firm grasp, the mouse was steadily pulled in a horizontal plane until grip release occurred. Each subject underwent three measurements, and the peak force value was used for assessment.

Blood biochemical analysis

Following the behavioral testing, blood samples were collected from the inferior vena cava and maintained at room temperature (RT) for 1 h. Subsequently, the samples were centrifuged at 3,000 rpm for 10 min to obtain serum. Serum levels of creatine kinase (CK), lactate dehydrogenase (LDH), glucose (GLU), alanine aminotransferase (ALT), aspartate aminotransferase (AST), creatinine (Cr), and blood urea nitrogen (BUN) were measured using a HITACHI 7080 Automatic Clinical Analyzer (Hitachi, Tokyo, Japan).

Histological analysis

Prior to tissue extraction, mice were anesthetized with 2% sodium pentobarbital and subjected to cardiac perfusion with physiological saline. All histological analyses were performed at the experimental endpoint, following completion of the 6-week treatment period and the final behavioral assessment. The gastrocnemius muscles were surgically removed bilaterally and weighed. The L4-L5 lumbar vertebral segments and muscle tissues were harvested and immersed in 4% paraformaldehyde for fixation. After 24 h, the tissues were dehydrated through a graded series of ethanol solutions and subsequently embedded in paraffin. 5 μm-thick paraffin sections were prepared using a microtome (Leica, Germany) for subsequent histological staining.

Hematoxylin and eosin (H&E) staining: Before staining, paraffin sections were deparaffinized in xylene and rehydrated with a dilution series of ethanol. Tissue sections were stained with hematoxylin for 10 min according to the manufacturer's instructions for the Hematoxylin and Eosin (H&E) Staining Kit (C0105, Beyotime,

China). Subsequently, sections were rinsed in running tap water for 10 min and counterstained with eosin for 2 min. Sections were then dehydrated rapidly through a graded ethanol series and mounted with neutral resin mounting medium. Images were acquired using a Leica Aperio GT 450 scanner (Leica, Germany).

Masson staining: Tissue sections underwent Masson's trichrome staining using a Modified Masson's Trichrome Stain Kit (G1346, Solarbio, China). Following standard deparaffinization and hydration, the staining protocol involved sequential incubation in: hematoxylin, Ponceau S fuchsin, phosphomolybdic acid, and aniline blue solutions. Subsequently, sections were dehydrated, cleared, and cover-slipped with neutral resin. Digital images were acquired utilizing a Leica Aperio GT 450 scanner (Leica, Germany).

Sirius red staining: Fibrosis in gastrocnemius muscle tissues was evaluated using Sirius red staining, performed with a commercial kit (G1472, Solarbio, China). Following dewaxing and rehydration, tissue sections were immersed in iron hematoxylin staining solution for 10 min. Subsequent washing under running tap water was carried out for 10 min, followed by staining in Sirius red solution for 8 min. The sections were then dehydrated, cleared, and coverslipped using neutral resin. Imaging was conducted utilizing a Leica Aperio GT 450 scanner (Leica, Germany).

Immunohistochemistry (IHC): Tissue sections underwent deparaffinization and rehydration prior to heat-mediated antigen retrieval using sodium citrate buffer (pH 6.0). To suppress endogenous peroxidase activity, slides were treated with 3% hydrogen

peroxide for 10 min at ambient temperature. Non-specific binding sites were blocked by incubation in 3% bovine serum albumin (BSA) for 1 h. Primary antibodies were applied and incubated overnight at 4°C. Subsequently, sections were exposed to a biotin-conjugated Goat Anti-Polyvalent secondary antibody (DAB Kit, ab64264, Abcam, USA) for 1 h, followed by a 1-h incubation with streptavidin-horseradish peroxidase conjugate. Diaminobenzidine (DAB) substrate was utilized for chromogenic detection. Stained slides were digitally imaged using a Leica Aperio GT 450 scanner (Leica, Germany).

Immunofluorescence (IF): After antigen retrieval, sections were permeabilized and blocked. Primary antibody incubation was carried out overnight at 4°C. Sections were then treated with fluorescent secondary antibodies for 1h at RT. Counterstaining of nuclei was achieved using 4′,6'-diamidino-2-phenylindole (DAPI) (C1002, Beyotime, China) for 5 min at RT. Confocal microscopy images were acquired using a Zeiss LSM980 system (Zeiss, Germany). Antibodies used are listed in Supplemental Table 2.

Neuromuscular junction (NMJ) staining: NMJ staining was performed on longitudinal cryosections of gastrocnemius muscle. Fresh muscle tissues were embedded in OCT and sectioned at 30 μm thickness using a cryostat (Leica, Germany). Sections were permeabilized and blocked, followed by overnight incubation at 4°C with primary antibody against synaptophysin. Sections were then incubated with fluorescent secondary antibodies and Alexa Fluor 488-conjugated α-bungarotoxin (α-BTX; B13422, Invitrogen, USA) to visualize postsynaptic motor endplates. Confocal images

were acquired using a Zeiss LSM980 system (Zeiss, Germany). Antibodies used are listed in Supplemental Table 2.

Nissl staining: Following dewaxing and rehydration, the sections were stained with 0.5% toluidine blue solution (G1036, Servicebio) for 5 min. Subsequently, the sections were rapidly dehydrated through an ethanol gradient, cleared in xylene and mounted with neutral resin. Images were acquired using a Leica Aperio GT 450 scanner (Leica, Germany).

Cell culture and transfection

PC12 cells were cultured at 37°C under 5% CO_2 in RPMI-1640 medium (C11875500, Gibco, USA) supplemented with 10% FBS. Cells expressing human *SOD1* carrying the G93A mutation were used as a cellular model of ALS associated with mutant SOD1 expression. Cells were seeded into 10 cm dishes or multi-well plates and transiently transfected using Lipofectamine™ 2000 transfection reagent (11668019, Invitrogen, USA) at a DNA-to-reagent ratio of 3:1 (1 μg DNA per well). Plasmids encoding $hSOD1^{G93A}$ (pcDNA3.1(+)-SOD1(G93A)-ECFP-3Xflag) or empty vector （pcDNA3.1(+)-ECFP-3xFLAG），and constitutively active $RalA^{G23V}$ (pcDNA3.1-CMV-3xFLAG-Rala(G23V)-EF1α-EGFP) or corresponding empty vector (pcDNA3.1-CMV-3xFLAG-EF1α-EGFP), were synthesized by OBiO Biologic Technology Corp., Ltd. (Shanghai, China). At 24 h post-transfection, the medium was replaced with serum-free medium containing either 100 μM UA-30 or vehicle. Cells were harvested 48 h post-transfection for subsequent experiments.

Western blotting (WB)

Protein extraction from tissues or cells was performed utilizing RIPA lysis buffer supplemented with protease inhibitors. Protein quantification was carried out following the manufacturer's protocols provided with the BCA assay kit (23227, Thermo Fisher Scientific, USA). Subcellular fractionation, isolating cytoplasmic and nuclear proteins, was achieved using the Nucl-Cyto-Mem Preparation Kit (P1201, Applygen, Beijing). Mitochondrial and cytosolic fractions from spinal cord tissues were isolated using a mitochondria extraction kit (C3606, Beyotime, China) according to the manufacturer’s instructions. Following separation on 10% SDS-PAGE gels, proteins were electrophoretically transferred onto PVDF membranes. Blocking of membranes was performed with 5% skim milk at RT for 1 h. Subsequently, membranes were incubated with primary antibodies overnight at 4°C. The following day, membranes underwent three washes with TBST buffer solution prior to incubation with secondary antibodies at RT for 1 h. Signal visualization was accomplished using the Pierce™ ECL Western Blotting Substrate kit (32209, Thermo Fisher Scientific, USA). ImageJ software facilitated quantitative densitometric analysis. Specific antibodies employed are detailed in Supplemental Table 2.

Immunoprecipitation (IP)

Cells or spinal cord tissues were lysed on ice using WB/IP lysis buffer (P0013, Beyotime, China) containing a protease inhibitor. For each sample, 200 μl of the lysate was incubated with the target-specific antibody or an equivalent amount of species-matched control IgG overnight at 4°C under constant rotation. The remaining lysate

was retained as the input control. 10 µl of Protein A/G magnetic beads (pre-equilibrated with three washes in ice-cold PBS containing inhibitor) were added to the antibody-lysate mixture and incubation was continued under rotation at 4°C for 6 h. The bead-bound complexes were then immobilized on a magnetic rack, washed three times with the same ice-cold PBS/inhibitor solution, and reconstituted in 40 µl of 1× SDS-PAGE loading buffer. Proteins were eluted by denaturation at 95°C for 10 min. Following magnetic separation, the supernatant containing the immunoprecipitated proteins was collected for subsequent WB analysis.

Quantitative real-time PCR (RT-qPCR)

Total RNA was extracted from spinal cord or PC12 cells with TRIZOL Reagent (Invitrogen, Germany). Reverse transcription of 0.5 µg RNA into cDNA was performed using the Evo M-MLV kit (AG11706, Accurate Biology, China). Quantitative assessment of gene expression was then achieved by real-time PCR (Thermo Fisher Scientific instrument) with SYBR Premix Ex Taq™ (Takara, Japan) and gene-specific primers. Expression levels were normalized to the housekeeping gene *Gapdh* and calculated relative to controls using the $2^{-\Delta\Delta Ct}$ method. The sequences of all primers used in this study are listed in Supplemental Table 3.

Mitochondrial functional assays

Mitochondrial functional assays were performed on tissues collected at the experimental endpoint after completion of behavioral testing.

ATP assay: ATP levels in the mouse medulla oblongata, spinal cord, and gastrocnemius

muscles were measured using an ATP Assay Kit (S0026, Beyotime, China) according to the manufacturer's instructions. Tissues were homogenized in the provided ATP lysis buffer to prepare extracts. Following protein concentration determination via BCA assay, equivalent amounts of sample protein or ATP standards were combined with the ATP detection reagent. The ATP-dependent bioluminescence was quantified using a microplate reader (Tecan, Switzerland). Final ATP concentrations in the samples were derived by interpolation from the concurrently generated ATP standard curve. ATP content is expressed as absolute values (nmol/mg protein), reflecting steady-state intracellular ATP levels.

Malondialdehyde (MDA) assay: MDA, as a marker of lipid peroxidation, was quantified in mouse tissue homogenates using a commercial assay kit (S0131S, Beyotime, China). Following the manufacturer's protocol, tissue homogenates underwent centrifugation (12,000×g, 10 min, 4 °C) to collect the supernatant. Protein concentration in the supernatant was determined using the BCA assay. Subsequently, aliquots of supernatant (100 μl) were mixed with the MDA detection reagent (200 μl) and heated at 100 °C for 15 min. After cooling, the reaction mixtures were centrifuged (1,000×g, 10 min, RT). The resulting supernatant (200 μl) was then transferred to a 96-well microplate. Absorbance at 532 nm was recorded using a microplate reader (Tecan, Switzerland). MDA concentrations in the samples were derived by interpolation from a concurrently generated standard curve.

Superoxide dismutase (SOD) activity assay: A SOD Assay Kit (S0103, Beyotime,

China) was used for the measurement of the total SOD activity in the medulla oblongata, spinal cord, and gastrocnemius muscles. Absorbance at a wavelength of 450 nm was measured a microplate reader (Tecan, Switzerland).

ROS and mitochondrial ROS (mitoROS) assessment: Cells were loaded with the fluorescent indicator 2',7'-dichlorodihydrofluorescein diacetate (DCFH-DA; Sigma-Aldrich, USA) at a concentration of 10 μM. Incubation proceeded for 30 min at 37°C in serum-free medium. To specifically detect mitochondrial superoxide, cells were treated with 5 μM MitoSOX Red reagent (40778ES50, Yeasen Biotechnology, China). This incubation was carried out for 10 min at 37°C using serum-free medium. Following incubation, cells were washed three times with PBS. Fluorescent images were captured using a laser confocal microscope (LSM980, Zeiss, Germany). Fluorescence intensity was measured using a microplate reader (Tecan, Switzerland): ROS detection was performed at 488 nm excitation and 525 nm emission, while mitoROS was assessed at 510 nm excitation and 580 nm emission.

Measurement of mitochondrial membrane potential (MMP): Changes in cellular MMP were assessed using the JC-1 mitochondrial membrane potential detection kit (C003S, Beyotime, China). Cells were washed three times with PBS and then incubated directly with JC-1 dye for 20 min. Fluorescent images were acquired using a laser confocal microscope (LSM980, Zeiss, Germany). The fluorescence intensity of JC-1 monomers (excitation/emission: 514/529 nm) and aggregates (excitation/emission: 585/590 nm) was measured using a microplate reader (Tecan, Switzerland). Quantification of MMP

was based on the fluorescence intensity ratio of JC-1 aggregates to monomers.

Transmission electron microscopy (TEM)

The spinal cord was surgically removed and immersed in a 2.5% glutaraldehyde solution overnight at 4°C. The tissue blocks (1-2 mm^3) were treated with 1% osmium tetroxide (OsO_4) for 1 h, followed by dehydration using graded alcohol and embedding in epoxy resin. At 80°C, polymerization of the specimen was performed for 24 h. Staining of ultrathin sections (60-70nm) employed 2% uranyl acetate followed by 1% lead citrate; these were examined with a Hitachi 7100 transmission electron microscope (Nikon, Tokyo, Japan).

CCK-8 assay

PC12 cells were seeded into 96-well plates and cultured under standard conditions. Once cellular attachment was achieved, cells were cultured in FBS-free medium with various UA-30 concentrations for 24 h. To assess cell viability, 10 µl of CCK-8 solution (CK04, Dojindo, Japan) were introduced into each well, and the plates were incubated at 37 °C for an additional 1-4 h. Absorbance at 450 nm was subsequently recorded using a Tecan microplate reader (Switzerland). The obtained values were directly correlated with cell viability.

Proteomics

Protein isolation and digestion: Tissues were harvested from each experimental mouse cohort, with four biological replicates per group. Samples were homogenized on ice in

lysis buffer (8 M urea, PBS pH 8.0) supplemented with protease and phosphatase inhibitors. Following homogenization, lysates were centrifuged (12,000 rpm, 30 min, 4°C) to clarify the supernatant. Supernatant protein concentration was determined using a BCA assay. Aliquots containing 50 μg of protein per sample were processed for enzymatic digestion. Proteins were reduced with 10 mM dithiothreitol (DTT, 55°C, 1 h) and subsequently alkylated using 25 mM iodoacetamide (IAA, RT, 1 h, protected from light). Trypsin (Promega, WI, USA; 1:25 enzyme-to-protein ratio) was added for proteolysis (37°C, 15 h). The digest was acidified to pH 1-2 with trifluoroacetic acid (TFA), and resulting peptides were purified via solid-phase extraction using a reverse-phase Oasis HLB column (Waters, USA). Purified peptides were lyophilized and prepared for TMT labeling.

TMT labeling and LC-MS/MS: Lyophilized peptides were dissolved in 50 μl of 200 mM triethylammonium bicarbonate (TEAB) and incubated with Tandem Mass Tag (TMT) reagents (90110, Thermo Fisher Scientific, USA) for 1 h at RT. The labeling reaction was terminated by adding 5% hydroxylamine (15 min incubation). All TMT-labeled samples were combined, desalted, dried, and reconstituted in 100 μl of 0.1% formic acid. Peptide fractionation was performed using an Ultimate 3000 UHPLC system (Thermo Fisher Scientific, USA) equipped with an Xbridge BEH 300 C18 column (Waters, USA), yielding 60 fractions as per manufacturer guidelines. Fractions were dried, resuspended in 20 μl of 0.1% formic acid, and analyzed by LC-MS/MS. MS/MS spectra were matched to the UniProt Mouse proteome database using Proteome Discoverer software (Thermo Fisher Scientific, USA). Relative protein quantification

was derived from peptide ion intensities. Proteins exhibiting differential expression between groups were identified using the Student t-test (p-value < 0.05) within Perseus software.

Bioinformatic assessment: Differentially expressed proteins identified in Perseus underwent further analysis. Protein abundance patterns were analyzed by unsupervised hierarchical clustering based on normalized expression values and visualized using heatmaps. Functional annotation was performed via Gene Ontology (GO) analysis using the DAVID Bioinformatics Resource (v6.8). Figures were generated and refined using the Hiplot visualization platform.

LC-MS/MS analysis

Mouse spinal cord and brain samples were collected at specific time intervals following a single oral gavage administration of 100mg/kg UA-30. Tissue homogenates were prepared at a ratio of 1 g of tissue to 3 ml of extraction solvent (phosphate: methanol: 15 mM phosphate buffer = 3:40:80, v/v). These homogenates were then centrifuged at 10,000×g for 20 min at 4°C to obtain the supernatant, which was subsequently analyzed using LC-MS/MS.

Chromatographic separation was carried out on an LC-MS/MS system (API6500+ ABSciex ExionAC: SH-LCMS-006). The mobile phase used was composed of water with 0.5% formic acid (mobile phase A) and acetonitrile with 0.1% formic acid (mobile phase B). The separation of analytes was accomplished using an XSelect HSS T3 column (4.6 × 50 mm, 3.5 µm; Waters, USA) that was kept at 40°C. A gradient elution

procedure was used, with a flow rate of 0.8 ml/min and an injection volume of 10 μl. The target compound (UA-30) was monitored using the mass transition of m/z 481.1 → 127.0 (precursor ion → product ion) in multiple reaction monitoring (MRM) mode using optimized mass spectrometry parameters: curtain gas (CUR) 40 psi, collision gas (CAD) 10 psi, ion spray voltage (IS) 5500 V, nebulizer temperature (TEM) 500 °C, gas 1 (GS1) 50 psi, gas 2 (GS2) 50 psi, entrance potential (EP) 10 V, and collision cell exit potential (CXP) 10 V.

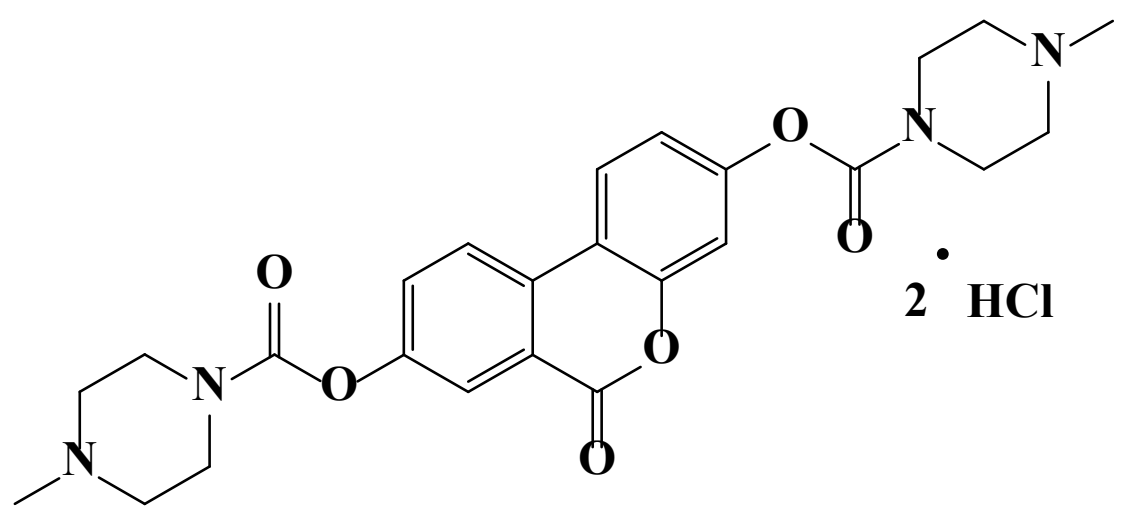

Supplemental Figure 1 Chemical structure of UA-30.

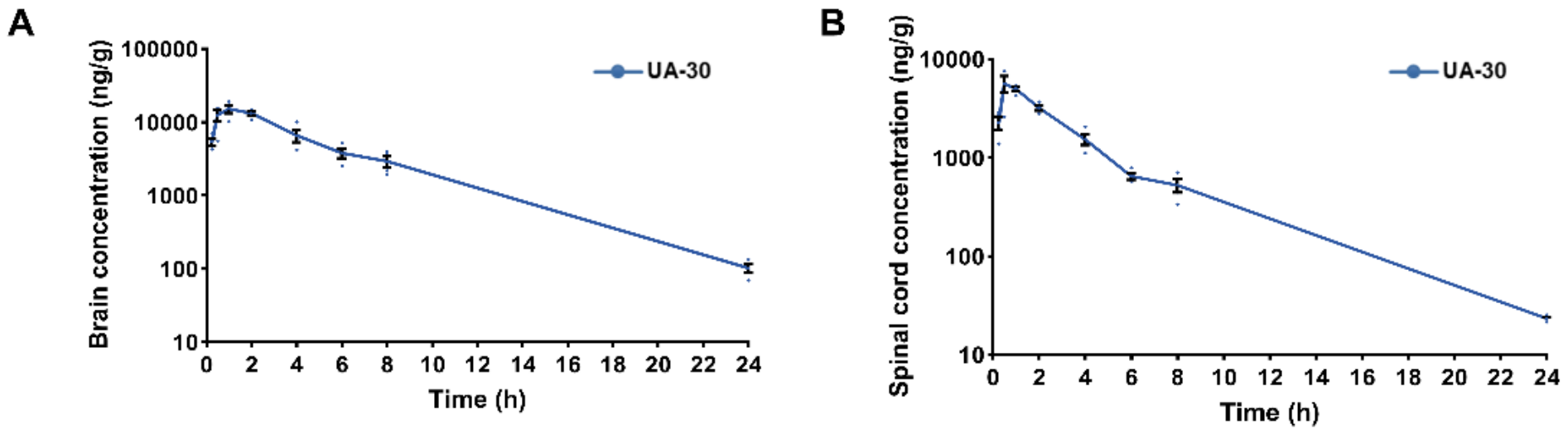

Supplemental Figure 2 Concentration-time curve of UA-30 in brain and spinal cord.

(A, B) Concentration-time curve of UA-30 in brain tissue (A) and spinal cord (B) after oral administration of 100 mg/kg UA-30 in mice. n = 4 mice per time point. Data are expressed as mean ± SEM.

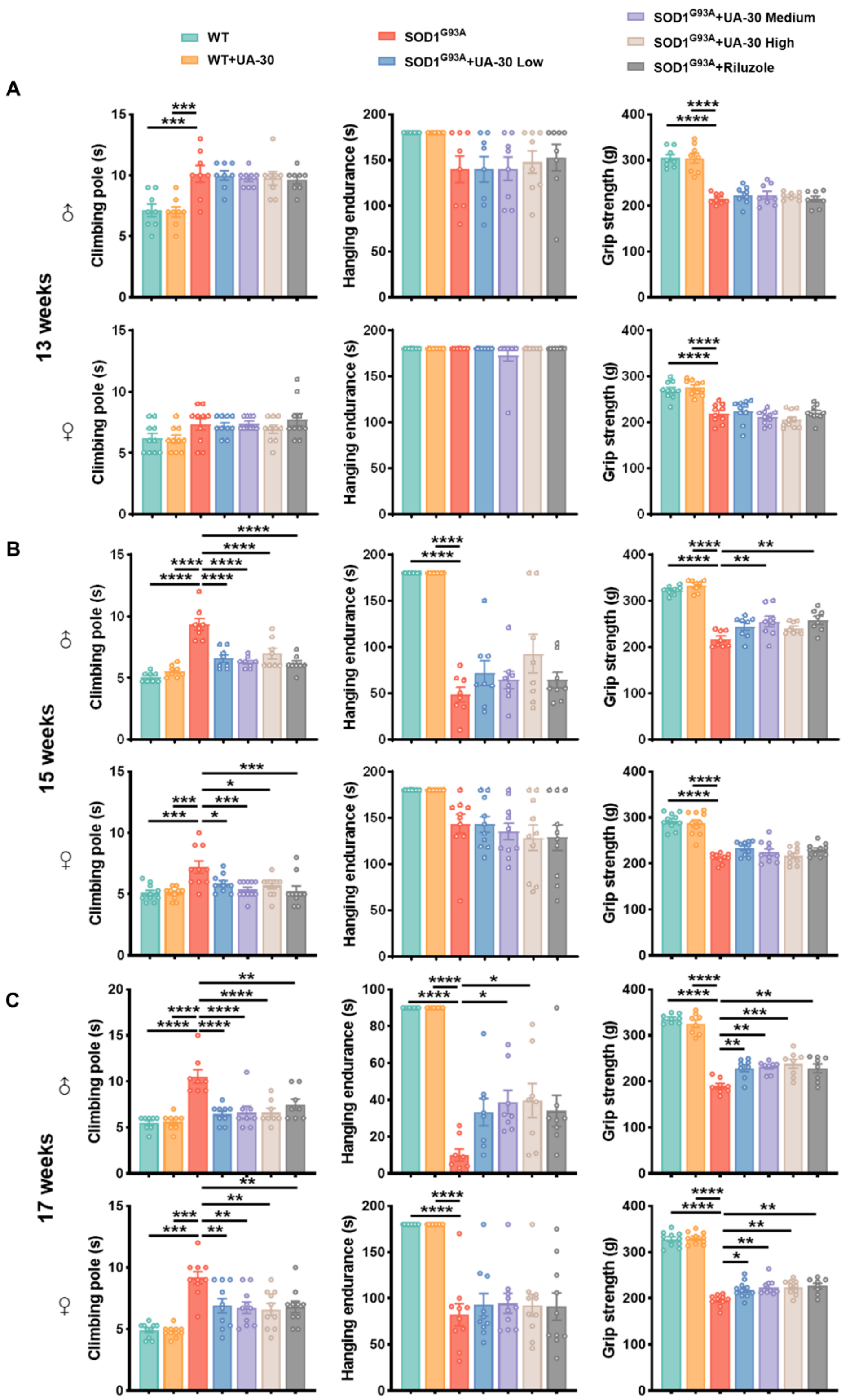

Supplemental Figure 3 UA-30 treatment improved motor performance in SOD1^{G93A} mice.

(A) Baseline behavioral assessments at 13 weeks of age, prior to treatment initiation,

including the climbing pole test, hanging wire test, and grip strength test in male and female mice. (B) Behavioral assessments after 2 weeks of treatment (15 weeks of age), including the climbing pole test, hanging wire test, and grip strength test in male and female mice. (C) Behavioral assessments after 4 weeks of treatment (17 weeks of age), including the climbing pole test, hanging wire test, and grip strength test in male and female mice. n = 8-10 per group for each sex. Data are expressed as mean ± SEM. *, $P < 0.05$, **, $P < 0.01$ ***, $P < 0.001$ and ****, $P < 0.0001$. One-way ANOVA followed by Tukey's multiple-comparison test.

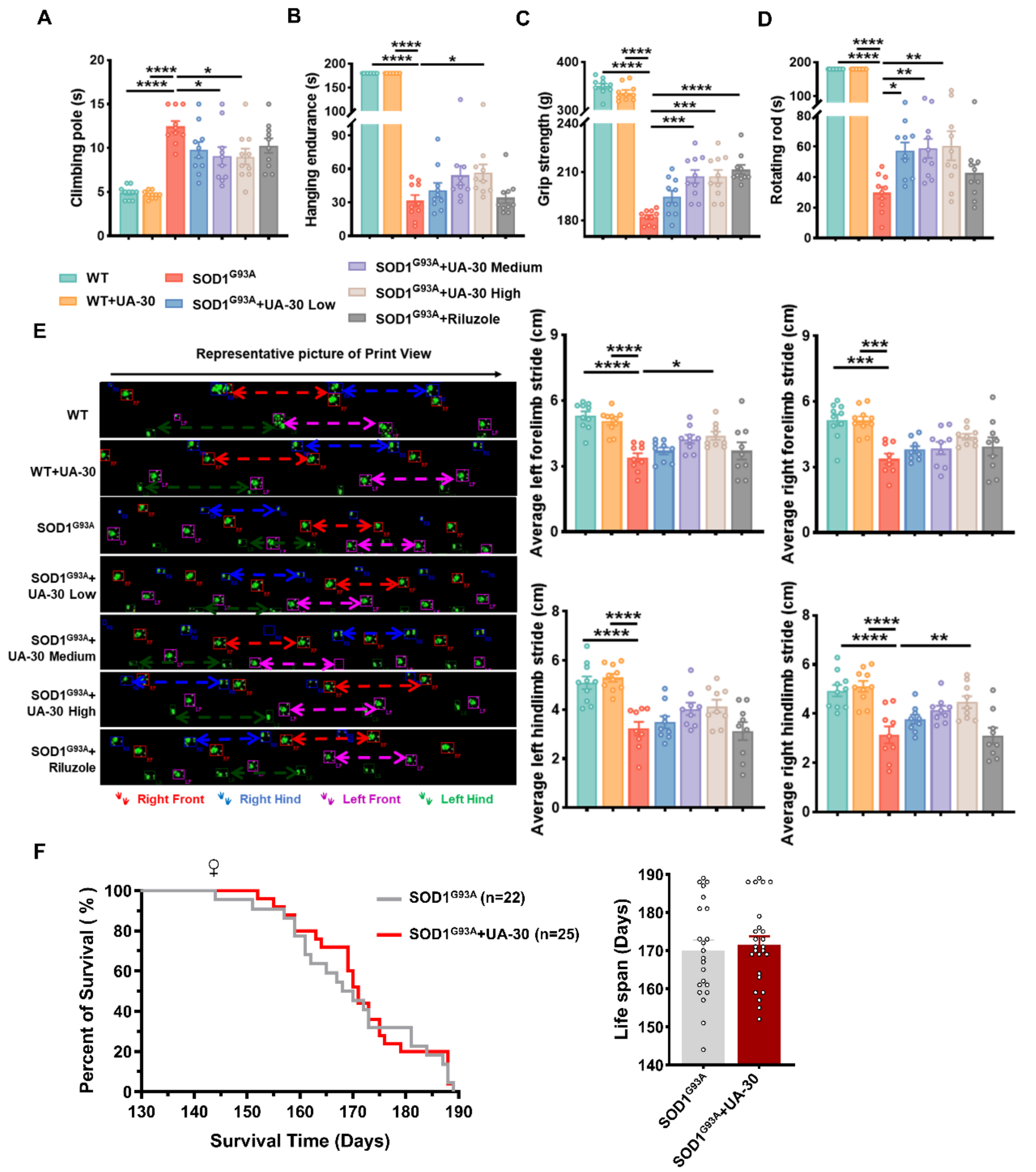

Supplemental Figure 4 UA-30 treatment attenuated motor dysfunction in female SOD1^{G93A} mice.

(A-E) Motor performance and muscular strength assessments in female SOD1^{G93A} mice treated with different doses of UA-30 or saline for 6 weeks. Tests performed: (A) Climbing pole test, (B) Hanging wire test, (C) Grip strength test, (D) Rotarod test. (E) Gait testing. Left/right forelimb and left/right hindlimb stride length was recorded

during gait analysis. n = 9-10 female per group. (F) Kaplan-Meier survival curves for female $SOD1^{G93A}$ mice treated with saline or UA-30 (100 mg/kg). Statistical summaries show overall lifespan for each cohort. n=22 $SOD1^{G93A}$ female mice. n=25 $SOD1^{G93A}$+UA-30 female mice. Data are expressed as mean ± SEM. *, $P < 0.05$, **, $P < 0.01$ ***, $P < 0.001$ and ****, $P < 0.0001$. A-E, one-way ANOVA followed by Tukey's multiple comparison test; F, log-rank (Mantel-Cox) test for survival curves and one-way ANOVA followed by Tukey's multiple comparison test for overall lifespan summary.

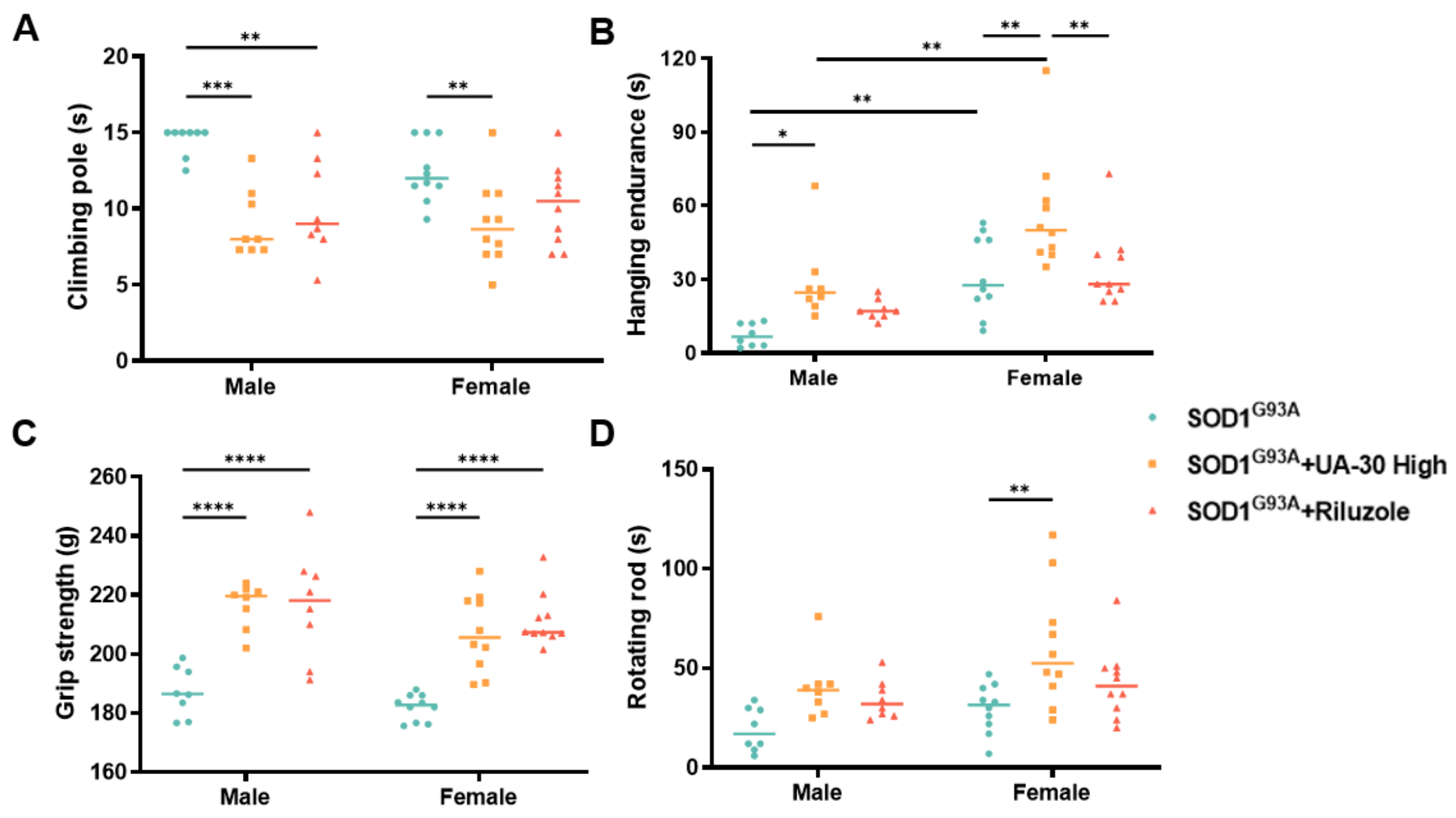

Supplemental Figure 5 Sex-stratified behavioral effects of UA-30 and Riluzole in $SOD1^{G93A}$ mice.

(A-D) Behavioral performance was analyzed separately in male and female $SOD1^{G93A}$ mice treated with vehicle, UA-30 High (100 mg/kg), or Riluzole (10 mg/kg). Shown are the climbing pole test (A), hanging wire test (B), grip strength test (C), and rotarod test (D). Statistical comparisons shown in the panels indicate treatment comparisons within each sex and were performed using two-way ANOVA followed by Šídák's multiple comparisons test. To assess whether treatment responses differed by sex, two-way ANOVA was performed using Sex and Treatment as factors. No significant Sex × Treatment interaction was detected for pole climbing (P = 0.3222), hanging wire (P = 0.5612), grip strength (P = 0.8287), or rotarod performance (P = 0.5821). n = 8-10 mice per group for each sex. Data are expressed as mean ± SEM. *, $P < 0.05$, **, $P < 0.01$, ***, $P < 0.001$ and ****, $P < 0.0001$.

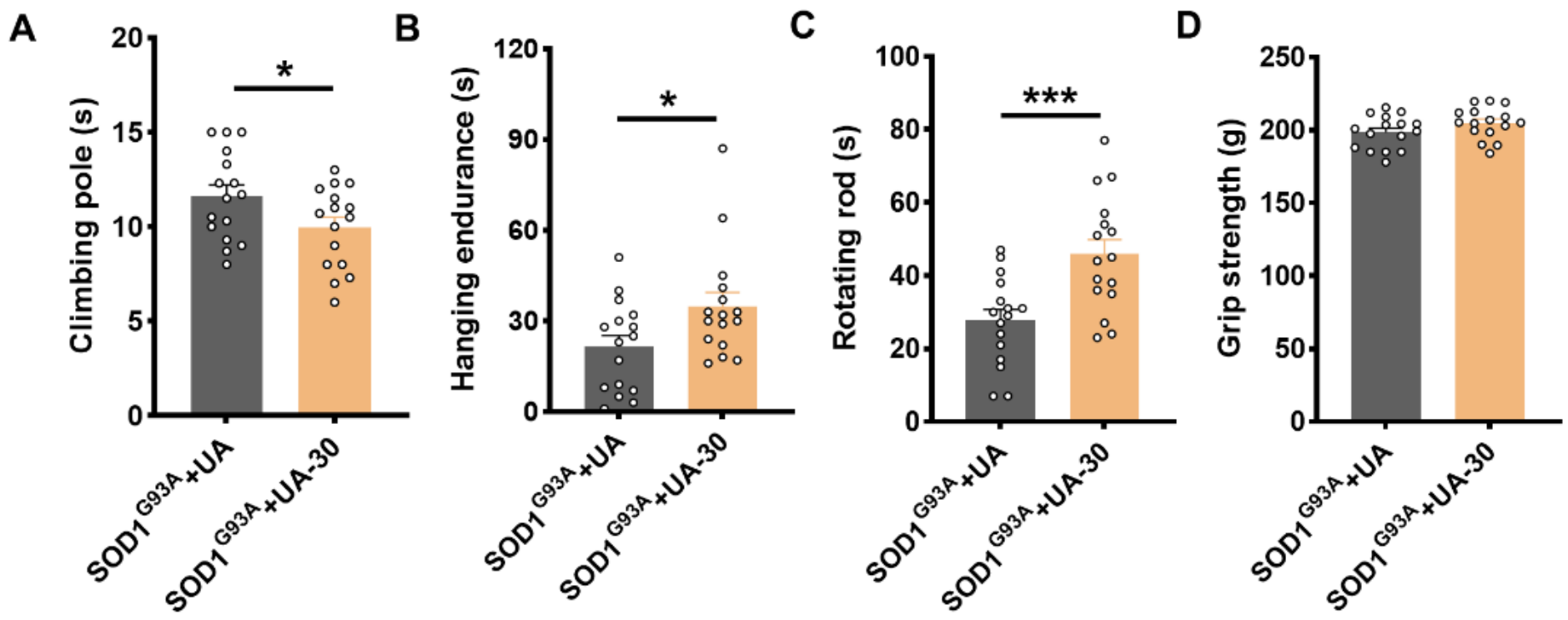

Supplemental Figure 6 UA-30 outperforms UA in motor function tests at equimolar doses in SOD1^{G93A} mice.

(A-D) Behavioral assessment of motor function and muscle strength in SOD1^{G93A} mice treated with UA-30 (25 mg/kg) or an equimolar concentration of UA (10 mg/kg). (A) Climbing pole test, (B) Hanging wire test, (C) Rotarod test, (D) Grip strength test. n = 16 per group (8 males and 8 females). Data are expressed as mean ± SEM. *, $P < 0.05$ and ***, $P < 0.001$. Two-tailed unpaired Student's t test.

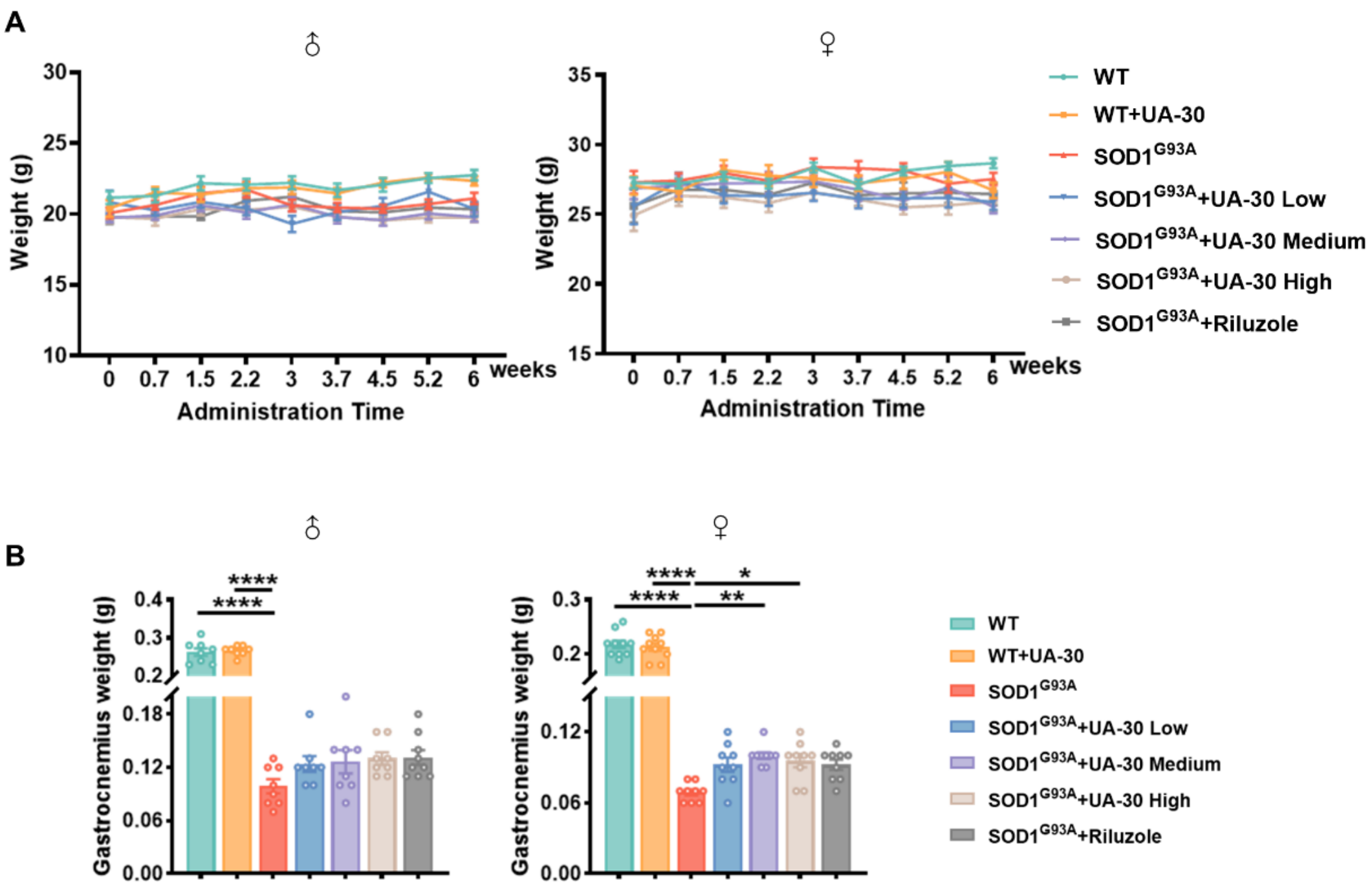

Supplemental Figure 7 Effects of UA-30 treatment on body weight and gastrocnemius muscle weight in SOD1^{G93A} mice.

(A) Changes in body weight of mice treated with UA-30 or saline over the course of 6 weeks in each group (left) male (right) female. (B) Weight of the gastrocnemius muscle of mice treated with UA-30 or saline in each group (left) male (right) female. n = 8-10 per group for each sex. Data are expressed as mean ± SEM. *, $P < 0.05$, **, $P < 0.01$ and ****, $P < 0.0001$. A, two-way ANOVA followed by Bonferroni's multiple comparison test; B, One-way ANOVA followed by Dunnett's multiple comparison test.

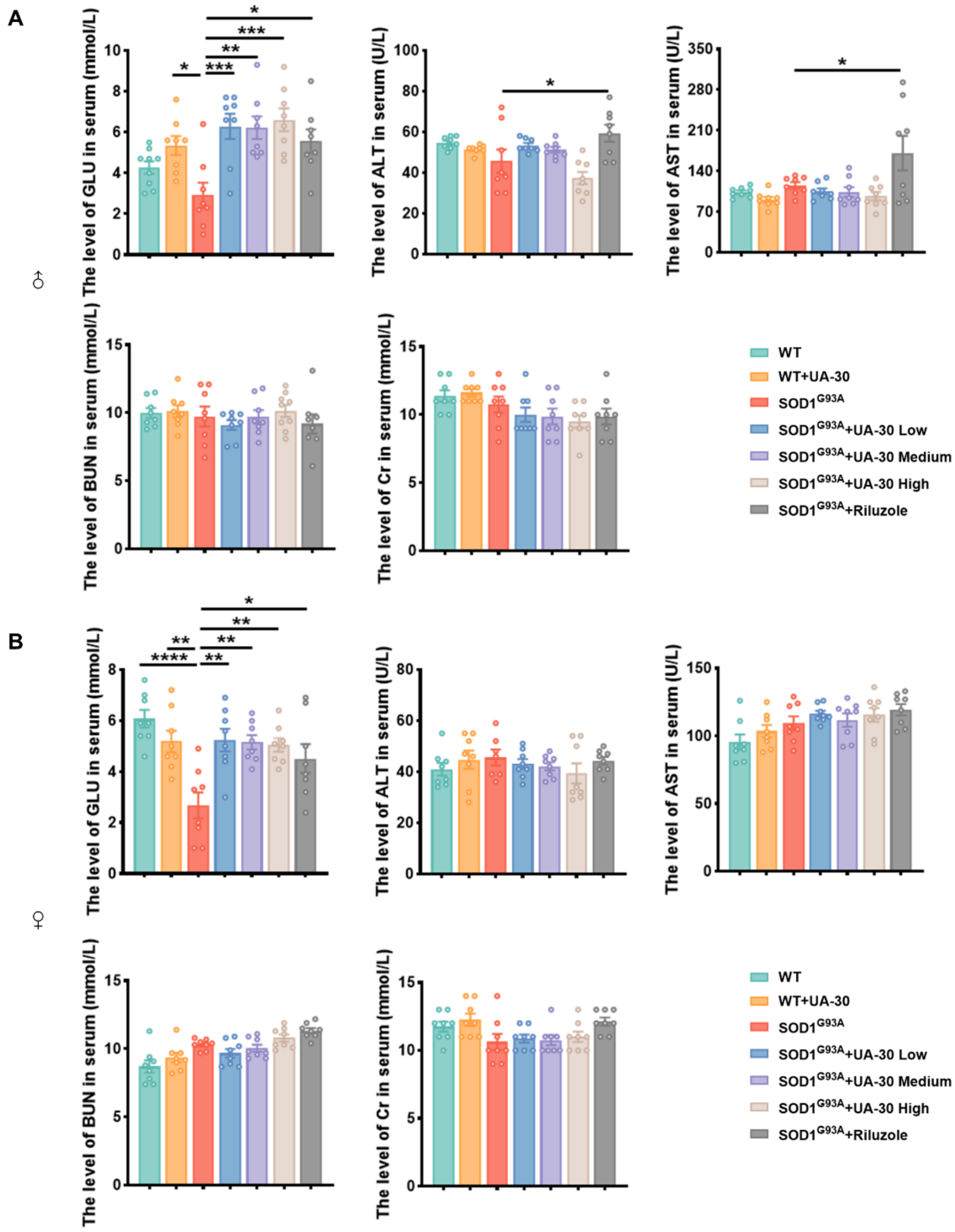

Supplemental Figure 8 Serum biochemical parameters in UA-30-treated SOD1^{G93A} mice.

(A, B) Blood biochemical analysis of glucose (GLU), alanine aminotransferase (ALT), aspartate aminotransferase (AST), blood urea nitrogen (BUN), and creatinine (Cr) in SOD1^{G93A} mice treated with UA-30 or saline in each group (A) male (B) female. n = 8 per group for each sex. Data are expressed as mean ± SEM. *, $P < 0.05$, **, $P < 0.01$ ***, $P < 0.001$ and ****, $P < 0.0001$. One-way ANOVA followed by Dunnett's multiple

comparison test.

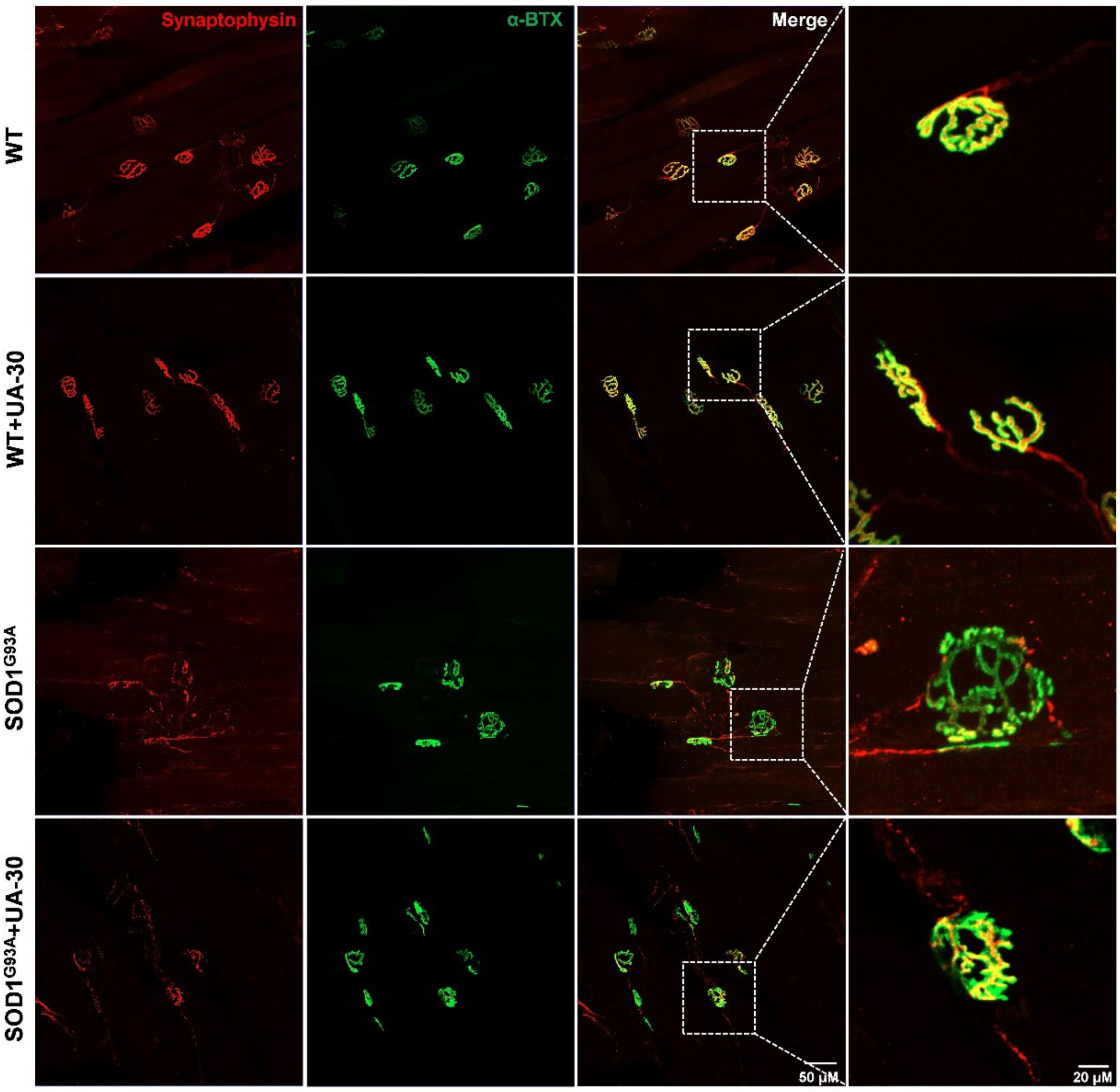

Supplemental Figure 9 UA-30 partially preserved neuromuscular junction innervation in the gastrocnemius muscle of male SOD1^{G93A} mice.

Representative immunofluorescence images of neuromuscular junctions stained for synaptophysin (red) and α-bungarotoxin (α-BTX, green) in UA-30 (100 mg/kg)-treated male mice. Merged images show the apposition between presynaptic terminals and postsynaptic motor endplates.

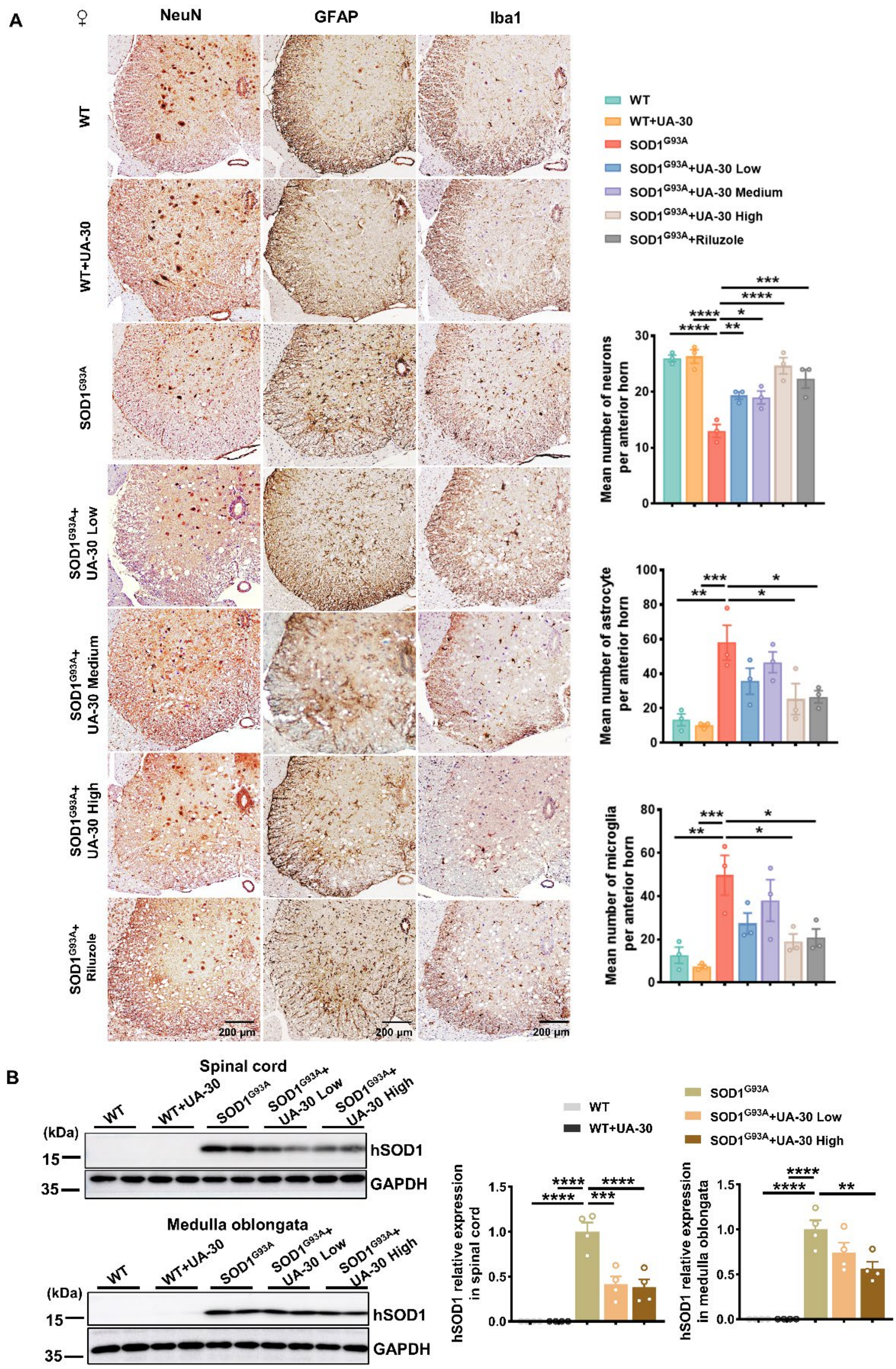

Supplemental Figure 10 UA-30 treatment attenuated motor neuron loss and neuroinflammation and reduced the expression of hSOD1 in SOD1^{G93A} mice.

(A) Representative images and quantification of neurons (NeuN), astrocytes (GFAP)

and microglia (Iba1) immunohistochemical staining in spinal cord after UA-30 treatment in female SOD1^{G93A} mice. n = 3 female per group. (B) The expression levels of hSOD1 in the spinal cord and medulla oblongata of male SOD1^{G93A} mice treated with UA-30 were detected by western blotting and quantitative analysis. n = 4 male per group. Data are expressed as mean ± SEM. *, $P < 0.05$, **, $P < 0.01$ ***, $P < 0.001$ and ****, $P < 0.0001$. One-way ANOVA followed by Dunnett's multiple comparison test.

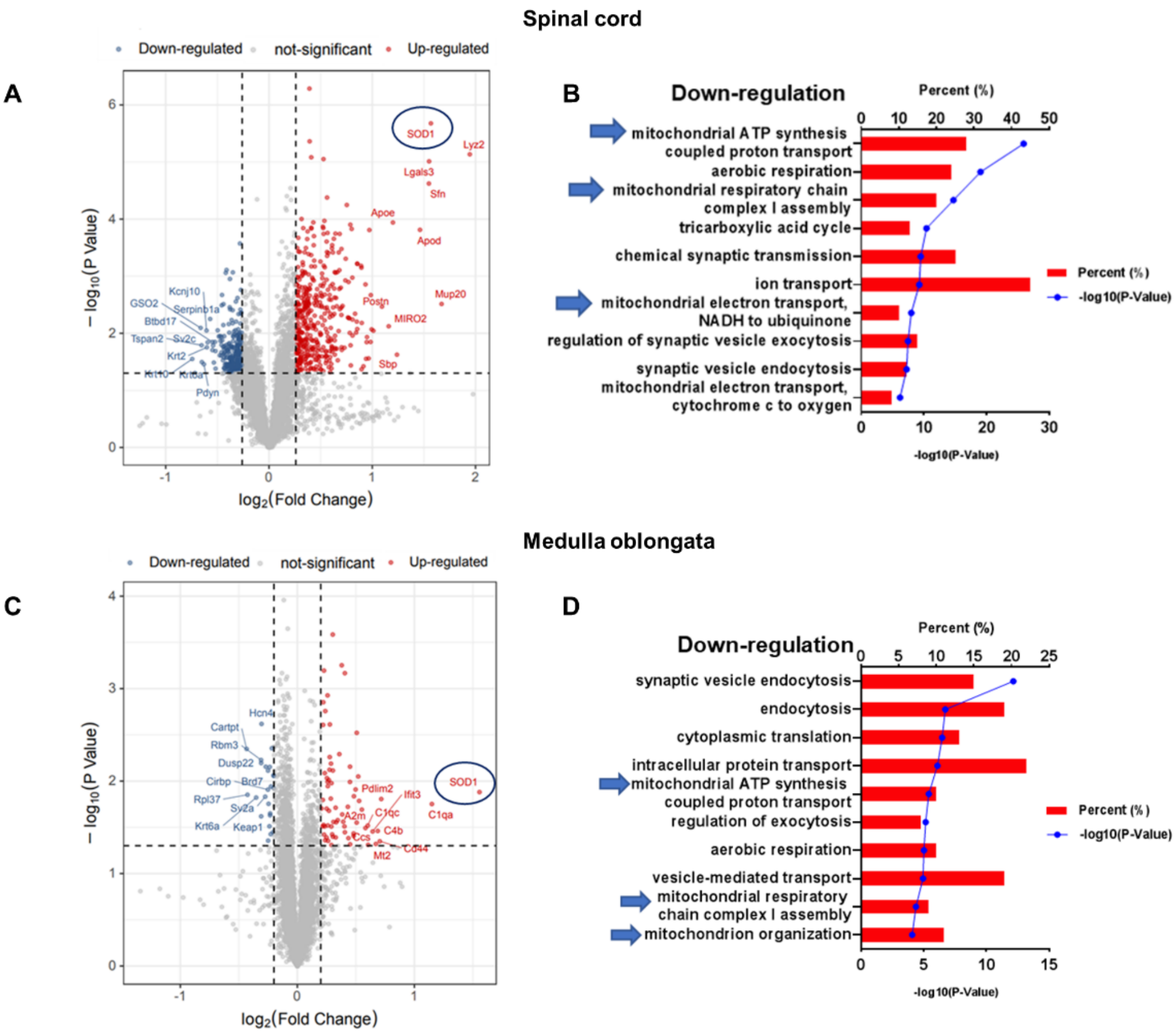

Supplemental Figure 11 Proteomics analysis of the spinal cord and medulla oblongata in male SOD1 ^{G93A} mice.

(A) Volcano plot of differentially expressed (DE) proteins in the spinal cord proteome of SOD1^{G93A} mice compared to WT mice. Red and blue dots represent upregulated and downregulated proteins, respectively. (B) Biological processes enriched among the downregulated DE proteins were defined using $P < 0.05$ in the spinal cord of SOD1^{G93A} mice compared to WT mice. (C) Volcano plot of differentially expressed (DE) proteins in the medulla oblongata proteome of SOD1^{G93A} mice compared to WT mice. Red and blue dots represent upregulated and downregulated proteins, respectively. (D) Biological processes enriched among the downregulated DE proteins were defined using $P < 0.05$ in the medulla oblongata of SOD1^{G93A} mice compared to WT mice.

Arrows indicated terms/pathways with high enrichment scores and greater biological relevance.

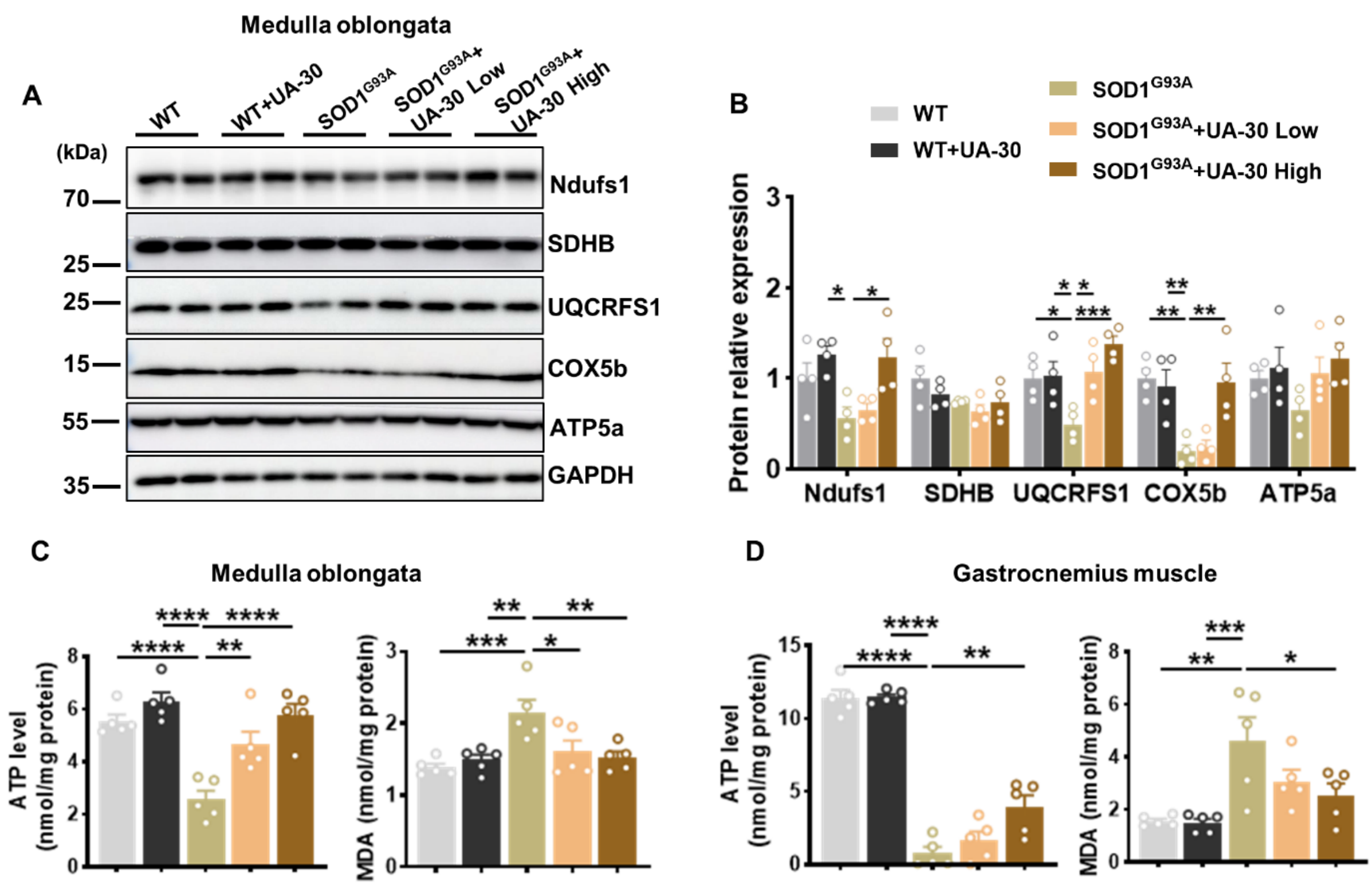

Supplemental Figure 12 UA-30 treatment restored mitochondrial function in SOD1^{G93A} mice.

(A, B) The expression and quantification of mitochondrial respiratory complex I (Ndufs1), II (SDHB), III (UQCRFS1), IV (COX5b), V (ATP5a) in the medulla oblongata from saline or UA-30 treated mice. n = 4 male per group. (C, D) Steady-state ATP content and lipid peroxidation levels were measured in medulla oblongata (C) and gastrocnemius muscle (D) of SOD1^{G93A} mice exposed to different treatments. n = 5 male per group. Data are expressed as mean ± SEM. *, $P < 0.05$, **, $P < 0.01$ ***, $P < 0.001$ and ****, $P < 0.0001$. One-way ANOVA followed by Dunnett's multiple comparison test.

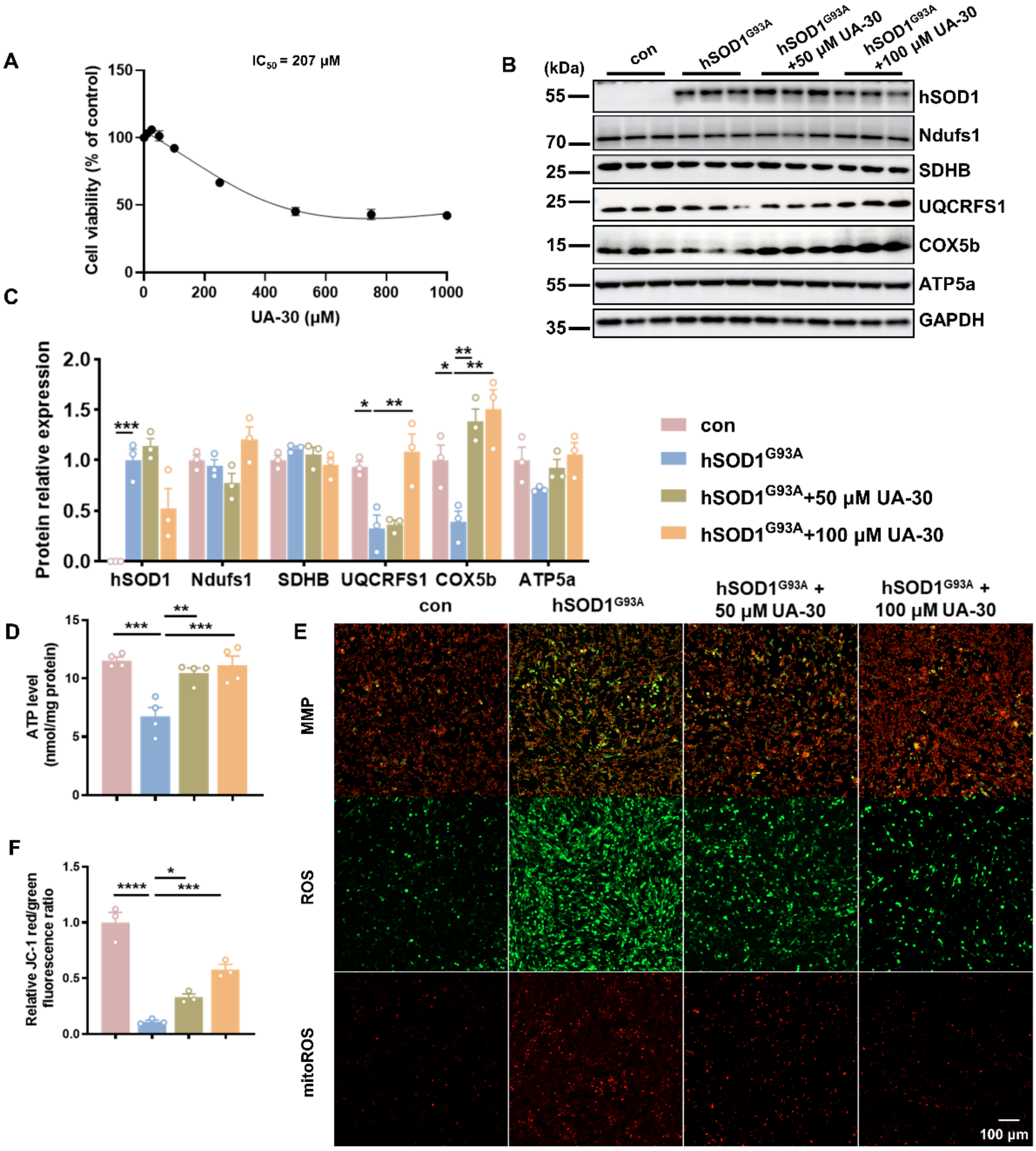

Supplemental Figure 13 UA-30 treatment restored mitochondrial function in $SOD1^{G93A}$ cells.

(A) PC12 cell viability was determined by the CCK-8 assay at different concentrations of UA-30. IC_{50} of UA-30 was calculated to be 207 μM. n = 4 per group. (B, C) Western blot analysis and quantitation of hSOD1 and mitochondrial respiratory complex in $SOD1^{G93A}$-transfected PC12 cells treated with vehicle or UA-30. n = 3 per group. (D) ATP content was measured in UA-30 treated $SOD1^{G93A}$ cells. n = 4 per group. (E) Measurement of MMP level (JC-1 staining red/green fluorescence intensity), ROS

production and mitoROS level in SOD1^{G93A} cells treated with vehicle or UA-30. (F) Quantification of MMP as the JC-1 red/green fluorescence ratio in SOD1^{G93A}-transfected PC12 cells treated with vehicle or UA-30. n = 3 per group. Data are expressed as mean ± SEM. *, $P < 0.05$, **, $P < 0.01$ and ***, $P < 0.001$. One-way ANOVA followed by Dunnett's multiple comparison test.

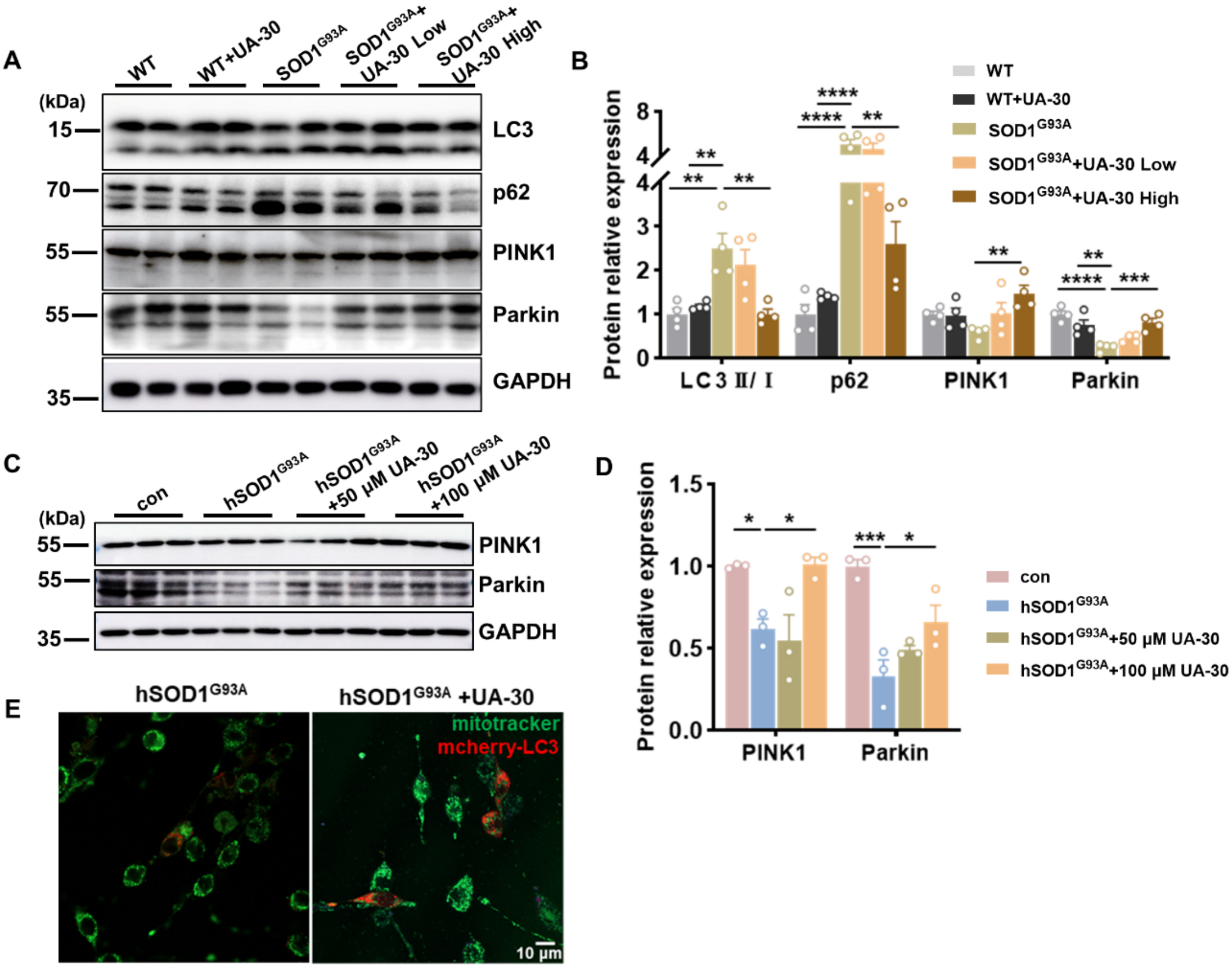

Supplemental Figure 14 UA-30 enhanced PINK1/Parkin-associated mitophagy in the SOD1^{G93A} model.

(A, B) Western blot analysis (A) and quantification (B) for levels of LC3, p62, PINK1 and Parkin in medulla oblongata after UA-30 treatment in SOD1^{G93A} mice. n = 4 male per group. (C, D) WB (C) and quantitation (D) for levels of PINK1 and Parkin after UA-30 treatment in hSOD1^{G93A}-transfected PC12 cells. n = 3 per group. (E) SOD1^{G93A} cells were transfected with mCherry-LC3 for 24 h, then mitochondria were labelled with a mitochondrial dye. After treatment with UA-30, LC3 (red) and mitochondria (green) in the cells were observed using confocal microscopy. Data are expressed as mean ± SEM. *, $P < 0.05$, **, $P < 0.01$ ***, $P < 0.001$ and ****, $P < 0.0001$. One-way ANOVA followed by Dunnett's multiple comparison test.

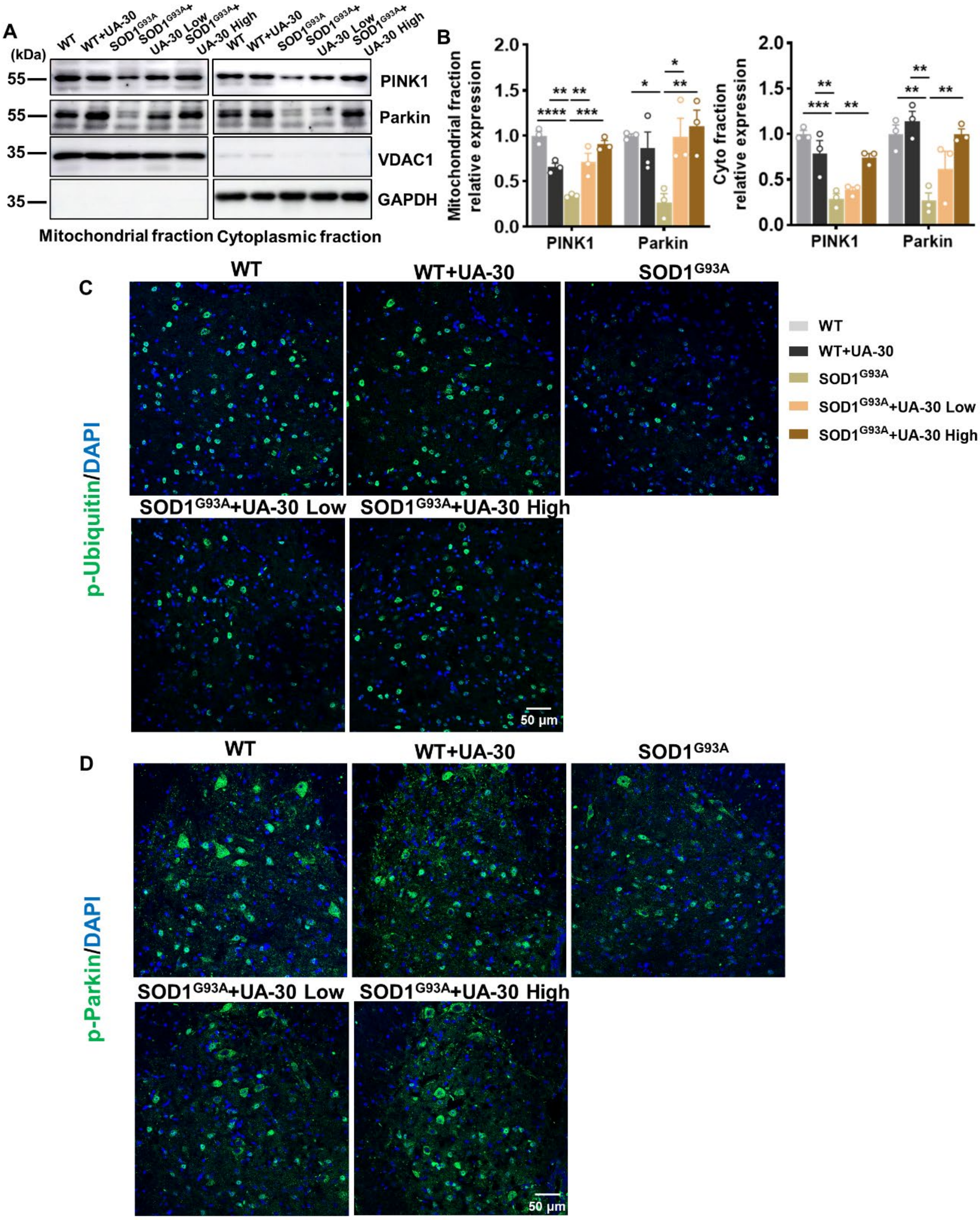

Supplemental Figure 15 UA-30 promoted mitochondrial localization of PINK1/Parkin and restored p-Ubiquitin and p-Parkin signals in the spinal cord of SOD1^{G93A} mice.

(A, B) Western blot analysis and quantification of PINK1 and Parkin levels in

mitochondrial and cytoplasmic fractions isolated from spinal cord of UA-30-treated $SOD1^{G93A}$ mice. VDAC1 was used as a mitochondrial loading control and GAPDH was used as a cytoplasmic loading control. n = 3 male per group. (C) Representative immunofluorescence images of p-Ubiquitin (green) in the ventral horn of the spinal cord from the indicated groups. (D) Representative immunofluorescence images of p-Parkin (green) in the ventral horn of the spinal cord from the indicated groups. Data are expressed as mean ± SEM. *, $P < 0.05$, **, $P < 0.01$, ***, $P < 0.001$ and ****, $P < 0.0001$. One-way ANOVA followed by Dunnett's multiple comparison test.

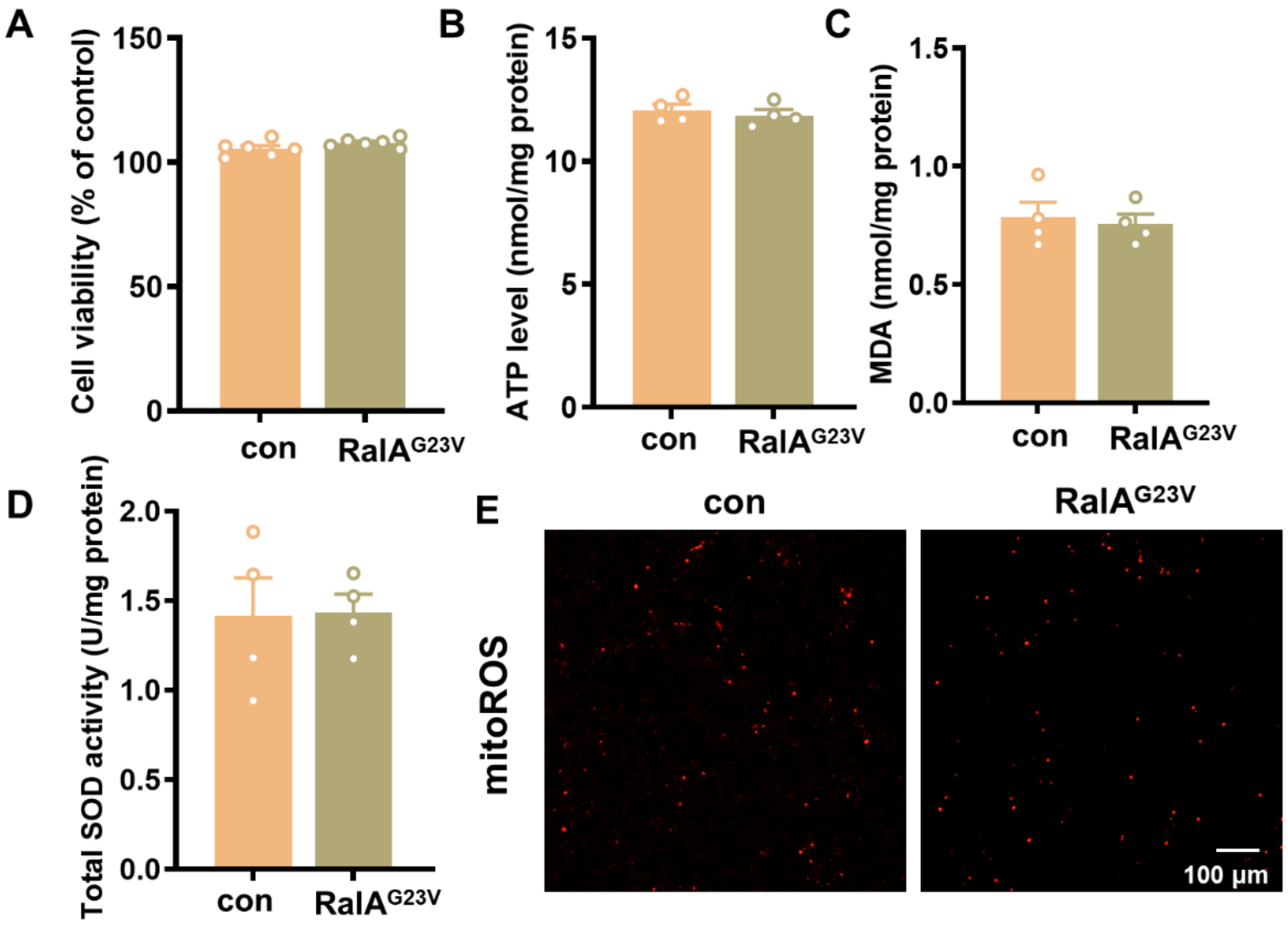

Supplemental Figure 16 Effects of constitutively active RalA in PC12 cells.

(A-D) Measurement of cell viability (A), ATP level (B), MDA level (C), and total SOD activity (D) in PC12 cells transfected with control vector or $RalA^{G23V}$. n = 4-6 per group. (E) Representative images of mitoROS staining in PC12 cells transfected with control vector or $RalA^{G23V}$. Data are presented as mean ± SEM. Two-tailed unpaired Student's t test.

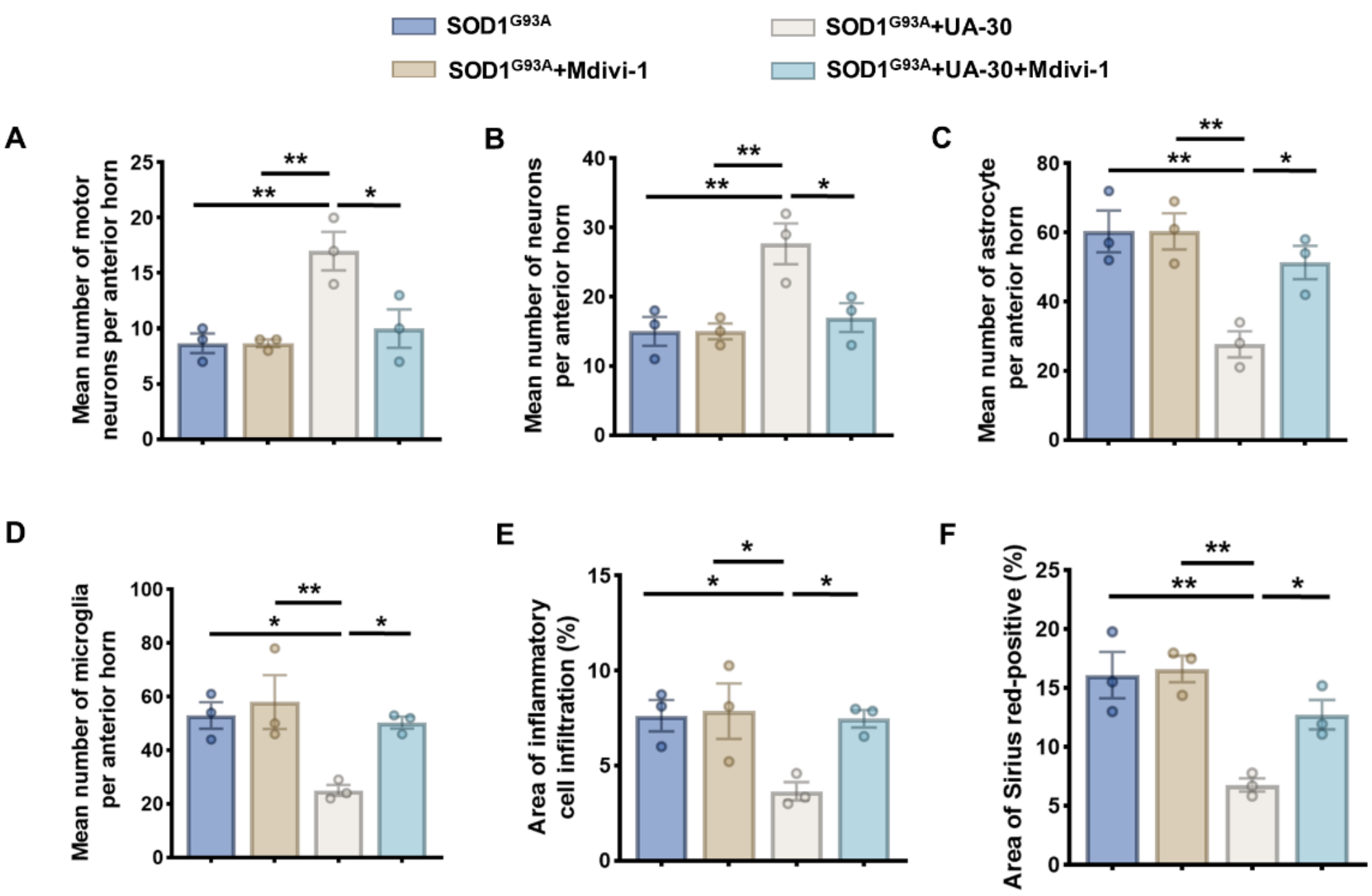

Supplemental Figure 17 Mdivi-1 eliminated the beneficial effects of UA-30 on motor neuron loss and neuroinflammation.

(A-D) Quantitation of Nissl staining (A), NeuN immunohistochemical staining (B), GFAP immunohistochemical staining (C) and Iba1 immunohistochemical staining (D) in spinal cord after UA-30 and Mdivi-1 treatment in SOD1^{G93A} mice. (E, F) Quantitation of histological staining for H&E (E) and Sirius red (F) in the gastrocnemius muscles of SOD1^{G93A} mice exposed to different treatments. n = 3 male per group. Data are expressed as mean ± SEM. *, $P < 0.05$ and **, $P < 0.01$. One-way ANOVA followed by Dunnett's multiple comparison test.

Supplemental Table 1 Pharmacokinetic parameters of UA-30 in brain and spinal cord after oral administration in mice (n=4)

Compound	Tissue	Dose Level (mg/kg)	$T_{1/2}$ (h)	T_{max} (h)	C_{max} (ng/g)	$AUC_{(0-t)}$ (h*ng/g)	$AUC_{(0-\infty)}$ (h*ng/g)	$MRT_{(0-t)}$ (h)	$MRT_{(0-\infty)}$ (h)
UA-30	brain	100	3.44±0.248	0.875±0.25	16490±2204	85188±12814	85703±12737	4.46±0.352	4.62±0.361
	spinal cord	100	3.67±0.177	0.625±0.25	6293±1022	20426±2104	20549±2103	3.75±0.354	3.9±0.347

Supplemental Table 2 Antibodies utilized in this study

Antibody	Catalog No.	Host	Dilution	Source
SOD1	ab52950	Rabbit	1:5000	Abcam
GFAP	3670	Mouse	1:200	Cell Signaling Technology
Iba1	019-19741	Rabbit	1:200	Wako
NeuN	MAB377	Mouse	1:200	Sigma-Aldrich
ChAT	PA5-97583	Rabbit	1:100	Invitrogen
Ndufs1	ab198955	Rabbit	1:1000	Abcam
SDHB	ab14714	Mouse	1:1000	Abcam
UQCRFS1/RISP	ab131152	Rabbit	1:1000	Abcam
COX5b	ab180136	Rabbit	1:1000	Abcam
LC3A/B	12741	Rabbit	1:500	Cell Signaling Technology
Lamp2	66301-1-Ig	Mouse	1:1000	Proteintech
SQSTM/p62	ab109012	Rabbit	1:1000	Abcam
Tomm40	sc-365467	Mouse	1:1000	Santa Cruz
Tomm20	ab186734	Rabbit	1:1000	Abcam
Ubiquitin	ab19247	Rabbit	1:1000	Abcam
ERK1/2	16443-1-AP	Rabbit	1:1000	Proteintech
p-ERK1/2	4370	Rabbit	1:1000	Cell Signaling Technology
FOXO3a	12829	Rabbit	1:1000	Cell Signaling Technology
p-FOXO3a (Ser294)	5538	Rabbit	1:500	Cell Signaling Technology
Histone H3	ab1791	Rabbit	1:1000	Abcam
RalA	13629-1-AP	Rabbit	1:1000	Proteintech
PINK1	sc-517353	Mouse	1:1000	Santa Cruz
Parkin	sc-32282	Mouse	1:1000	Santa Cruz
GAPDH	5174	Rabbit	1:3000	Cell Signaling Technology

VDAC1	ab154856	Rabbit	1:1000	Abcam
p-Ubiquitin	AF3888	Rabbit	1:200	Affinity Biosciences
p-Parkin	AF3500	Rabbit	1:200	Affinity Biosciences
Synaptophysin	17785-1-AP	Rabbit	1:200	Proteintech

Supplemental Table 3 Primer sequences used for RT-qPCR in this study

Gene	Forward (5’-3’)	Reverse (5’-3’)
Pink1	TTCTTCCGCCAGTCGGTAG	CTGCTTCTCCTCGATCAGCC
Prkn	AAACCGGATGAGTGGTGAGT	AGCTACCGACGTGTCCTTGT
Foxo3a	GGGGAACCTGTCCTATGCC	TCATTCTGAACGCGCATGAAG
Rala	ACAGGATGGCTGCAAACAAG	TGAACTGCAGAGTCAGAGCA
Gapdh	CATCACTGCCACCCAGAAGACTG	ATGCCAGTGAGCTTCCCGTTCAG

Supplemental Video legends

Supplemental Video 1. Representative gait test videos of male $SOD1^{G93A}$ mice after UA-30 treatment.

Representative gait test videos show locomotor patterns of male mice treated with UA-30, Riluzole, or saline. WT and WT+UA-30 mice are included as controls. Videos are arranged by treatment group as indicated.

Supplemental Video 2. Representative gait test videos of female $SOD1^{G93A}$ mice after UA-30 treatment.

Representative gait test videos show locomotor patterns of female mice treated with UA-30, Riluzole, or saline. WT and WT+UA-30 mice are included as controls. Videos are arranged by treatment group as indicated.

Supplemental Video 3. Representative gait test videos of male $SOD1^{G93A}$ mice after UA-30 with or without Mdivi-1 treatment.

Representative gait test videos show locomotor patterns of male $SOD1^{G93A}$ mice treated with vehicle, Mdivi-1, UA-30, or UA-30 plus Mdivi-1. Videos are arranged by treatment group as indicated.
