# Supplementary material for: UA-30 ameliorates motor deficits through RalA-mediated mitophagy in ALS mice
Source: J Clin Invest. 2026 Sep 15;136(18):e202787. doi: 10.1172/JCI202787 (PMC13574146; doi:10.1172/JCI202787)

Full unedited blot for Figure 5A

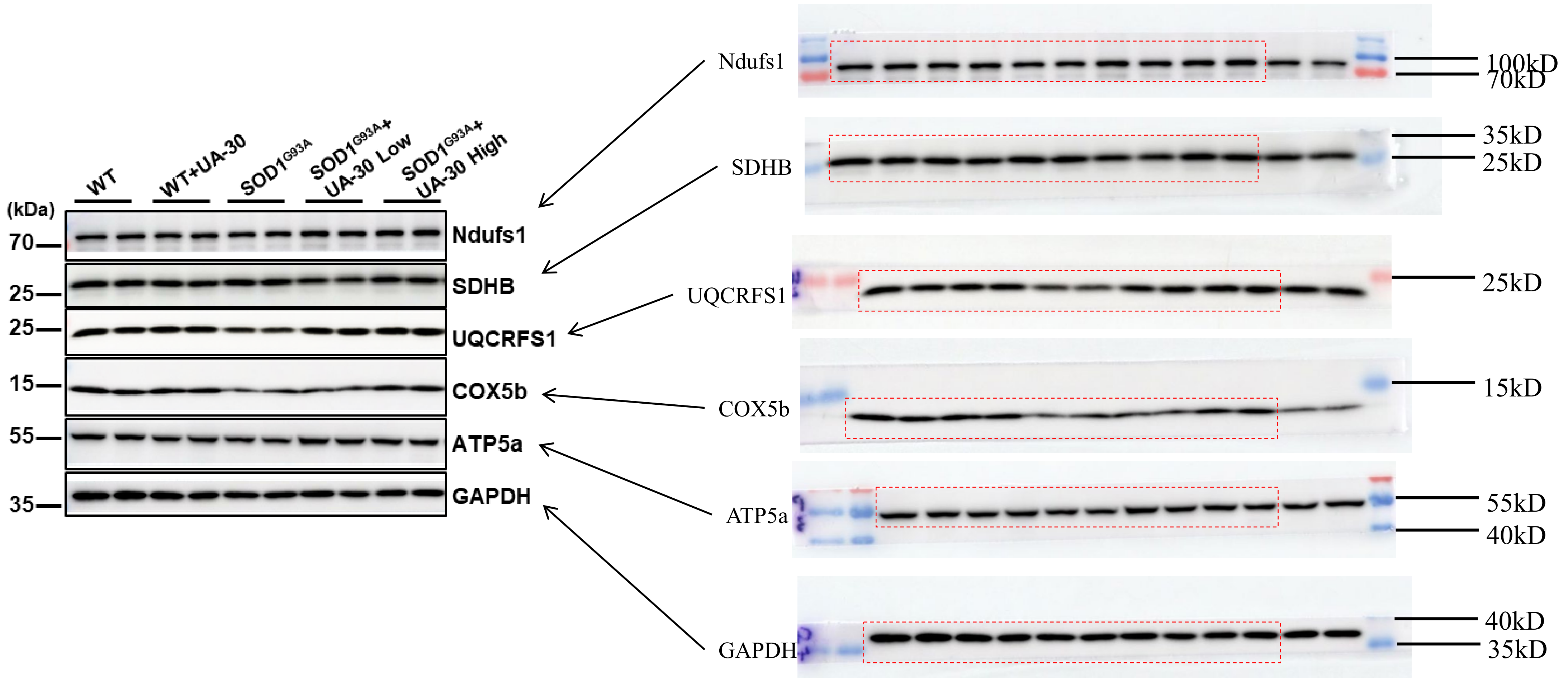

Full unedited blot for Figure 5C

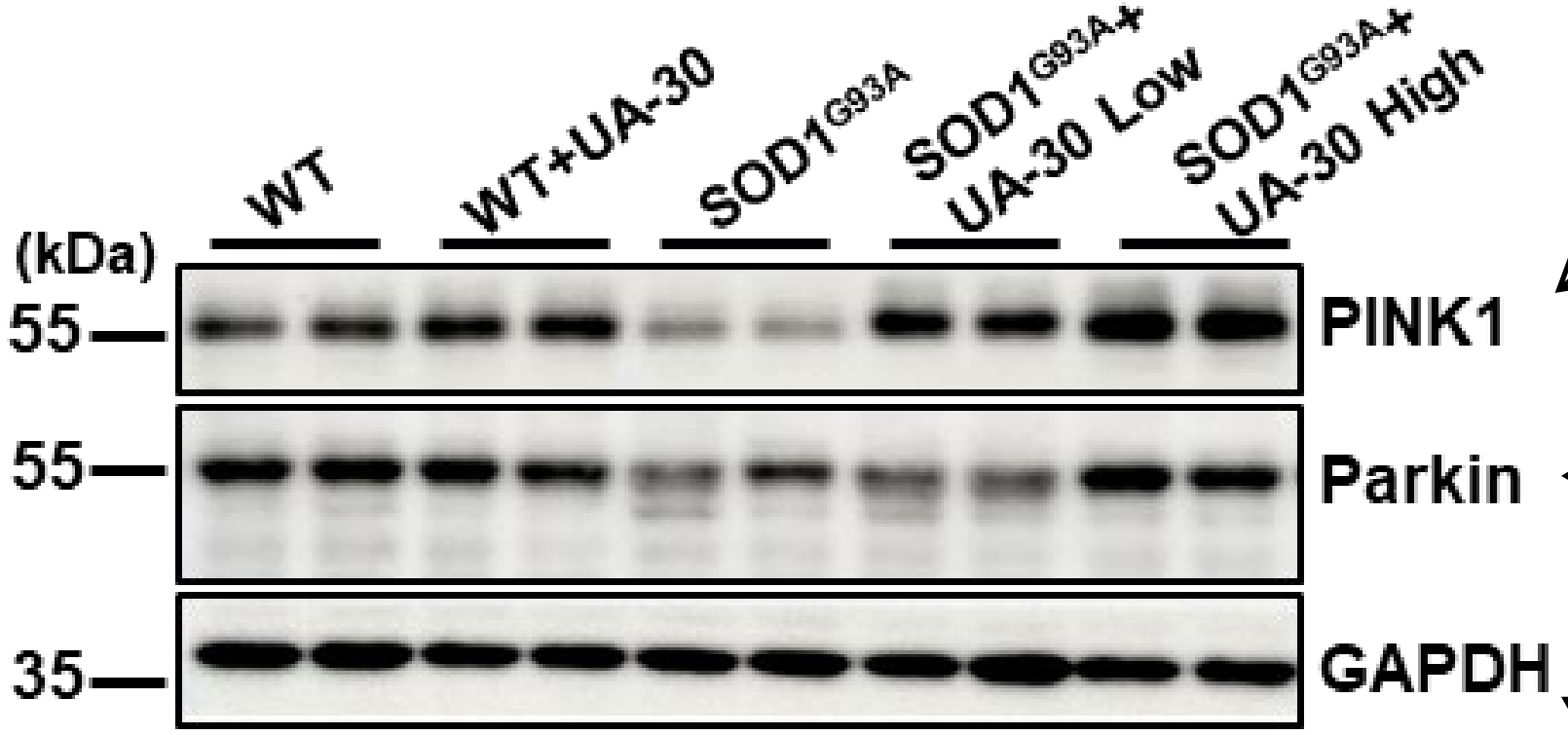

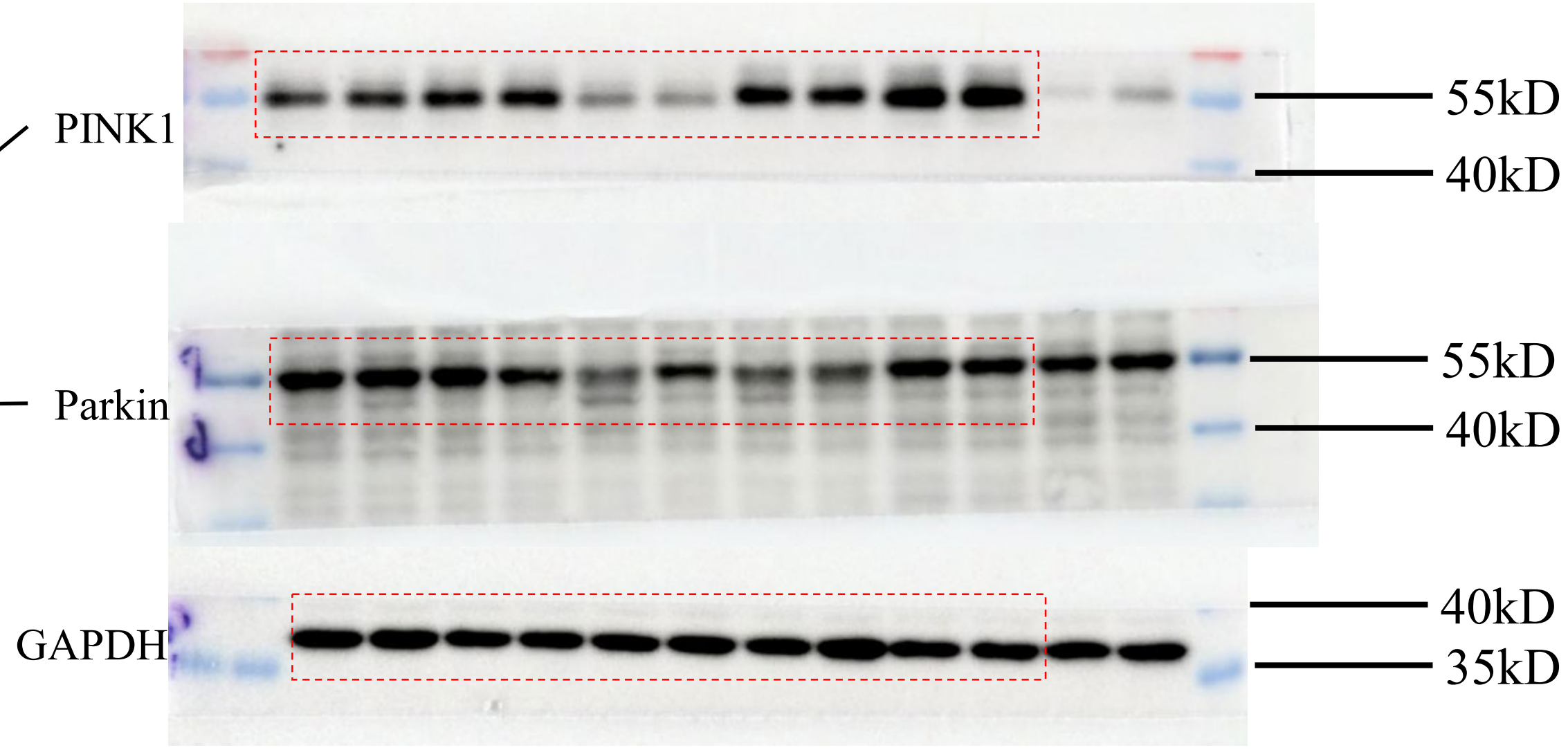

Full unedited blot for Figure 6A

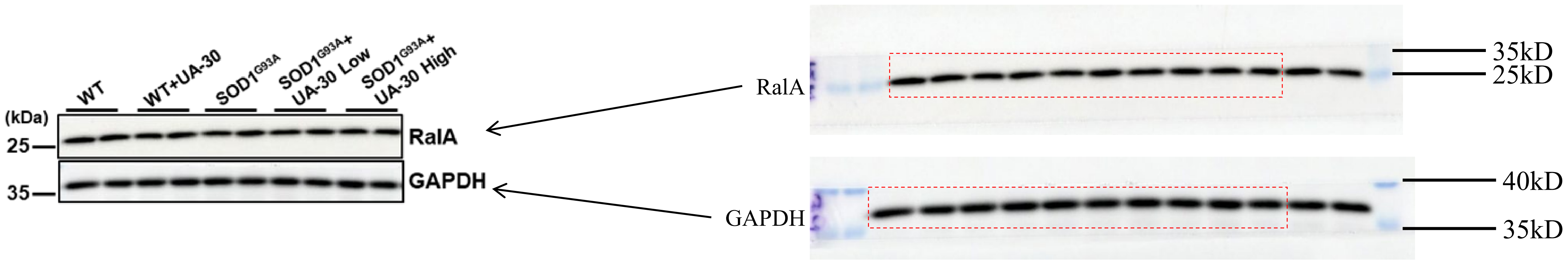

Full unedited blot for Figure 6E

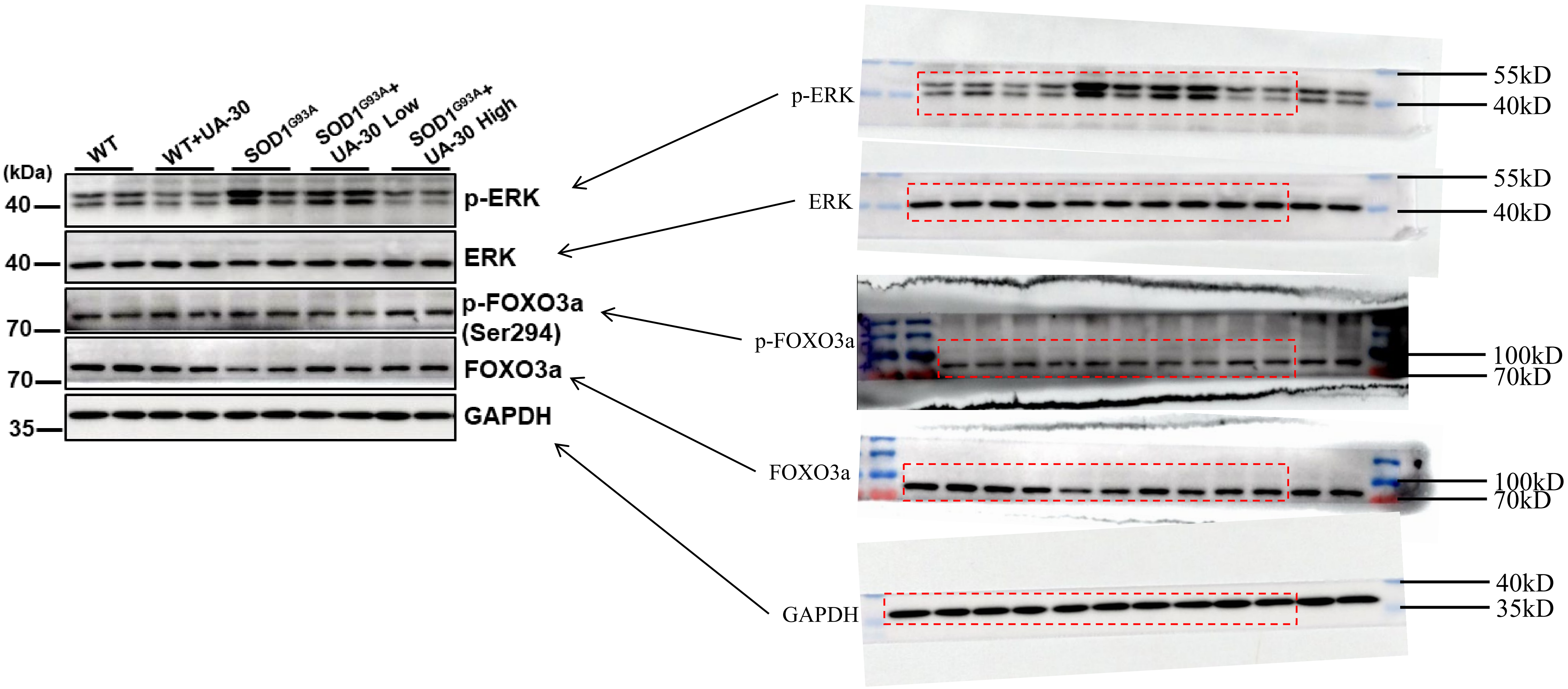

Full unedited blot for Figure 6G

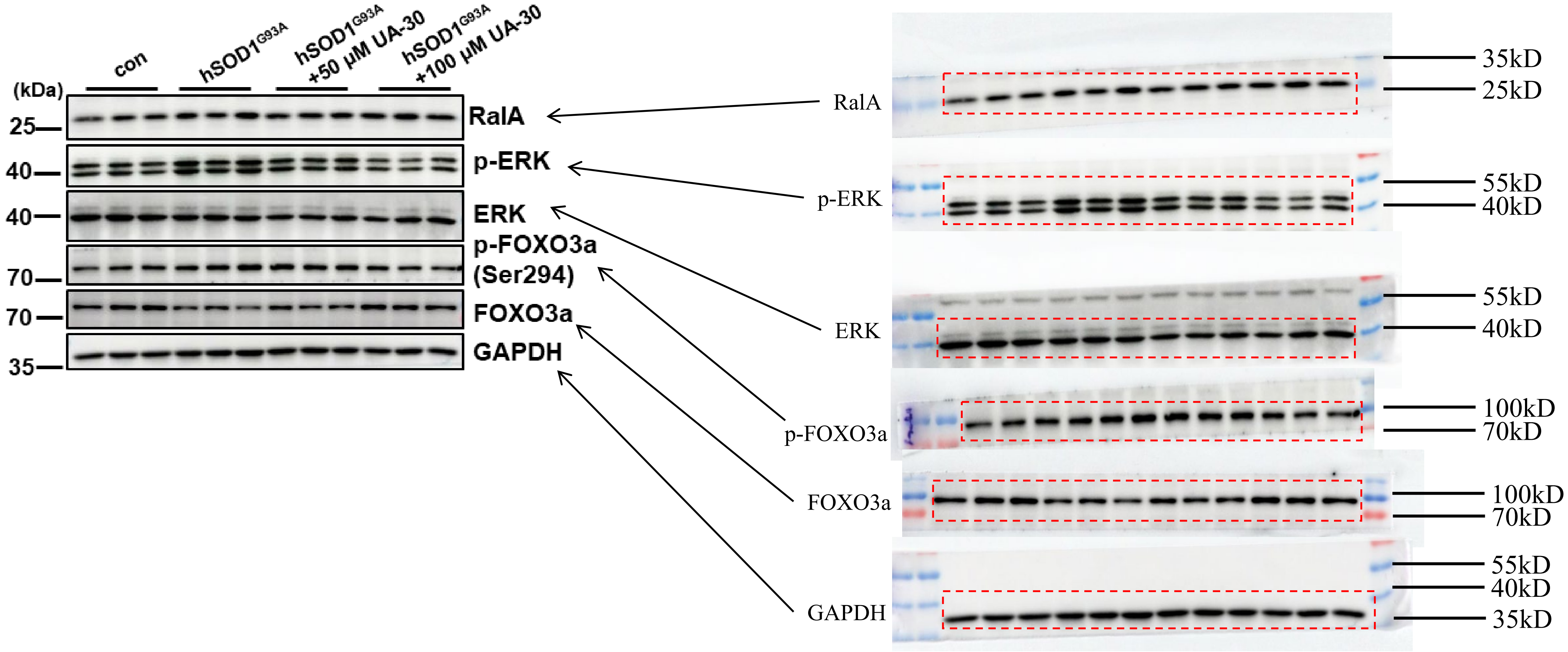

Full unedited blot for Figure 6J

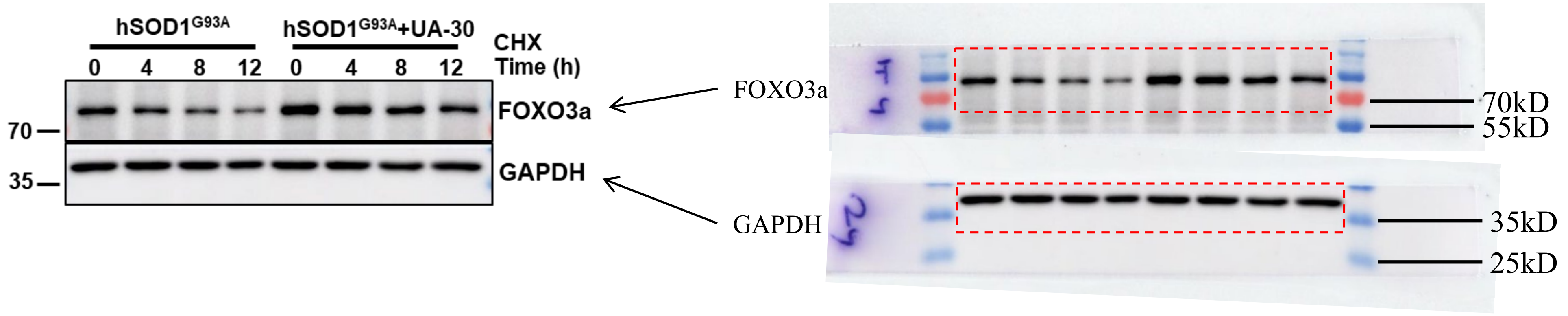

Full unedited blot for Figure 6L

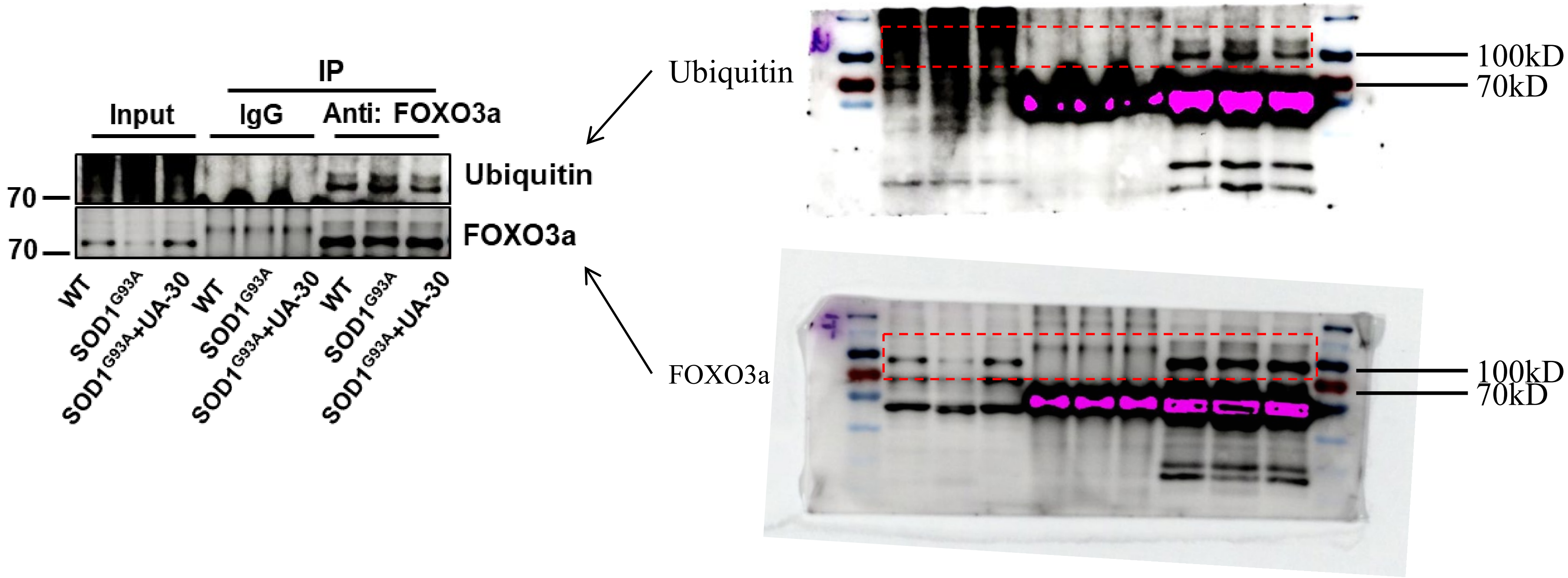

Full unedited blot for Figure 6M

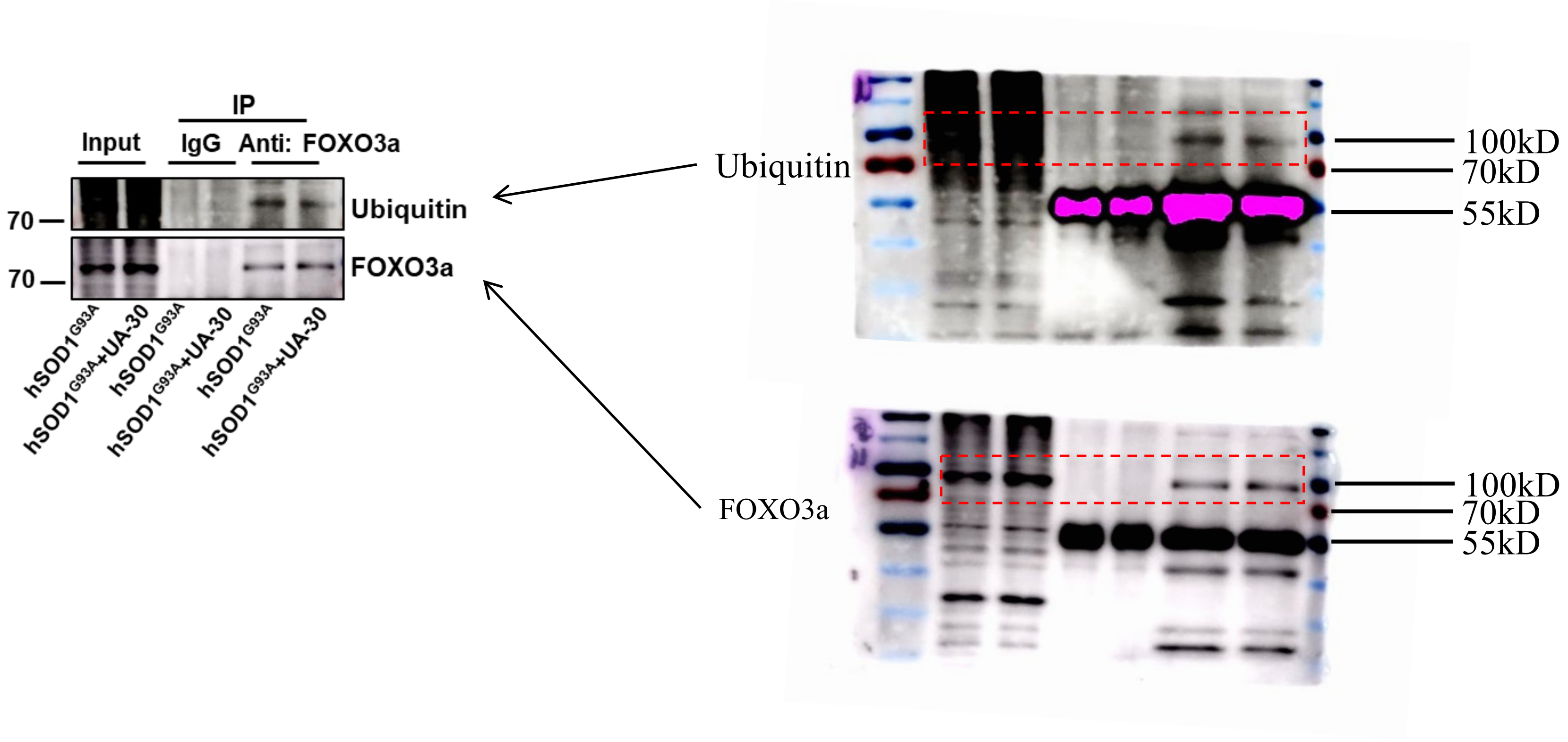

Full unedited blot for Figure 6N

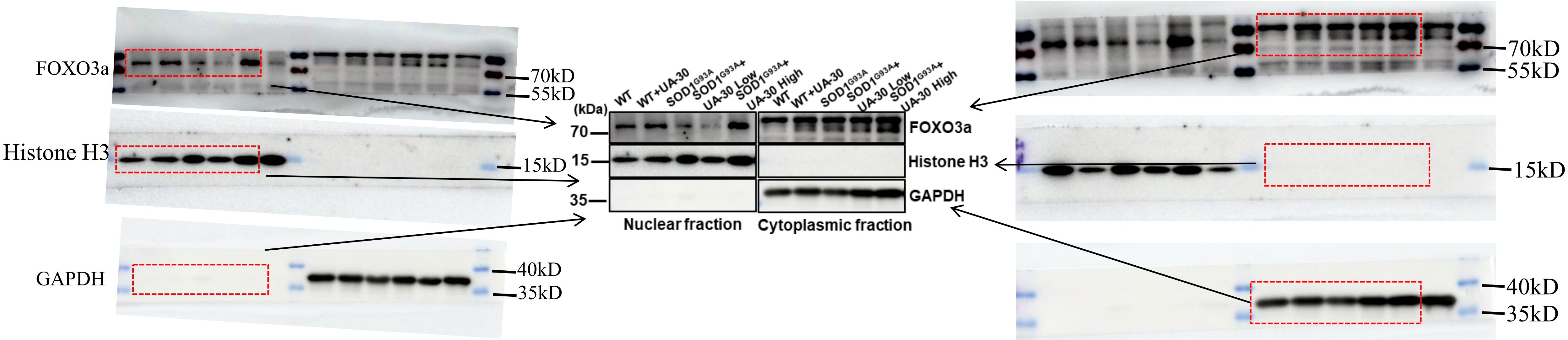

Full unedited blot for Figure 6O

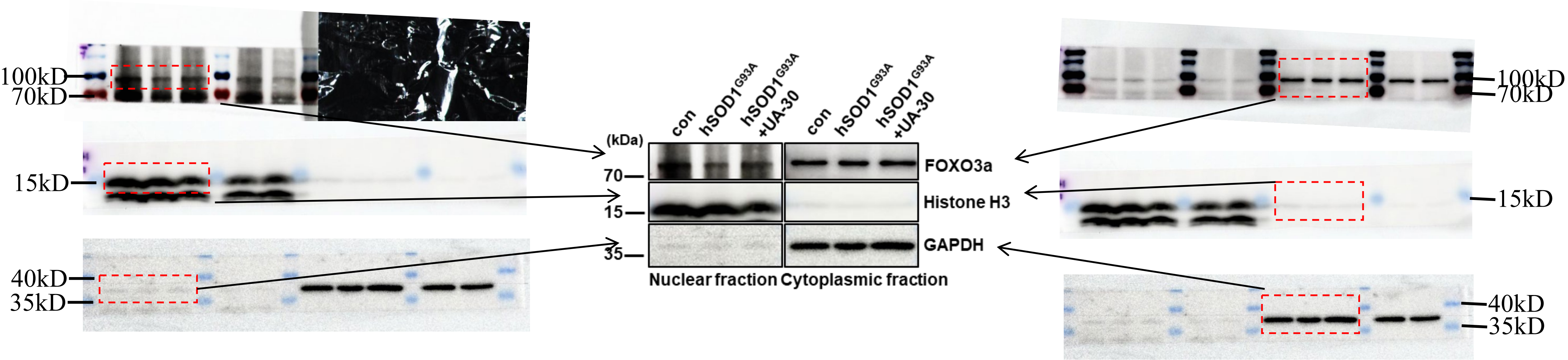

Full unedited blot for Figure 7A

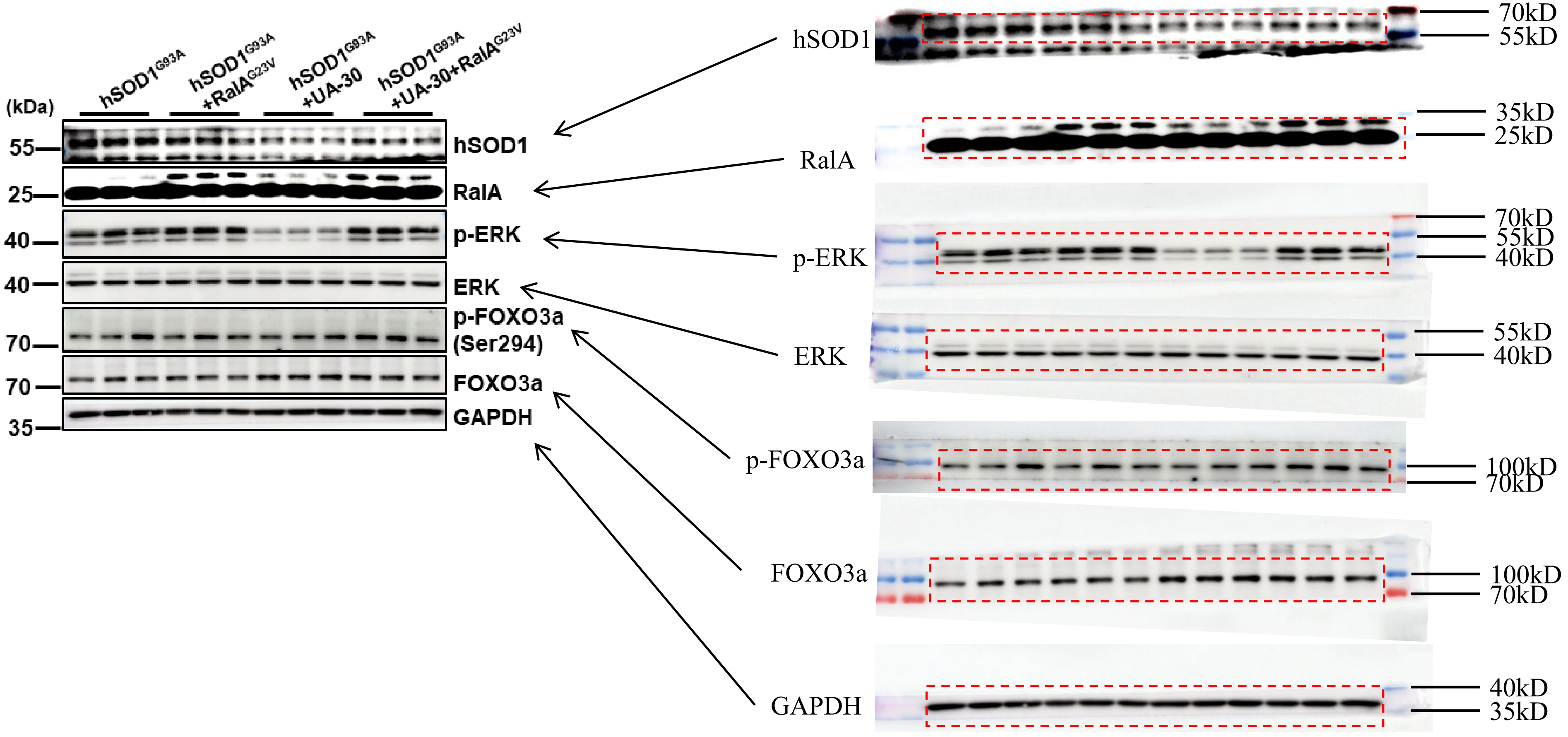

Full unedited blot for Figure 7C

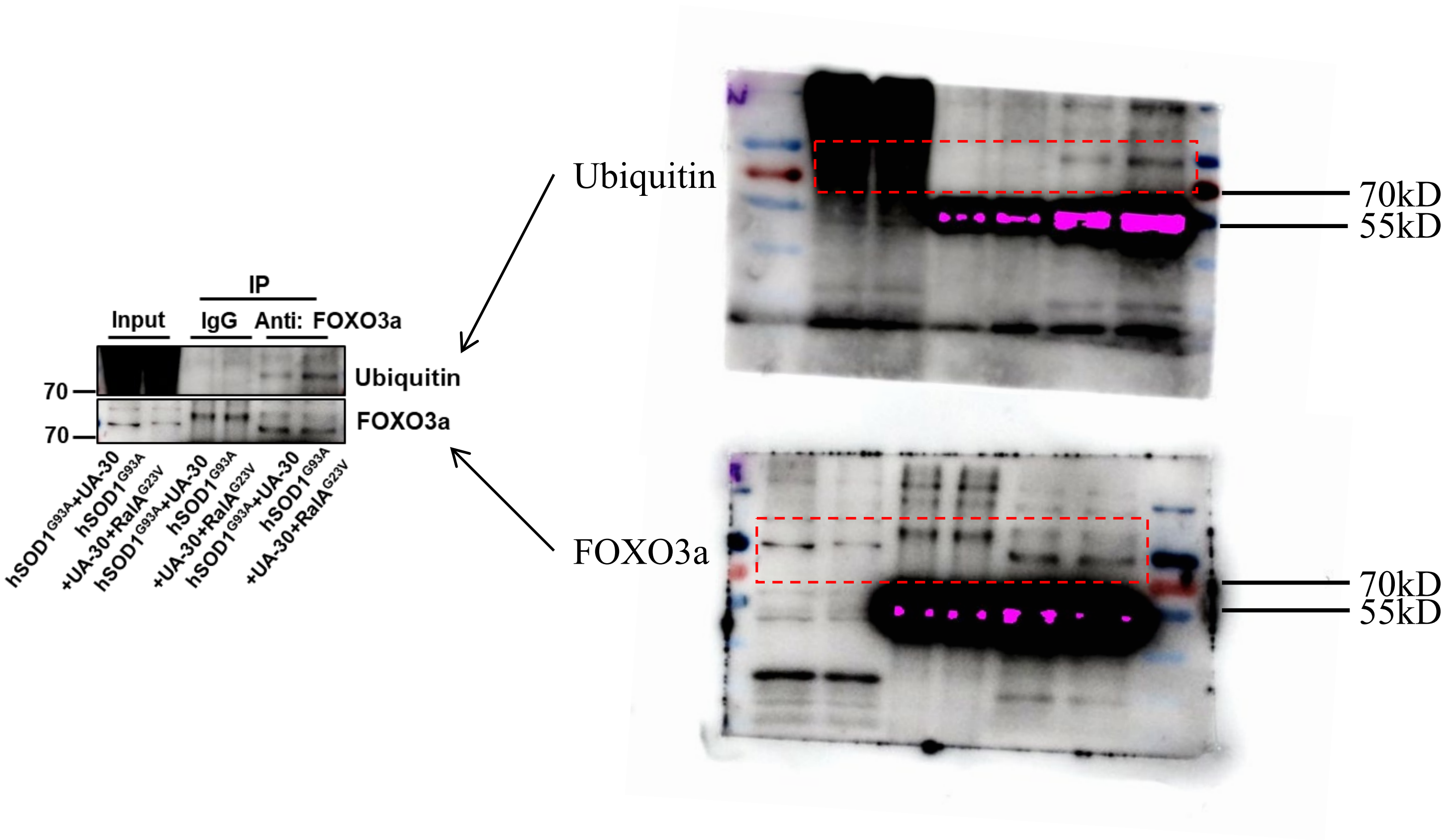

Full unedited blot for Figure 7D

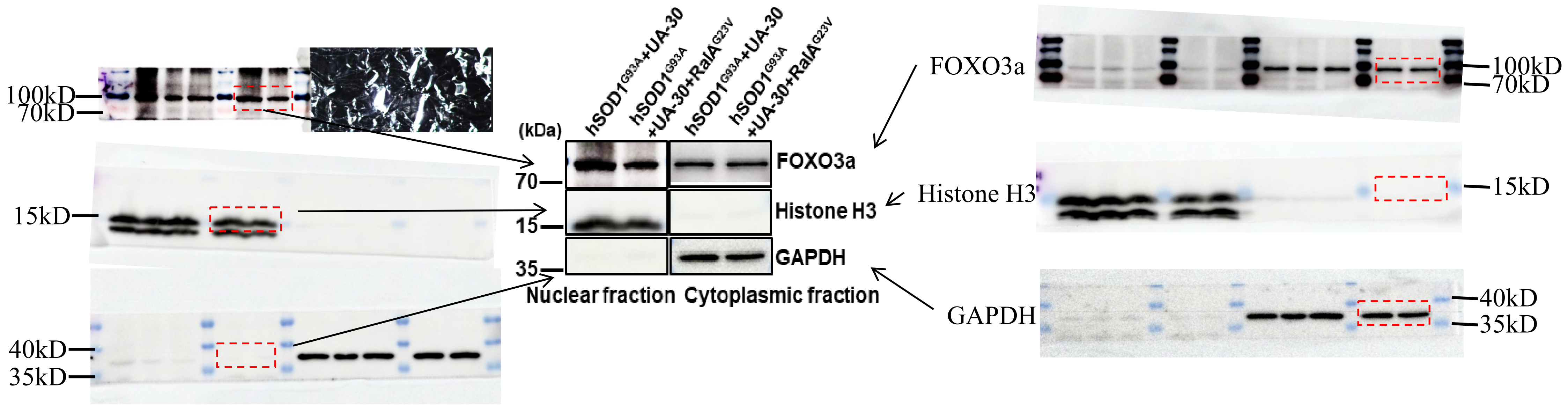

Full unedited blot for Figure 7E

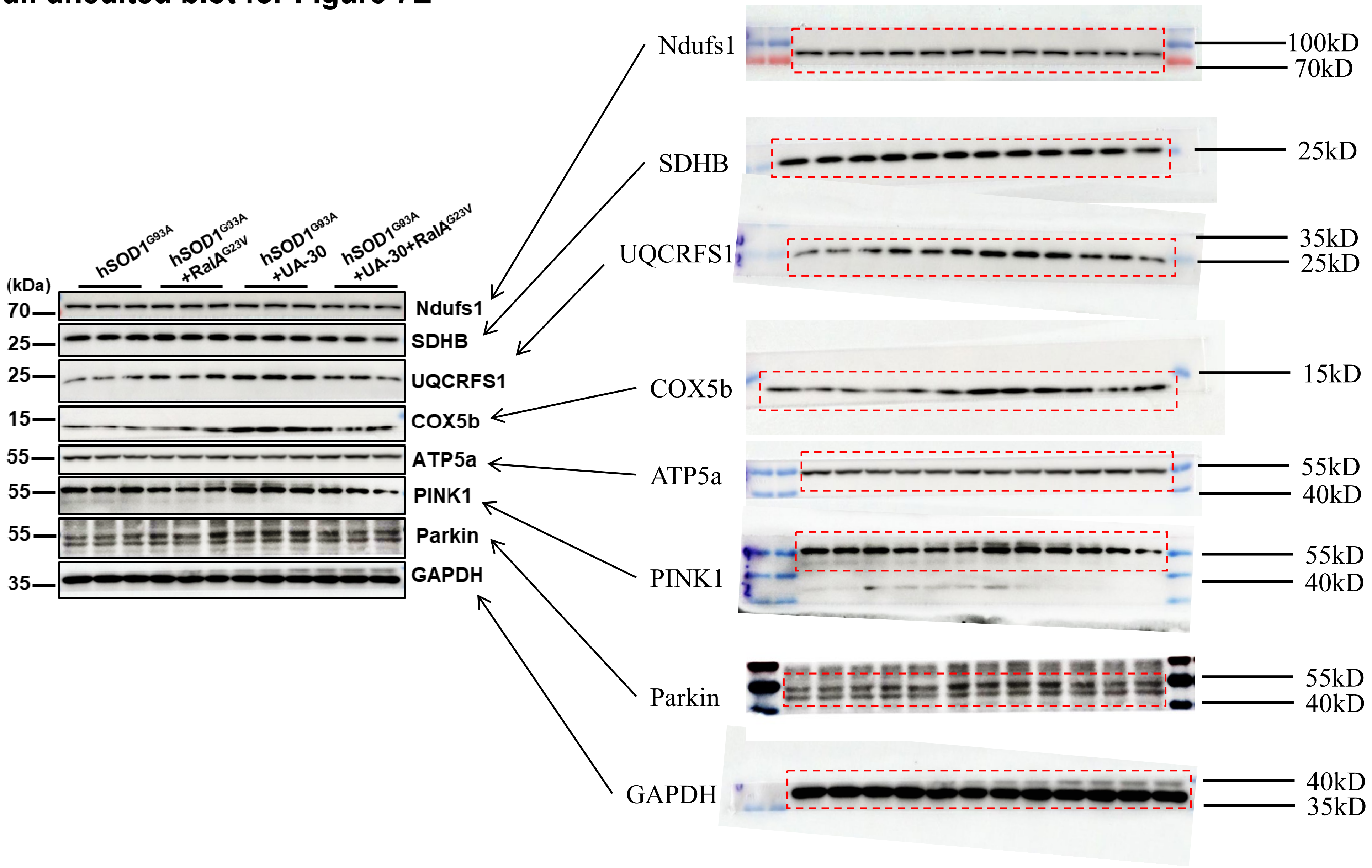

Full unedited blot for Figure 8A

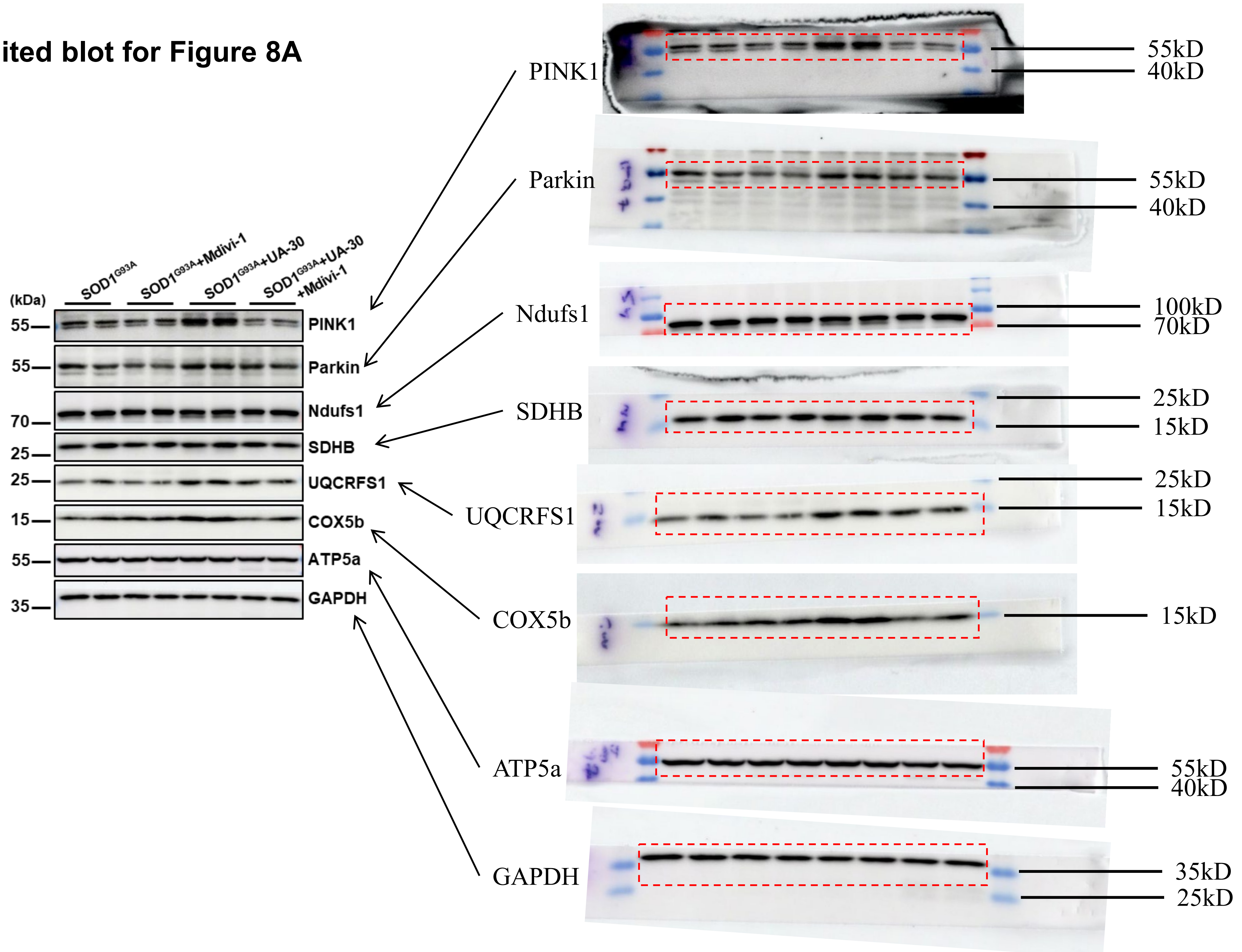

Full unedited blot for Supplemental Figure 11B

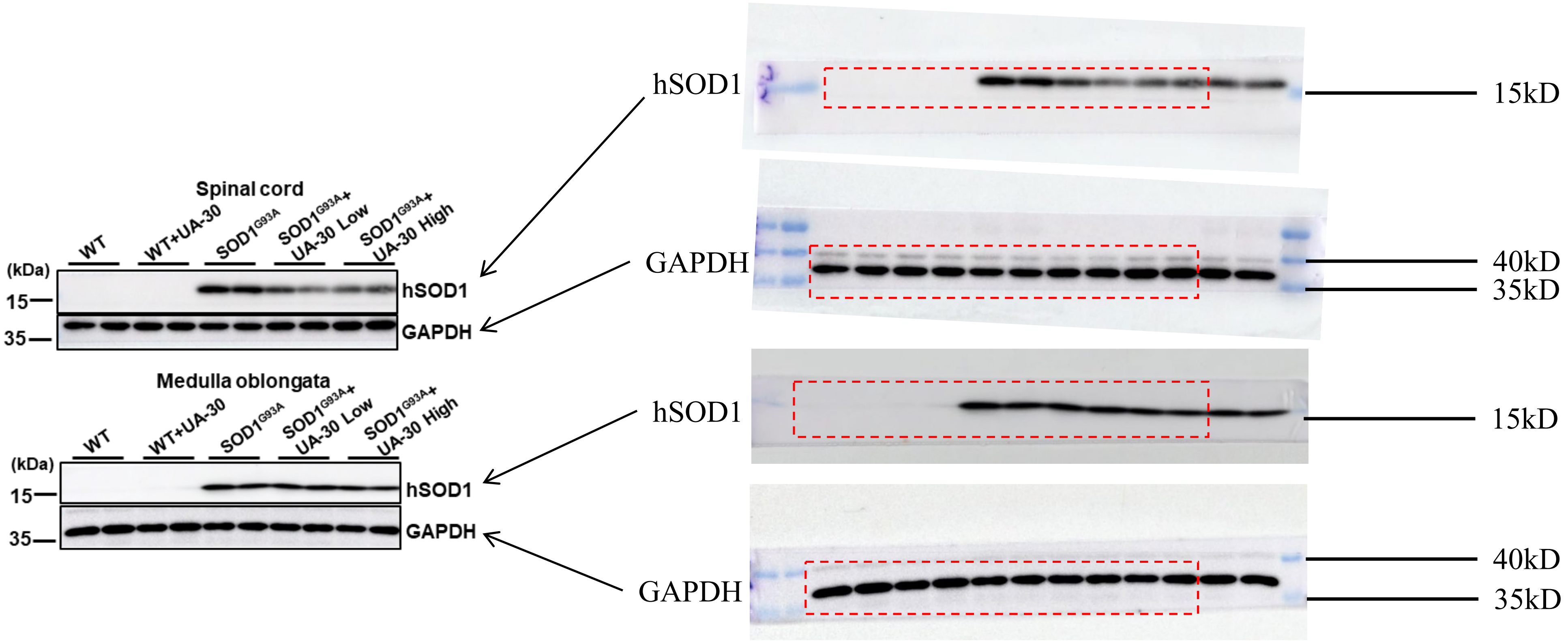

Full unedited blot for Supplemental Figure 13A

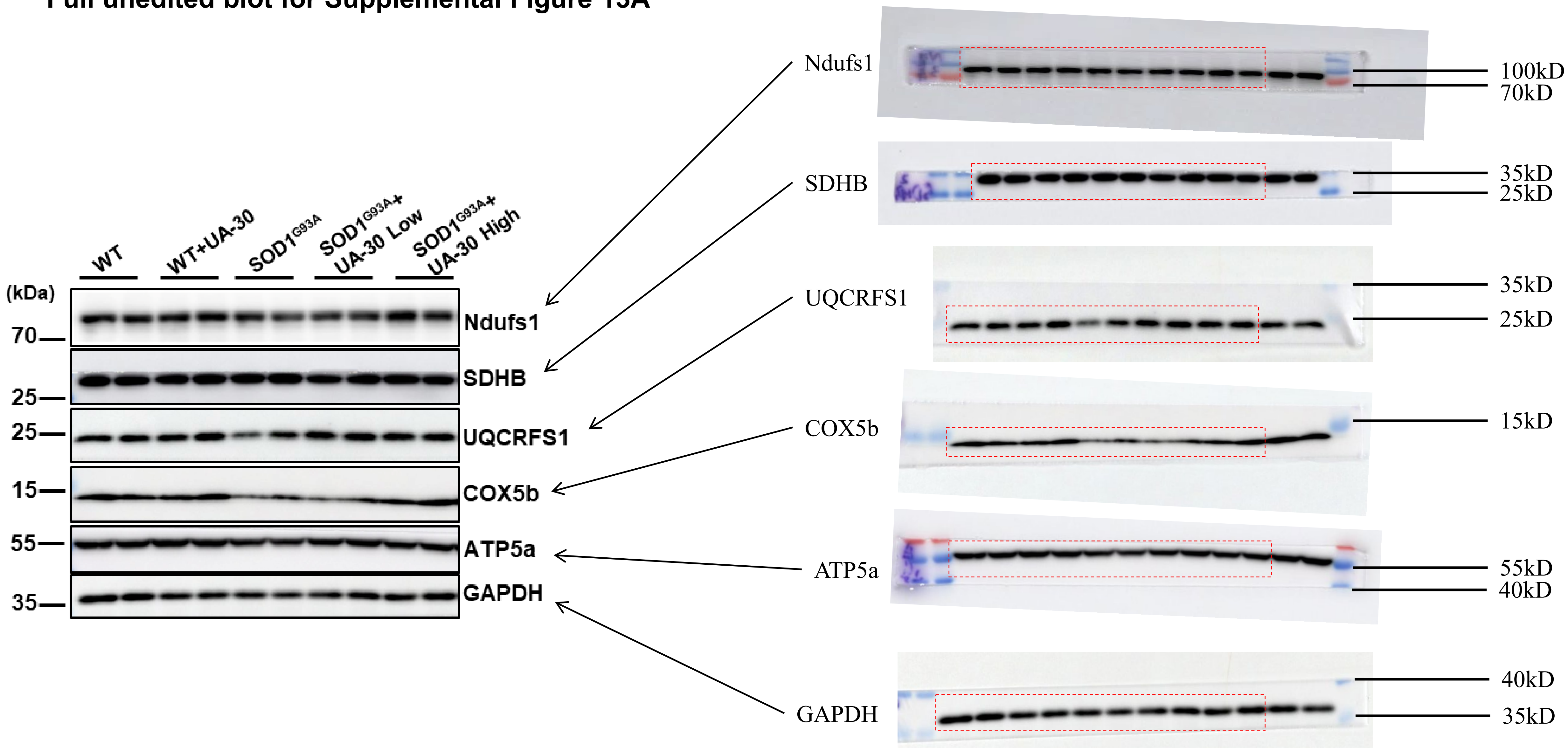

Full unedited blot for Supplemental Figure 14B

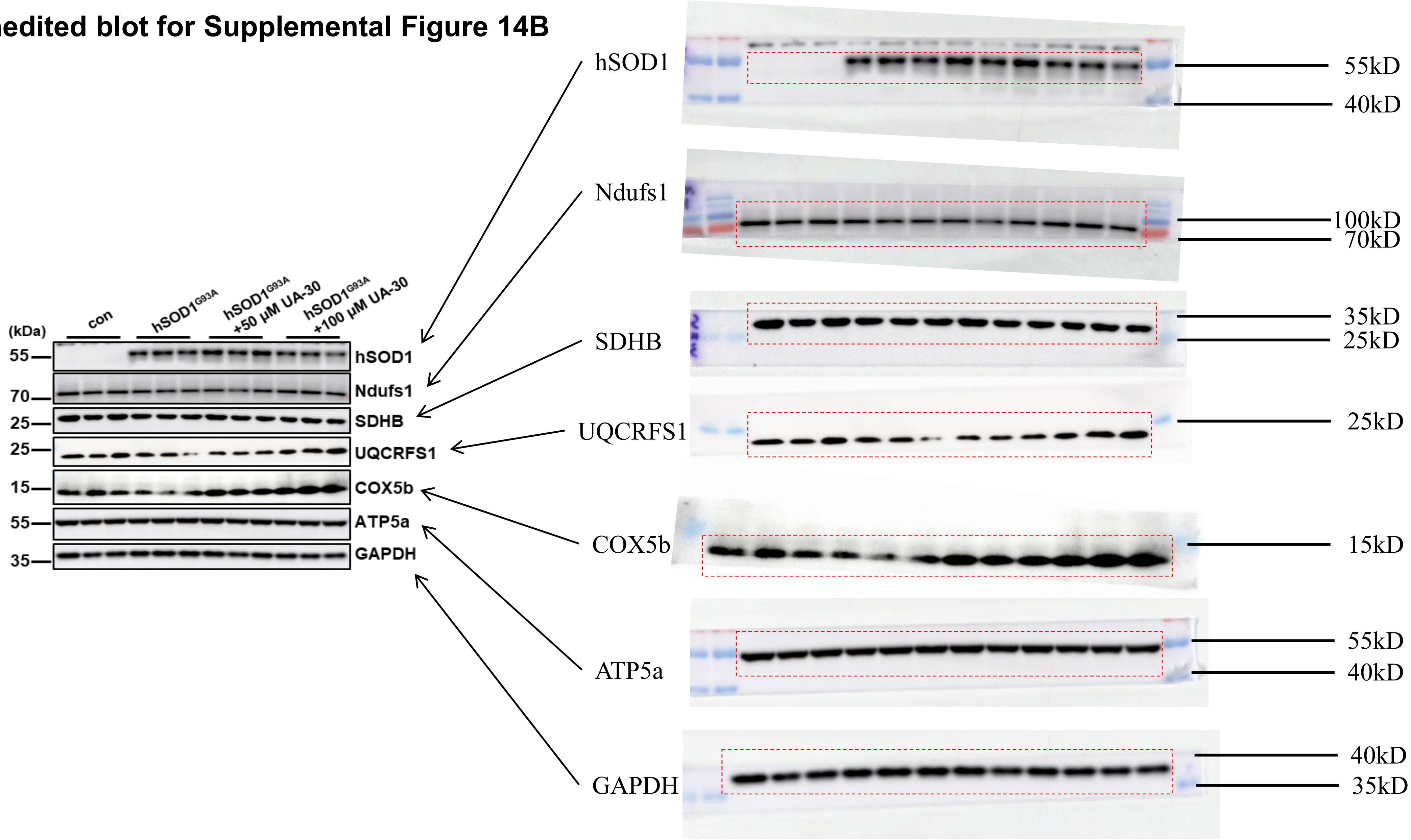

Full unedited blot for Supplemental Figure 15A

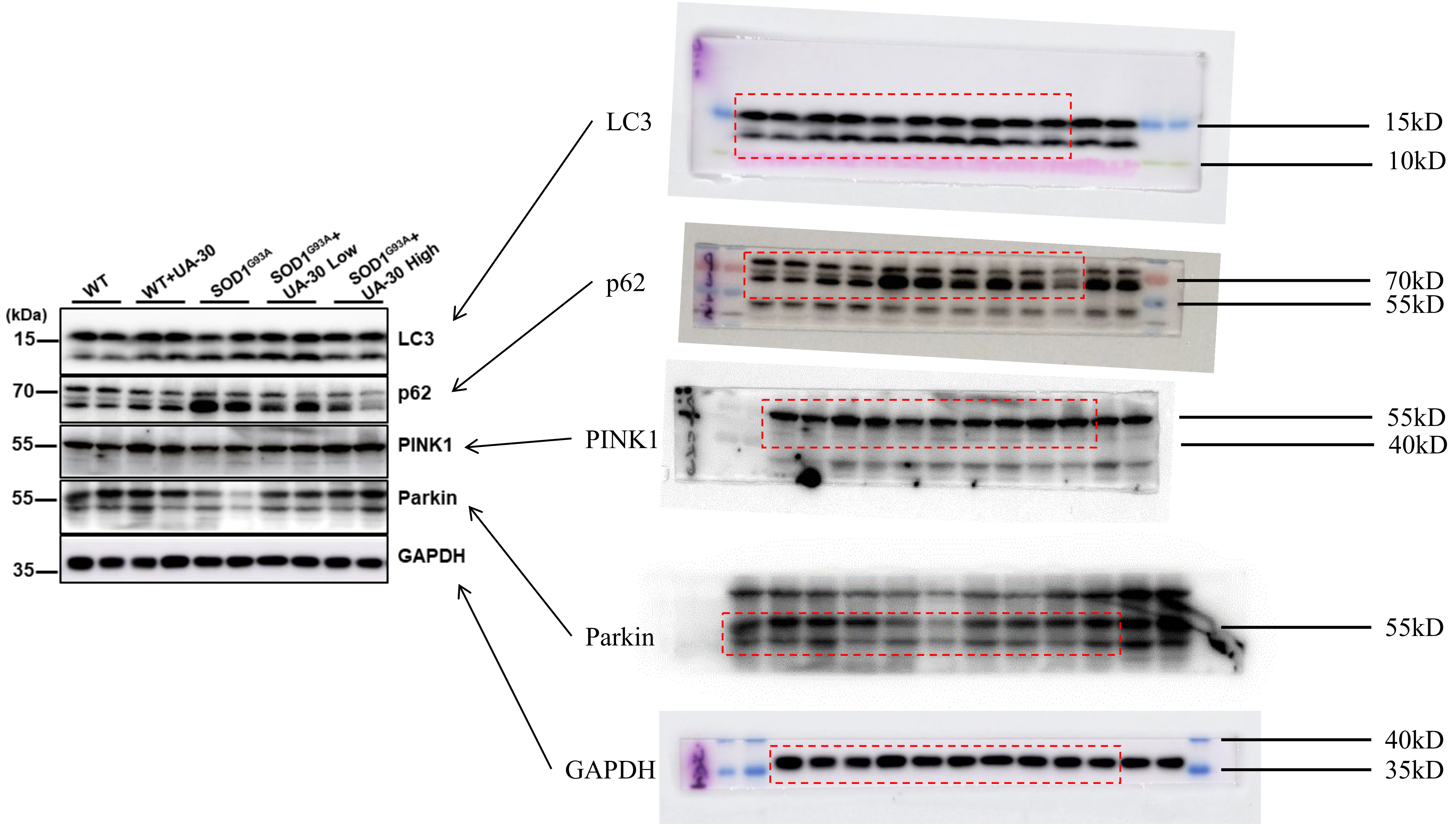

Full unedited blot for Supplemental Figure 15C

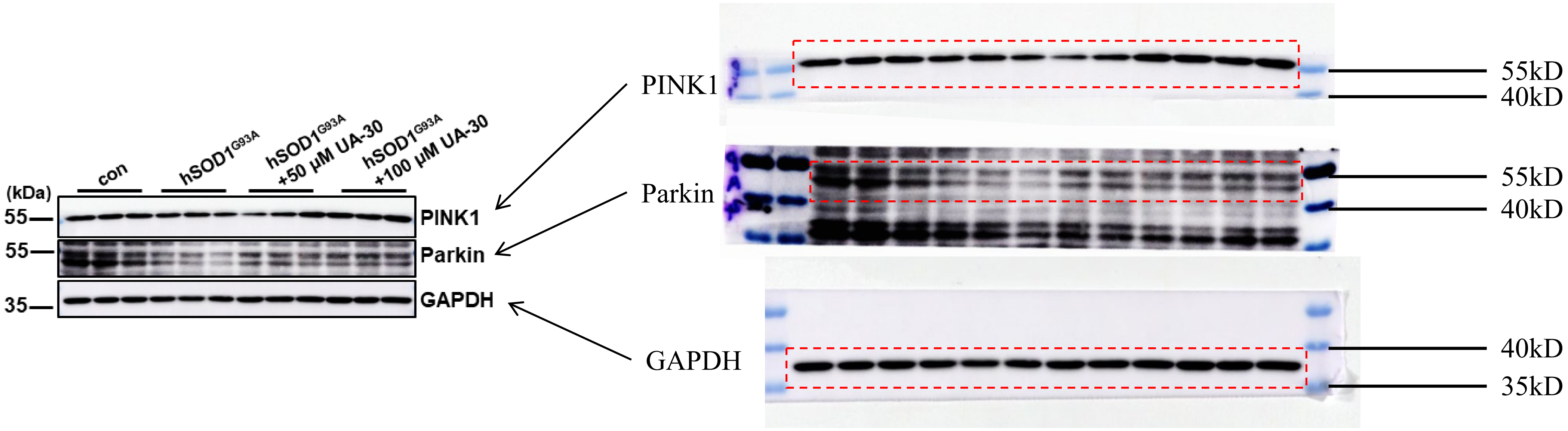

Full unedited blot for Supplemental Figure 16A

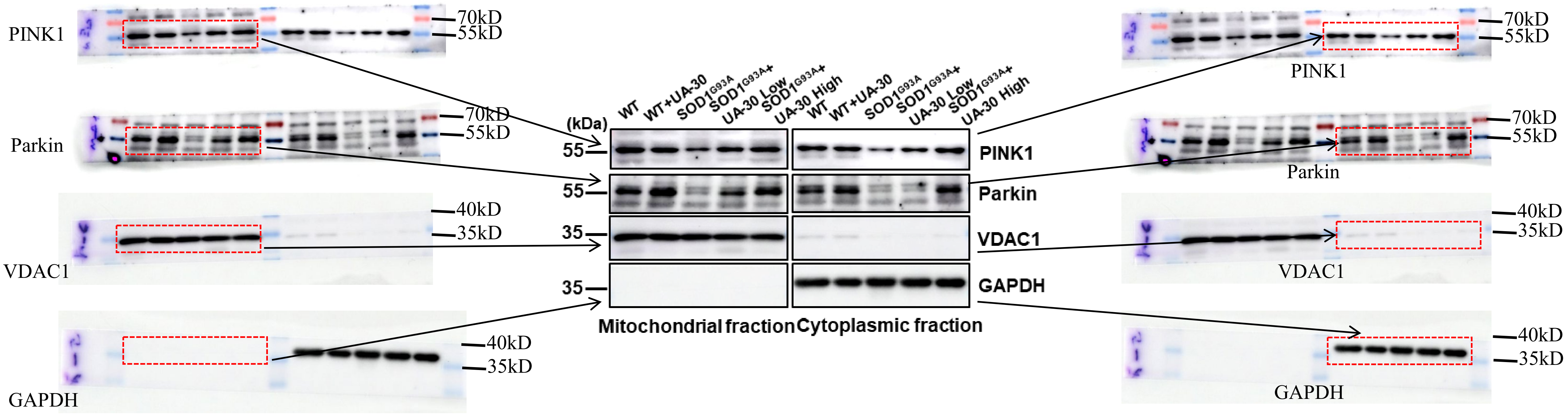

Supplement: Supplementary file 2 [file jci-136-202787-s090.pdf]
